# Peripherally restricted transthyretin-based delivery system for probes and therapeutics avoiding opioid-related side effects

**DOI:** 10.1038/s41467-022-31342-z

**Published:** 2022-06-23

**Authors:** Md Tariqul Haque Tuhin, Dengpan Liang, Fang Liu, Hala Aldawod, Toufiq Ul Amin, Joshua S. Ho, Rasha Emara, Arjun D. Patel, Melanie A. Felmlee, Miki S. Park, James A. Uchizono, Mamoun M. Alhamadsheh

**Affiliations:** Department of Pharmaceutics and Medicinal Chemistry, Thomas J. Long School of Pharmacy, University of the Pacific, Stockton, CA 95211 US

**Keywords:** Drug delivery, Small molecules, Pharmacology, Mechanism of action

## Abstract

Several investigations into the sites of action of opioid analgesics have utilized peripherally acting mu-opioid receptor antagonists (PAMORAs), which have been incorrectly assumed to possess limited permeability across the blood-brain barrier. Unfortunately, the poor pharmacokinetic properties of current PAMORAs have resulted in misunderstandings of the role of central nervous system and gastrointestinal tract in precipitating side effects such as opioid-induced constipation. Here, we develop a drug delivery approach for restricting the passage of small molecules across the blood-brain barrier. This allows us to develop naloxone- and oxycodone-based conjugates that display superior potency, peripheral selectivity, pharmacokinetics, and efficacy in rats compared to other clinically used PAMORAs. These probes allow us to demonstrate that the mu-opioid receptors in the central nervous system have a fundamental role in precipitating opioid-induced constipation. Therefore, our conjugates have immediate use as pharmacological probes and potential therapeutic agents for treating constipation and other opioid-related side effects.

## Abstract (web summary)

The current peripherally acting mu-opioid receptor antagonists present limited permeability and pharmacokinetic properties. Here, the authors develop a drug delivery approach based on AG10, which demonstrates the impact of mu-opioid receptors in the central nervous system in precipitating opioid-induced constipation.

## Introduction

There are several strategies for delivering therapeutics across the blood-brain barrier (BBB)^1^. However, there are limited approaches for restricting the passage of molecules across the BBB. Such an approach would be useful for limiting the central nervous system (CNS) toxicity of certain therapeutics with peripheral targets (e.g., ciprofloxacin, methotrexate, and indomethacin)^2–4^. Another application of this approach would be to develop pharmacological probes that could be used to investigate the peripheral vs. CNS action of drug molecules such as opioids. To limit BBB penetration, a drug molecule is typically modified to increase its size and hydrophilicity. However, this often adversely affects the potency and pharmacokinetic properties of the drug molecule.

Here we describe the development of an approach that limits the undesired diffusion of molecules across the BBB. Our strategy involves endowing the therapeutic agents with a hydrophilic derivative of the small molecule, AG10, which binds reversibly to the serum protein transthyretin (TTR)^5^. AG10 was discovered by our group and is currently in Phase III clinical trials for TTR amyloid cardiomyopathy (ATTR-CM)^6,7^. We hypothesize that the high hydrophilicity of AG10 (AG10 exists as a zwitterion at physiological pH) and its binding to TTR in plasma will decrease the passive diffusion of AG10 conjugates across the BBB. In contrast to other approaches, and through binding to TTR, our strategy should be successful in extending the circulation half-life of therapeutic agents without compromising their potency. We evaluated the potential of our approach by developing pharmacological probes that provided important insights on the site of action of opioid drugs (e.g., morphine). This information could broaden our understanding of current clinical issues associated with the undesirable side effects of opioids.

Opioid analgesics are effective in the management of severe acute and chronic pain. However, they are often associated with dose-limiting side effects, such as sedation, nausea, vomiting, constipation, tolerance, and respiratory depression. Opioid-induced constipation (OIC) is the most common side effect of opioid usage affecting 80% of patients who receive opioids for cancer pain or chronic noncancer pain. OIC can be difficult to manage and, in contrast to other side effects, tolerance to OIC does not develop^8–10^. OIC can be severe enough to require opioid discontinuation, exposing the patients to unnecessary pain. Antagonists of mu-opioid receptors, such as naloxone and naltrexone, would be useful to treat OIC. However, these drugs cross the BBB and reverse the intended analgesic effects of opioid agonists and often result in the precipitation of opioid withdrawal syndrome, which can sometimes be life threatening^11,12^. Due to the presence of mu-opioid receptors in the gastrointestinal (GI) tract, it is widely assumed that OIC results primarily from the interaction of opioids with the mu-opioid receptors in the gut. Therefore, a main objective in pain management has been to develop opioid antagonists that do not cross the BBB. There are several peripherally acting mu-opioid receptor antagonists (PAMORAs) that are clinically used to treat OIC. These drugs are designed to have high polarity, which limits their ability to cross the BBB, and therefore, they are not supposed to affect the analgesic effects of opioids in the CNS. Many PAMORAs are based on the structure of morphinan where the additional polarity is introduced by forming a quaternary salt at the morphinan nitrogen (e.g., methylnaltrexone, which is a quaternary derivative of naltrexone). However, the introduction of the quaternary ammonium group in opioid antagonists affects the metabolic stability and often leads to a 100-fold reduction in potency. For example, methylnaltrexone (MNTX) exhibits only 1% of the affinity for the mu-opioid receptors as naltrexone itself^13^. Another clinically used PAMORA is naloxegol, which is a derivative of naloxone in which the tertiary amino group is maintained, and a short polyethylene glycol (PEG) is introduced at position 6 of the morphinan ring^14^.

It is important to note that opioid withdrawal symptoms and an increase in pain have been reported in patients after taking MNTX or naloxegol^15,16^, suggesting that these molecules or their metabolites can cross the BBB. This observation is supported by our data in rats (discussed below) which show that peripheral selectivity is only maintained at low doses of these PAMORAs that often do not produce the maximum desired reversal of OIC. While current PAMORAs have been successful in reversing OIC for a certain number of patients, none of the previous clinical studies have established an improvement in quality of life in patients taking PAMORAs^8^. Unfortunately, the reason for this significant deficiency with current PAMORAs has not been addressed yet. We believe that part of this problem is due to the poor understanding of the role of the CNS vs. GI tract in causing constipation, which resulted from using current PAMORAs as pharmacological probes. We show here that current PAMORAs are poor pharmacological probes that are not suitable for performing accurate in vivo experiments. Therefore, developing a true peripherally selective PAMORAs that do not compromise analgesia or induce withdrawal symptoms at high doses of opioid agonists is highly needed.

**Fig. 1:**
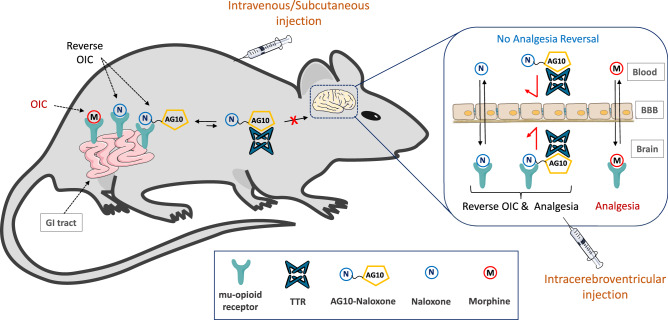
Concept of the AG10-based approach to limit BBB penetration of therapeutic molecules. Lipophilic mu-opioid receptor antagonists (e.g., naloxone) are hypothesized to reverse opioid-induced constipation (OIC) caused by the interaction of morphine (opioid agonist) with mu-opioid receptors in the gastrointestinal (GI) tract. However, these antagonists can also cross the blood-brain barrier (BBB) and displace morphine from the mu-opioid receptors in the brain, which results in reversing the intended analgesic effect of morphine. By conjugating naloxone to the hydrophilic derivative of AG10, we generated an AG10-Naloxone conjugate that has limited penetration across the BBB. The high peripheral selectivity of the AG10-Naloxone conjugate to the mu-opioid receptors in the GI tract is attributed to the high polarity of the AG10 ligand and reversible binding to the abundant plasma protein, transthyretin (TTR). Increasing the hydrophilicity of molecules is typically associated with fast renal excretion and shorter in vivo half-life. Unlike other approaches, the binding of our hydrophilic AG10 conjugates to TTR in plasma (TTR half-life is ~2 days) results in an extended circulation half-life. Because of their high selectivity to the peripheral tissues, the AG10-Naloxone conjugate should reverse the action of opioid agonists (e.g., morphine) in the GI tract without compromising the analgesic effect of opioid agonists in the brain or precipitating opioid-related withdrawal symptoms. In addition, direct intracerebroventricular administration of the AG10-Naloxone conjugate in the brain will allow for evaluating the role of mu-opioid receptors in the brain in causing analgesia and constipation, without any effect on mu-opioid receptors in the periphery.

Here we show that by conjugating the opioid antagonist naloxone to a hydrophilic derivative of AG10, we generate the most potent and peripherally restricted PAMORA (a brief description of the concept of our approach is shown in Fig. 1). We report a comprehensive study of our AG10-Naloxone PAMORA that involves chemical synthesis, in vitro binding and stability assays, and pharmacokinetic and pharmacodynamic evaluations in rats. The high peripheral selectivity of our AG10-based PAMORA reveals insights on the important role of the mu-opioid receptors in the CNS in causing constipation. Importantly, our findings show that synergy or additive effect between the mu-opioid receptors in both the CNS and GI tract is essential for understanding OIC and how to treat it effectively. These findings contradict reports suggesting that OIC occurred through a mechanism involving predominantly the mu-opioid receptors located within the GI tract^17–23^. We also support our results by designing and evaluating a peripherally restricted opioid agonist (AG10-Oxycodone conjugate) that confirms the predominant role of the CNS in precipitating OIC. Therefore, the designed AG10-opioid conjugates represent a class of pharmacological probes that will advance our knowledge base on OIC and other side effects. Aside from their immediate use as pharmacological probes, the designed opioid conjugates have potential as therapeutics for OIC and other yet unexplored therapeutic actions related to antagonizing peripherally localized opioid activity^9,24,25^. This would help in developing safer and more effective opioid therapeutics.

## Results

### Description of the AG10-based approach for limiting penetration across the BBB

**Fig. 2:**
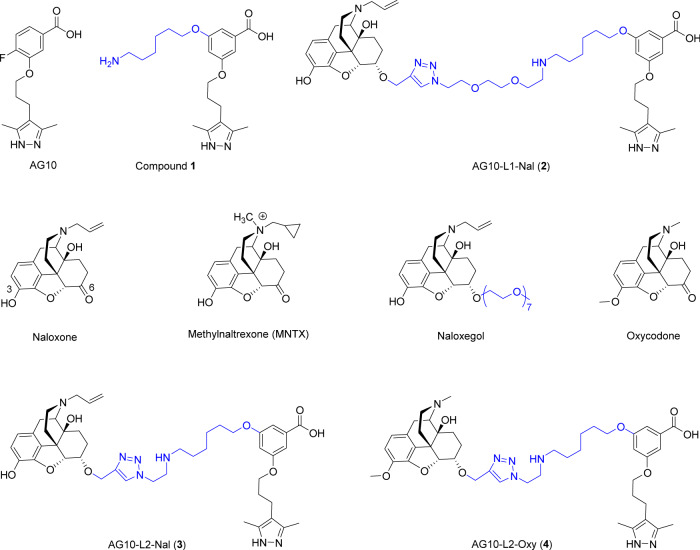
Chemical structures of compounds used in our studies. AG10 and compound **1** are selective TTR ligands. Naloxone is an opioid antagonist that crosses the blood-brain barrier (BBB). MNTX and naloxegol are PAMORAs approved to treat OIC. AG10-L1-Nal and AG10-L2-Nal are conjugates of AG10 and naloxone using linkers (L1 and L2) of different lengths. Oxycodone is an analgesic opioid agonist. AG10-L2-Oxy is a conjugate of AG10 and oxycodone.

TTR is an abundant homo-tetrameric plasma protein (concentration ~5 μM) that is made by the liver. TTR is also synthesized by the choroid plexus and secreted into the cerebrospinal fluid (CSF). Because of its size (55 kDa), the circulation half-life of TTR is ~2 days^26^. TTR transports *holo*-retinol binding protein from the liver to various organs^26^. TTR acts as a backup carrier of thyroxine (<1% thyroxine bound)^27^. The binding sites for thyroxine are orthogonal to those of holo-RBP. We have recently reported a linker-modified hydrophilic derivative of AG10, compound **1** (Fig. 2). AG10 and compound **1** bind to the two thyroxine sites in TTR and do not interfere with the interaction between TTR and holo-RBP^6,28^.

We hypothesized that conjugating the opioid antagonist (naloxone) or agonist (oxycodone) to compound **1** would limit the BBB penetration of these opioids (Fig. 1; illustrating an example with naloxone). The hydrophilicity of **1** would limit the BBB penetration while binding of the AG10 portion of **1** to TTR would increase the half-life of the naloxone and oxycodone conjugates. Importantly, higher affinity of AG10-conjugates to mu-opioid receptors over TTR will allow the conjugate to leave TTR and preferentially bind to mu-opioid receptors. Owing to the reversible binding of these AG10-opioid conjugates to TTR, the half-life of these conjugates will be enhanced, and the intrinsic activity of the drug conjugates would not be adversely affected (Fig. 1). Due to their high polarity, we also hypothesize that direct intracerebroventricular administration of these conjugates in the brain will restrict their permeation out of the CNS to the periphery, extending their CNS action. This should provide these probes with the ability to be concomitantly administered with other opioids to evaluate the precise site of action for their efficacy and side effects.

### Evaluation of the BBB penetration of compound 1 in rats

**Fig. 3:**
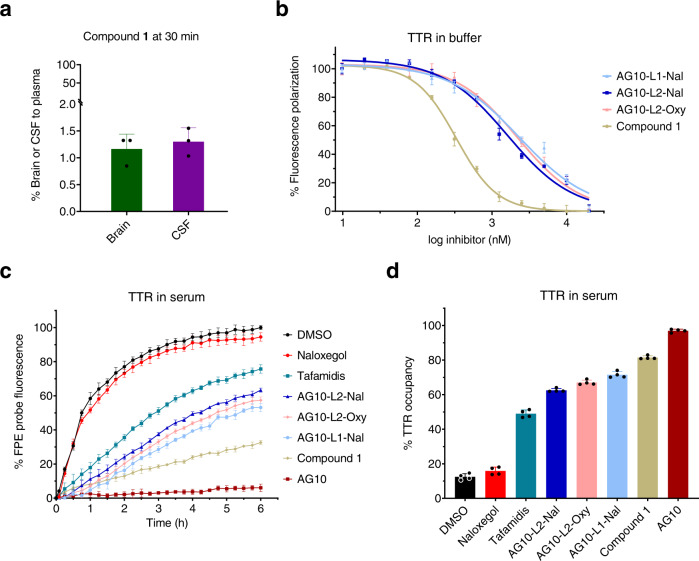
BBB penetration study of compound 1 in rats and binding affinity of 1 and AG10- conjugates (AG10-L1-Nal, AG10-L2-Nal, and AG10-L2-Oxy) to TTR in buffer and human serum. **a** Percentage brain to plasma ratio and percentage CSF to plasma ratio of compound **1** in rats 30 min after intravenous administration of compound **1** (50 µmol/kg; equivalent to 20 mg/kg). The concentration of compound **1** in plasma, brain tissue homogenate, and cerebrospinal fluid (CSF) were determined using validated LC-MS/MS methods. Bar graphs represent the respective mean (±s.d.) (*n* = 3 for each group). **b** Binding affinity of ligands to purified human TTR in buffer was evaluated using fluorescence polarization assay. Compounds were tested at different concentration (0.01 µM to 20 µM). The IC_50_ values were used to calculate the binding constant (*K*_d_) using the Cheng–Prusoff equation. Data represent the mean ± s.d. (*n* = 3). **c** Evaluation of the binding selectivity of ligands (tested at 10 μM) for TTR in human serum (concentration ~5 μM). The modification of TTR in human serum by covalent FPE probe was monitored for 6 h in the presence of FPE probe alone (black circles) or probe and TTR ligands (colors). Ligands that display lower fluorescence of FPE probe have higher binding selectivity to TTR. **d** Percent occupancy of TTR in human serum by ligands in the presence of FPE probe. The 3 h readings, relative to probe alone, was used to calculate the percent occupancy. Error bars indicate mean ± s.d. (*n* = 4). Source data are provided as a Source Data file.

Before we started with opioid antagonists and as a proof of concept, we tested our hypothesis by evaluating the BBB penetration of compound **1** in rats. The plasma concentration of TTR in rats (concentration ∼5 μM) is similar to that of human. Rat TTR has high similarity with human TTR (∼80% sequence homology at the protein levels)^29,30^. While there are some differences between rat and human TTR in the peripheral loop regions, the amino acids in the two thyroxine binding sites, where **1** binds, are conserved between rat and human. Therefore, we do not expect appreciable differences in the binding of **1** and conjugates between human or rat TTR. Compound **1** was administered intravenously (IV) through a cannula in the jugular vein. The concentrations of **1** in plasma, brain tissue, and cerebrospinal fluid (CSF) were quantified using validated LC-MS/MS methods (Supplementary Fig. 1 and Supplementary Table 1; calibration curves). The plasma concentration of **1** after 30 min of the IV bolus injection was 1.85 ± 0.34 µM. The percentage brain to plasma ratio for **1** (1.2%) and the CSF to plasma ratio (1.3%) were less than 2%, which clearly shows that **1** does not have significant penetration across the BBB (Fig. 3a). It is well established that molecules having <2% of its plasma concentration in the brain or CSF are not considered to cross the BBB^31^. These data support our hypothesis that the hydrophilicity and selective binding to TTR limit the ability of the AG10-conjugates to cross the BBB.

### Design, synthesis, and in vitro evaluation of peripherally restricted AG10-Naloxone conjugates

We investigated the potential of our approach in addressing clinical issues associated with the undesirable side effects of opioid agonists such as morphine. We thought that naloxone (Fig. 2) would be an ideal candidate for our approach for multiple reasons. First, naloxone binds with high affinity to the mu-opioid receptor (*K*_i_ = 0.29 nM), which is the main opioid receptor involved with OIC. Second, the lipophilicity of naloxone allows it to rapidly cross the BBB into the brain and achieve a greater brain-to-plasma concentration ratio. This should allow us to evaluate the effectiveness of our approach in limiting the BBB penetration of lipophilic molecules like naloxone. Third, the structure-activity relationship (SAR) of naloxone is well established. It has been shown that modifying position 6 of the morphinan structure does not significantly affect the binding affinity of both agonists and antagonists such as morphine and naloxone, respectively. For example, naloxegol is a derivative of naloxone where a hydrophilic polyethylene glycol side chain has been added at position 6 of naloxone (Fig. 2). Despite this modification, naloxegol maintained high antagonistic potency to the mu-opioid receptors. The well-studied SAR of naloxone strongly indicates that the binding affinity of our designed AG10-Naloxone conjugates to the mu-opioid receptors could be maintained. For synthesis of the AG10-Naloxone conjugates, we first conjugated an azide-modified **1** (i.e., AG10-Linker 1 or AG10-L1) to a naloxone derivative equipped with an alkyne group at position 6 to give AG10-Linker 1-Naloxone (abbreviated as AG10-L1-Nal) (Fig. 2). AG10-L1-Nal and all other compounds tested were >95% purity (full description of the synthesis for all conjugates and HPLC purity analysis can be found in the Supplementary Information).

The binding affinity of AG10-L1-Nal to TTR in buffer was evaluated using fluorescence polarization (FP) binding assay^32^ (*K*_d_ = 485.3 nM; Fig. 3b). This binding affinity is lower than the binding affinity of **1** (*K*_d_ = 68.5 nM; Fig. 3b). However, this decrease in TTR binding affinity might be useful for allowing AG10-L1-Nal to preferably leave TTR and interact with target mu-opioid receptors. For our approach to be successful in vivo, AG10-Naloxone conjugates should be able to selectively bind to TTR in the presence of more than 4,000 other human serum proteins. We evaluated the selectivity of AG10-L1-Nal binding to TTR in human serum using a well-established TTR serum fluorescent probe exclusion (FPE) selectivity assay^33,34^. Our data showed that AG10-L1-Nal maintained very good binding selectivity to TTR in human serum (71.5 ± 1.8% TTR occupancy) (Fig. 3c, d). Importantly, the performance of AG10-L1-Nal was better than that of the TTR stabilizer, tafamidis (an approved drug for TTR amyloidosis; 48.9 ± 2.2% TTR occupancy)^35^.

**Fig. 4:**
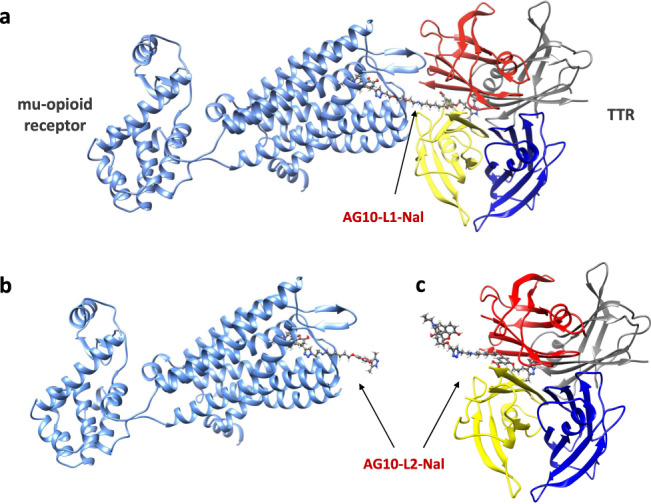
Modeled complexes of AG10-L1-Nal and AG10-L2-Nal to both TTR and mu-opioid receptors. **a** The linker length for AG10-L1-Nal (~20 Å) is long enough to allow AG10-L1-Nal to bind to both the mu-opioid receptor and transthyretin (TTR) simultaneously. The AG10-L2-Nal linker is shorter which allows AG10-L2-Nal to bind to either **b** the mu-opioid receptor (pdb id: 4DKL)^55^ or **c** TTR (pdb id: 4HIQ)^6^ but not both proteins at the same time. Because of the higher affinity of AG10-L2-Nal to the mu-opioid receptor (*K*_i_ = 1.3 nM) over TTR (*K*_d_ = 333 nM), AG10-L2-Nal will preferentially bind to the mu-opioid receptor over TTR.

We then evaluated the in vitro binding affinity of our test compounds to the mu-opioid receptors (Supplementary Fig. 2 and Supplementary Table 2). The binding assay is a competitive radioligand binding assay that measures the potency of test compounds in displacing a radiolabeled DAMGO (a synthetic opioid peptide with high mu-opioid receptor specificity) from recombinant human mu-opioid receptors. The binding affinity of AG10-L1-Nal (*K*_i_ = 0.81 nM) was 3-fold lower than naloxone (*K*_i_ = 0.29 nM) and 3.5-fold higher than naloxegol (*K*_i_ = 2.9 nM) (Supplementary Fig. 2). To mimic the conditions in vivo, we tested the binding affinity of AG10-L1-Nal to the mu-opioid receptors in the presence of excess TTR (1 µM). Interestingly, there was a 9-fold decrease in the binding affinity of AG10-L1-Nal to the mu-opioid receptors when TTR was present (*K*_i_ = 7.4 nM). To investigate the reason behind this decrease in binding potency, we performed a modeling study where we evaluated the binding of AG10-L1-Nal to both TTR and the mu-opioid receptor. It was clear that the length of the linker between AG10 and naloxone (~20 Å) is long enough to allow the formation of a ternary complex with both TTR and mu-opioid receptor (Fig. 4a). We hypothesize that this ternary complex likely resulted in some steric hindrance from TTR that affected the binding affinity of AG10-L1-Nal to the mu-opioid receptor.

To address this issue, we designed a conjugate (AG10-L2-Nal; Fig. 2) in which the linker length was reduced to ~12 Å. The binding affinity of AG10-L2-Nal to TTR in buffer (*K*_d_ = 333 nM; Fig. 3b) and binding selectivity to TTR in human serum (62.5 ± 1.1%TTR occupancy) (Fig. 3c, d) were similar to that of AG10-L1-Nal. The shorter linker of AG10-L2-Nal was sufficient to allow it to bind to either TTR or the mu-opioid receptor but not to both at the same time (Fig. 4b, c). To test our hypothesis, we determined the binding affinity of AG10-L2-Nal to the mu-opioid receptors in the absence and presence of TTR. Interestingly, the binding affinity of AG10-L2-Nal (*K*_i_ = 0.35 nM) was comparable to that of naloxone (*K*_i_ = 0.29 nM) and 8.3-fold higher than that of naloxegol (*K*_i_ = 2.9 nM). In the presence of excess TTR (1 µM), the binding affinity of AG10-L2-Nal (*K*_i_ = 1.3 nM) was 5.7-fold and 2.2-fold higher than what we observed for AG10-L1-Nal and naloxegol, respectively (Supplementary Fig. 2). The binding affinity of AG10-L2-Nal to the mu-opioid receptor in the presence of TTR was also 17-fold better than MNTX (*K*_i_ = 22.1 nM)^14^. Therefore, it was clear that the linker system plays an important role in the preferential binding to the mu-opioid receptor over TTR. Because of its improved binding affinity, we decided to proceed with the biological evaluation of AG10-L2-Nal as our lead molecule.

### AG10-L2-Nal displayed competitive antagonistic activity in mu-opioid receptor functional assays

In the [^35^S]GTPγS functional binding assay, using membranes of CHO-K1 cells stably expressing human mu-opioid receptors, AG10-L2-Nal (20 µM) exhibited 2% agonism (mean value of 4 experiments) relative to DAMGO (EC_50_ = 13.2 nM). Naloxegol displayed 6% agonism at 20 µM. Parallel testing of AG10-L2-Nal, naloxegol, and the potent opioid antagonist naltrexone in the mu-opioid receptor functional assay demonstrated that AG10-L2-Nal potency (IC_50_ = 7.5 nM) is similar to that of naltrexone (IC_50_ = 10.9 nM) and 10-fold more potent than naloxegol (IC_50_ = 72 nM) (Supplementary Fig. 3). These values correspond to equilibrium dissociation constant (*K*_B_) of 0.81 nM for AG10-L2-Nal and 7.7 nM for naloxegol. The *K*_B_ value for naloxegol is similar to the reported literature value (*K*_B_ = 11 nM)^14^. Importantly, our functional assay data fit well with the competitive binding assay data, discussed above, where the binding affinity of AG10-L2-Nal (*K*_i_ = 0.35 nM) is 8-fold higher than that of naloxegol (*K*_i_ = 2.9 nM).

In Schild-type experiments, AG10-L2-Nal elicited parallel rightward shifts in the morphine dose-response curve without any accompanying reduction in the maximal response (E_max_) produced by morphine (Supplementary Fig. 4). The EC_50_ of morphine (EC_50_ = 0.039 µM) was increased by 950-fold in the presence of AG10-L2-Nal (the EC_50_ of morphine + 0.4 µM AG10-L2-Nal = 37.2 µM). These results demonstrate that AG10-L2-Nal is a potent competitive antagonist of morphine at the human mu-opioid receptor.

### AG10-L2-Nal displayed potent activity towards other opioid receptors

To ensure that modifying the structure of naloxone with AG10-linker does not significantly affect the binding to other opioid receptors, we evaluated the binding of AG10-L2-Nal to the delta- and kappa-opioid receptors. In comparison to both naloxegol and MNTX, AG10-L2-Nal maintained excellent binding affinity to both delta- and kappa-opioid receptors (Supplementary Figs. 5 and 6). Similar to naloxone (*K*_i_ = 12.6 nM and 2 nM for the delta- and kappa-opioid receptor, respectively), AG10-L2-Nal binds with high affinity to the delta- and kappa-opioid receptors (*K*_i_ = 5.5 nM and 0.12 nM, respectively). This is significantly higher than naloxegol (*K*_i_ = 203 nM and 8.7 nM for the delta- and kappa-opioid receptors, respectively) and MNTX (*K*_i_ = 1,900 nM and 10.9 nM for delta- and kappa-opioid receptors, respectively)^14^.

### AG10-L2-Nal does not cross the BBB in rats

**Fig. 5:**
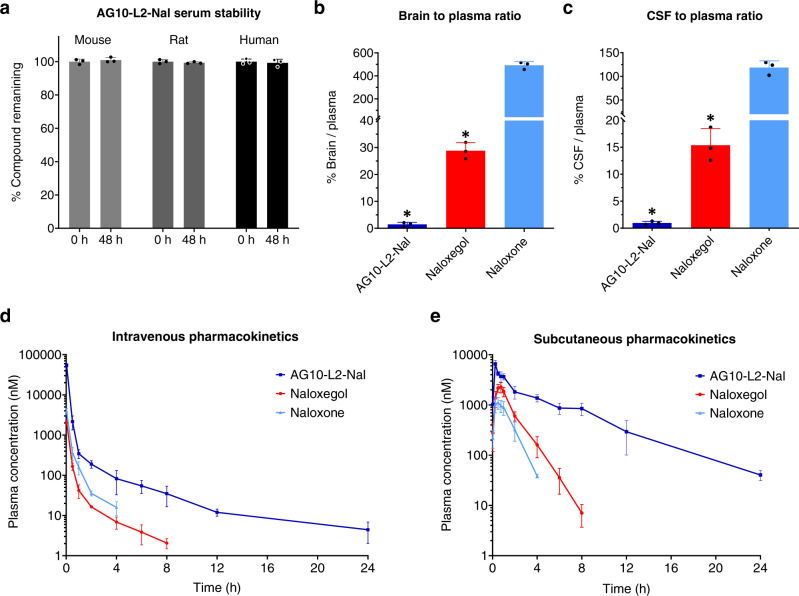
Evaluation of serum stability, BBB penetration, and intravenous/subcutaneous pharmacokinetics of AG10-L2-Nal, naloxone, and naloxegol. **a** Serum stability of AG10-L2-Nal was evaluated in mouse, rat, and human sera. AG10-L2-Nal (100 μM) was incubated in sera, and the concentration remaining in respective media was determined at 0 and 48 h using HPLC. Bar graphs represent the mean of % compound remaining ± s.d. (*n* = 3). **b** Brain to plasma ratios and **c** cerebrospinal fluid (CSF) to plasma ratios of AG10-L2-Nal, naloxegol, and naloxone. Male Sprague Dawley rats were dosed intravenously with 4.84 µmol/kg of test compounds (equivalent to 1.6 mg/kg, 3.2 mg/kg, and 4 mg/kg for naloxone, naloxegol, and AG10-L2-Nal, respectively). The plasma, brain tissue, and CSF were collected at 30 min after dosing. The ratio of the brain (ng/g) versus plasma concentration (ng/mL) is expressed as the percentage brain to plasma ratio. The ratio of the CSF (ng/mL) versus plasma concentration (ng/mL) is expressed as the percentage CSF to plasma ratio. Bar graphs show the respective mean (±s.d.) (*n* = 3 for each group). Statistical differences were determined using one-way ANOVA followed by Tukey’s Post Hoc test (*P < 0.05 compared to naloxone). For the brain to plasma ratio experiment F(2,6) = 678.0, *P* < 0.0001 and for the CSF to plasma ratio experiment F(2,6) = 174.6, *P* < 0.0001. **d** a single intravenous bolus dose of 4.84 µmol/kg and **e** a single subcutaneous injection of 16 µmol/kg dose of antagonists (equivalent to 5.2 mg/kg, 10.4 mg/kg, and 13.2 mg/kg for naloxone, naloxegol, and AG10-L2-Nal, respectively) were administered to rats (*n* = 3 for each group). The concentration of the test compounds in plasma was determined at different time points and expressed as means ± s.d. of three biological replicates. Source data are provided as a Source Data file.

Before performing our in vivo BBB penetration and pharmacokinetic studies, we assessed the stability of AG10-L2-Nal in mouse, rat, and human sera (Fig. 5a and Supplementary Fig. 7). HPLC analysis showed that AG10-L2-Nal was stable in all three sera (100% remaining) for at least 48 h when incubated at 37 °C. For the BBB penetration study, we compared AG10-L2-Nal to the two FDA-approved opioid antagonists, naloxone (opioid antidote that readily crosses the BBB) and naloxegol (a PAMORA approved for OIC due to its limited BBB) (Fig. 5b, c). Test compounds were intravenously administered to rats via a jugular vein cannula. The concentration of test compounds in plasma, brain tissue, and CSF 30 min after dosing was quantitated using LC-MS/MS (Supplementary Fig. 8 and Supplementary Table 1; calibration curves). The percentage brain to plasma ratio and CSF to plasma ratios of naloxone were ~490% and 119%, respectively. These data are consistent with the literature which showed that the concentration of naloxone is higher in the brain and CSF than in plasma^12^. The brain to plasma ratio and CSF to plasma ratio for naloxegol were 29% and 15%, respectively. The 15-29% of naloxegol that crosses the BBB is much higher than the 2% threshold for classifying the compound’s ability to cross the BBB. While the brain and the CSF levels of naloxegol were markedly reduced compared to naloxone, it was surprising that significant levels of naloxegol crossed the BBB. In contrast, the brain to plasma ratio and CSF to plasma ratio of AG10-L2-Nal (1.4% and 1%, respectively) were significantly lower than naloxone. For AG10-L2-Nal, there was a 20-fold decrease in brain to plasma ratio and a 15-fold decrease in CSF to plasma ratio compared to naloxegol. This result clearly demonstrates that AG10-L2-Nal is a highly peripherally restricted opioid antagonist.

### TTR extended the circulation half-life of AG10-L2-Nal in rats

We have evaluated the pharmacokinetic properties of AG10-L2-Nal, naloxone, and naloxegol in rats. These molecules were first administered as single intravenous injections (4.84 µmol/kg) (Fig. 5d and Supplementary Table 3). Blood samples were withdrawn from the jugular vein cannula at predetermined time points (ranging from 2 min to 24 h), and concentrations of the test compounds were quantitated using validated LC − MS/MS methods (Supplementary Fig. 8 and Supplementary Table 1). The pharmacokinetic profile of AG10-L2-Nal was markedly different from naloxone and naloxegol. The plasma concentrations of AG10-L2-Nal were higher than naloxone and naloxegol at any given time. While there was no measurable amount of naloxone and naloxegol 4 and 8 h after dosing, respectively, AG10-L2-Nal was still present even after 24 h. There was a 7-fold and 3.5-fold increase in the half-life of AG10-L2-Nal (half-life = 5.98 ± 0.81 h) compared to naloxone and naloxegol (half-lives = 0.87 ± 0.13 h and 1.72 ± 0.27 h, respectively). This was due to the lower clearance of AG10-L2-Nal (0.29 ± 0.02 L/h/kg), which was significantly lower than naloxone and naloxegol (3.39 ± 0.55 L/h/kg and 6.54 ± 0.29 L/h/kg, respectively). Importantly, the AUC_inf_ (exposure) of AG10-L2-Nal (16912.96 ± 1085.27 nM·h) was ∼23-fold and 12-fold higher than naloxegol and naloxone (741.34 ± 33.65 and 1455.11 ± 257.18 nM.h, respectively). These data strongly support and validate our approach that TTR recruitment can indeed enhance the half-life and pharmacokinetic profile of AG10-L2-Nal in vivo.

For AG10-L2-Nal to have a practical clinical application, we investigated the subcutaneous route of administration in rats. AG10-L2-Nal, naloxone, and naloxegol were administered as single subcutaneous doses (16 µmol/kg) (Fig. 5e, Supplementary Fig. 8, and Supplementary Tables 1, 4). We obtained similar pharmacokinetic profiles for all three compounds as observed in the intravenous route. AG10-L2-Nal was absorbed rapidly 2 min after subcutaneous dosing. As observed in the intravenous administration, AG10-L2-Nal has a more favorable half-life, clearance rate, and AUC_inf_ compared to naloxone and naloxegol (Supplementary Table 4). These data show that AG10-L2-Nal has the potential to be administered using the less invasive and more convenient subcutaneous route.

### AG10-L2-Nal does not reverse morphine-induced analgesia in rats after intravenous administration

Our pharmacokinetic and BBB data show that AG10-L2-Nal has a better pharmacokinetic profile and the lowest BBB penetration compared to naloxone and naloxegol. Before investigating the potency of AG10-L2-Nal in reversing OIC, we wanted to determine if the AG10-L2-Nal data can be translated into less reversal of morphine-induced analgesia. For this purpose, we used the rat hot plate model where the degree of analgesia is evaluated by measuring the length of time that an animal can tolerate heat from a hot plate maintained at 55 °C. We compared the efficacy of AG10-L2-Nal to two FDA-approved PAMORAs used to treat OIC (i.e., MNTX and naloxegol). We also included naloxone as an opioid antagonist with both CNS and peripheral nervous system action.

**Fig. 6:**
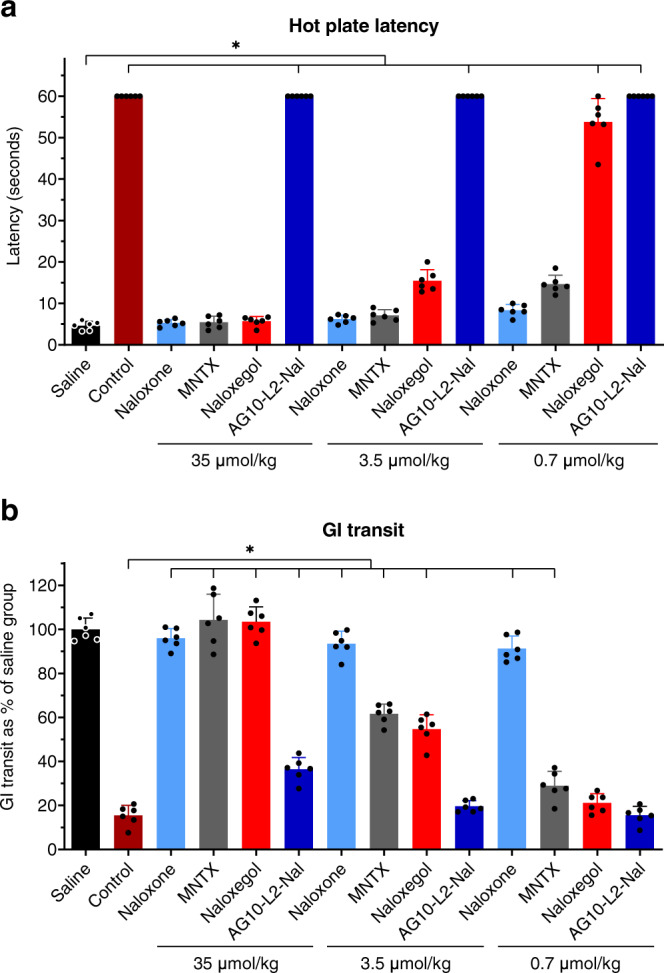
Hot plate and gastrointestinal (GI) transit efficacy studies of opioid antagonists in male Sprague-Dawley rats. **a** Hot plate latency test to measure analgesia. Male Sprague-Dawley rats were first administered with saline or a single intravenous (IV) dose of morphine (35 µmol/kg; equivalent to 10 mg/kg). After 5 min, the morphine treated animals were administered with a single intravenous dose of vehicle or the opioid antagonists. Saline group: saline + vehicle; control group: 35 µmol/kg morphine + vehicle; all other groups: 35 µmol/kg morphine + specified dose of antagonists. The 35 µmol/kg dose of antagonists represent 11.5 mg/kg, 23 mg/kg, 12.5 mg/kg, and 30 mg/kg for naloxone, naloxegol, methylnaltrexone (MNTX), and AG10-L2-Nal, respectively. The hot plate withdrawal latency to heat exposure (withdrawal or shaking of the hind paw, sharp withdrawal, licking of fore or hind paw, or attempting to escape by jumping) was recorded 1 h after the morphine dose before the rats were removed from the hot plate. Statistical differences were determined using Kruskal–Wallis test followed by Dunn’s multiple comparisons test, H = 77.02, *P* < 0.0001. All data are presented as mean (±s.d.) (*n* = 6 rats per group, *P < 0.05). **b** Gastrointestinal (GI) transit assay at 1 h after different IV bolus doses of the test compounds. The dosing schedule is similar to the hot plate assay with an additional oral gavage of charcoal meal 30 min after the saline or morphine dose. The significance of differences was measured by two-way ANOVA followed by Tukey’s post hoc test (*n* = 6 rats per group, *P < 0.05), dose F(2,90) = 262.3, *P* < 0.0001, compound F(5,90) = 670.8, *P* < 0.0001. Source data are provided as a Source Data file.

Rats were first dosed with saline or a single dose of morphine, and 5 min later, the animals were given a single dose of the opioid antagonists. In comparison to rats injected with saline (latency = 4.6 ± 1.0 seconds), rats injected with morphine (a single dose of 10 mg/kg or 35 µmol/kg) were insensitive to heat 1 h after dosing (latency at 1 h after morphine dose = 60 seconds, which is the maximum cutoff time to remove the animals from the hot plate) (Fig. 6a). The morphine data is consistent with the literature where a dose of 35 µmol/kg resulted in full analgesia. We then tested the antagonists at doses equivalent to concomitantly administered morphine (single doses of 35 µmol/kg). As expected, naloxone was very effective in reversing morphine-induced analgesia (latency = 5.3 ± 0.8 seconds). Surprisingly, both MNTX and naloxegol were also effective in fully reversing morphine-induced analgesia (latency = 5.4 ± 1.5 seconds and 5.7 ± 1.2 seconds, respectively) (Fig. 6a). In contrast, at 35 µmol/kg, the AG10-L2-Nal group maintained full morphine-induced analgesia with a latency of 60 seconds. It is important to note that the AG10-L2-Nal dose is ~7-fold higher than the dose we used in the BBB study (Fig. 5b, c), which shows that our strategy is very effective in limiting the BBB penetration of the naloxone conjugate. To evaluate the contribution of TTR in limiting the diffusion of AG10-L2-Nal across the BBB, we repeated the analgesia experiment in the presence of the potent TTR ligand, AG10. AG10 binds to TTR with a higher affinity than AG10-L2-Nal (*K*_d_ = 5 nM and 333 nM, respectively) and therefore, the presence of saturating concentration of AG10 (dosed at 50 mg/kg) will diminish the effect of TTR binding on the pharmacokinetic properties of AG10-L2-Nal. There was no reversal of analgesia by AG10-L2-Nal (35 µmol/kg, equivalent to 30 mg/kg) in the presence of AG10 (Supplementary Fig. 9a). The plasma concentration of AG10-L2-Nal (7.6 ± 0.5 µM) was higher than the plasma concentration of TTR (5 µM), which in addition to the presence of AG10 in our experiment, indicate that the high polarity of our conjugates (due to the presence of the zwitterion in AG10) is the main driving force for limiting the passage of the conjugates across the BBB. These data are also consistent with data for compound **1** where even at a higher dose (50 µmol/kg; Fig. 3a), there was no significant BBB penetration.

Next, we tested the ability of antagonists to reverse morphine-induced analgesia at a 10-fold lower dose (3.5 µmol/kg) than morphine. Naloxone and MNTX were effective in completely reversing analgesia (latency = 6.2 ± 0.9 seconds and 7.1 ± 1.4 seconds, respectively) whereas naloxegol resulted in a significant decrease in latency (latency = 15.5 ± 2.6 seconds) (Fig. 6a). These data agree well with our BBB data (Fig. 5b, c), which show that a ~30% of the naloxegol in plasma crosses the BBB at a comparable dose of 4.8 µmol/kg. Subsequently, we tested the antagonists at a 50-fold lower dose (0.7 µmol/kg) than morphine. Naloxone resulted in significant reversal of analgesia (latency = 8.3 ± 1.4 seconds). At this lower dose, there was partial reversal of morphine-induced analgesia for MNTX (latency = 14.7 ± 2.2 seconds) and no reversal of analgesia for naloxegol (latency = 53.8 ± 5.6 seconds) (Fig. 6a). The significant reversal of analgesia by MNTX has implications for patients with advanced illnesses (e.g., incurable cancer, end-stage COPD/emphysema, cardiovascular disease, HIV/AIDS, and Alzheimer’s disease/dementia)^15^ where the doses of opioid agonists needed are very high^36^ and the loss of analgesia during the administration of PAMORAs is not an option.

### Evaluation of the reversal of OIC in rats after intravenous administration of AG10-L2-Nal and antagonists

The GI transit model was employed to determine the efficacy of opioid antagonists in reversing OIC, caused by morphine, by measuring the distance traveled by an orally administered charcoal within the GI tract of rats. Single doses of antagonists were administered 5 min after morphine dose (a single intravenous dose of 35 µmol/kg). In the absence of antagonists, morphine reduced GI transit of a charcoal meal to ~15.5% of drug-free control (Fig. 6b). Naloxone was very effective in fully restoring GI transit and reversing OIC at all the doses tested (~96% charcoal GI transit relative to saline group animals). At 35 µmol/kg (doses equivalent to morphine), MNTX effectively restored full GI transit to ~100%. Reducing the doses of MNTX by 10-fold (3.5 µmol/kg) also resulted in significant reversal of OIC (GI transit = 61.6 ± 4.4%). When tested at 0.7 µmol/kg (the only dose that showed partial hot plate analgesia, Fig. 6a), MNTX displayed reduced charcoal movement (GI transit = 29.0 ± 6.5%) compared to the higher doses (Fig. 6b). While naloxegol was effective in reversing OIC at doses of 35 µmol/kg and 3.5 µmol/kg (GI transit = 100% and 54.6 ± 6.5%, respectively), there was no significant reversal of OIC for naloxegol at the 0.7 µmol/kg (a dose that resulted in no reversal of morphine-induced analgesia, Fig. 6a, b). Interestingly, the doses of naloxegol that were effective in reversing OIC were also effective in reversing analgesia (Fig. 6a). These doses have been demonstrated to have significant CNS penetration (Fig. 5b, c). It was very surprising to see that AG10-L2-Nal was only partially effective in reversing OIC at the highest dose of 35 µmol/kg (GI transit = 36.4 ± 5.4%). We investigated any potential effect of TTR binding on the efficacy of AG10-L2-Nal. There was no improvement in the OIC in the presence of AG10 (Supplementary Fig. 9b). This is somewhat predicted since the binding affinity of AG10-L2-Nal to the mu-opioid receptor is similar in the absence and presence of TTR (0.35 nM vs 1.3 nM) and the plasma concentration of AG10-L2-Nal in rat efficacy studies is in the micromolar range. The data for the test compounds show that there is a clear direct relationship between the level of reversal of analgesia (with documented CNS site of action for opioids) and reversal of OIC (Fig. 6a, b). Antagonists that have higher BBB penetration (Fig. 5b, c) displayed more efficient reversal of OIC, indicating a major role of the CNS in precipitating OIC (discussed below). Our AG10-L2-Nal data also show that we have developed what we believe is the most peripherally selective PAMORA that does not show any significant BBB penetration, as shown here in rats.

### AG10-L2-Nal does not have partial agonistic activity to the mu-opioid receptor

**Fig. 7:**
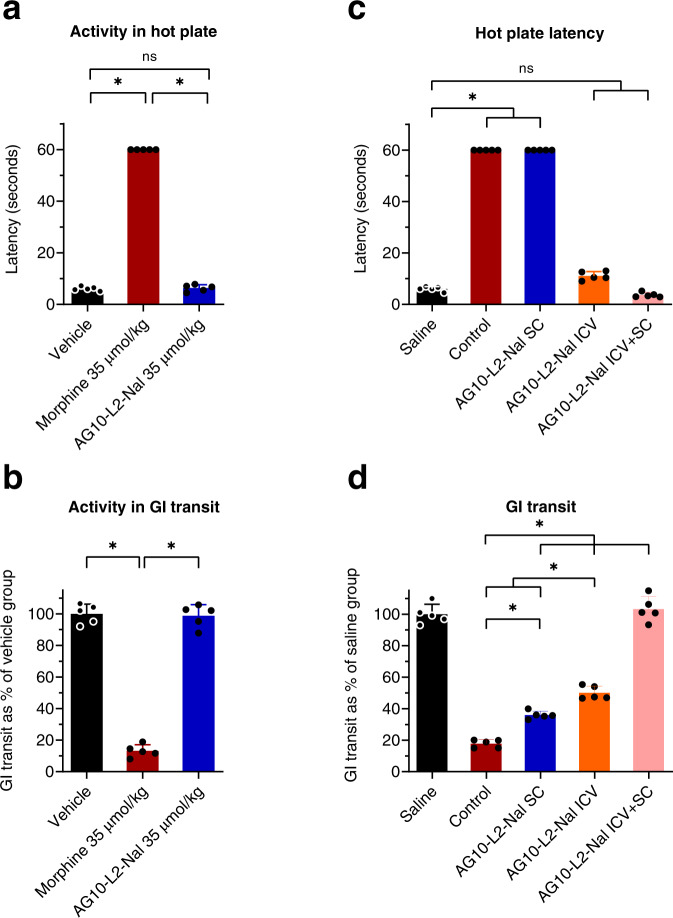
Evaluating the potential partial agonistic behavior of AG10-L2-Nal and contribution of mu-opioid receptors in the CNS to analgesia and OIC. **a** Hot plate latency, statistical differences were determined using Kruskal–Wallis test followed by Dunn’s multiple comparisons test, H = 9.769, *P* = 0.0021 **b** and gastrointestinal (GI) transit assays to evaluate potential partial agonistic behavior of AG10-L2-Nal. Hot plate latency or GI transit were checked 1 h after the subcutaneous dose of vehicle, morphine (35 μmol/kg), and AG10-L2-Nal (35 μmol/kg) in male Sprague-Dawley rats. Statistical differences were determined using one-way ANOVA followed by Tukey’s post hoc test, F(2,12)=354.4, *P* < 0.0001 **c** Hot plate latency, the significance of differences was measured by Kruskal–Wallis test followed by Dunn’s multiple comparisons test, H = 23.23, *P* = 0.0001 and **d** GI transit assays to evaluate the contribution of central and peripheral mu-opioid receptors to OIC. AG10-L2-Nal (35 µmol/kg; subcutaneous route, SC) and/or (0.35 µmol/kg equivalent to 88 nmol per rat; intracerebroventricular route, ICV) or vehicle (control group) was administered at *t* = 0 min. Morphine (35 μmol/kg SC) or saline (saline group) was administered at 10 min. The antagonists were administered 10 min before morphine to allow enough time to handle the animals during the ICV and SC dosing. Charcoal was given at 40 min. Hot plate latency and GI transit were measured at 70 min. Statistical differences were determined using one-way ANOVA followed by Tukey’s post hoc test F(4,20)=274.7, *P* < 0.0001. All data are presented as mean (±s.d.) (*P < 0.05, *n* = 5 rats per group). Source data are provided as a Source Data file.

While AG10-L2-Nal effectively reverses OIC, it was surprising that its efficacy was lower than that of lower doses of naloxone and other PAMORAs. AG10-L2-Nal is more potent on the mu-opioid receptor and, when given at equivalent doses, has higher concentration in the periphery (i.e., higher plasma concentration and AUC_inf_) than naloxegol. Therefore, the effect of AG10-L2-Nal should be higher on reversing OIC. The binding affinities of the antagonists used in our studies were determined for human mu-opioid receptors, while the in vivo efficacy studies were performed in rats. However, the mu-opioid receptors for human and rat share 94% similarity in the amino acid sequence^37^ and therefore, we do not anticipate this to be a main factor in the lower in vivo efficacy of AG10-L2-Nal. To check that the ~40% reversal of GI transit by AG10-L2-Nal was not due to partial agonistic activity, we evaluated AG10-L2-Nal by itself in the hot plate and GI transit tests. The goal of the hot plate analgesia and GI transit assays was to see whether AG10-L2-Nal showed any partial agonistic characteristics due to the structural modification of naloxone. In comparison to morphine, AG10-L2-Nal did not display any analgesic (latency = 6.3 ± 1.3 seconds) or OIC effects (GI transit = 98.7 ± 7.2%). These results confirm our mu-opioid receptor functional assay data (Supplementary Figs. 3 and 4) and demonstrate that AG10-L2-Nal acts as a pure antagonist (Fig. 7a, b).

### AG10-L2-Nal provides insights on the critical role of mu-opioid receptors in the CNS in causing OIC

The role of CNS in precipitating OIC is not well defined. The majority of studies suggest that opioid agonists reduce GI motility through a mechanism involving predominantly the mu-opioid receptors in the GI tract, and this indeed is the thesis behind the development of current PAMORAs. Our efficacy data in rats (Fig. 6) indicated that the CNS plays a major role in precipitating OIC. However, it was not clear from our data if the mu-opioid receptors in the CNS are directly involved in OIC. To investigate this, we performed an experiment in which rats were administered with subcutaneous morphine (will be distributed in the periphery and CNS) 10 min after administering AG10-L2-Nal either directly in the brain (i.e., intracerebroventricular injection, ICV), subcutaneously (SC), or both ICV + SC. Due to the high polarity of AG10-L2-Nal, ICV administration should restrict the effect of AG10-L2-Nal to the CNS, SC administration will only target the periphery, and ICV + SC administration will target mu-opioid receptors in both CNS and periphery. Our SC data were consistent with the data observed for IV administration; there was no reversal of analgesia (latency = 60 seconds; Fig. 7c) and partial reversal of OIC (GI transit = 36.0 ± 2.5% compared to 17.8 ± 2.6% for morphine) (Fig. 7d). ICV administration of AG10-L2-Nal resulted in effective reversal of analgesia (latency = 11.1 ± 1.7 seconds) and 50.1 ± 4.2% reversal of OIC, which is higher than what we observed for the SC route. While the ICV effect of AG10-L2-Nal on reversing analgesia is predicted (based on the established role of mu-opioid receptors in the CNS in causing analgesia), the OIC results were somewhat surprising. Interestingly, concomitant administration of AG10-L2-Nal via both ICV + SC routes resulted in full reversal of both analgesia (latency = 3.6 ± 0.9 seconds) and OIC (GI transit = 100%) (Fig. 7c, d). These data strongly suggest the presence of a synergistic or additive effect in targeting mu-opioid receptors in both periphery and CNS to induce full effect on both analgesia and OIC. While synergy was reported for the analgesic effect of opioids^38^, our data also suggest the presence of synergy or additive effect for OIC.

We next investigated if the results obtained with morphine are applicable to other more potent mu-opioid receptor agonists. We evaluated the ability of AG10-L2-Nal and naloxegol to reverse the analgesia and OIC induced by fentanyl^39^. AG10-L2-Nal (tested at 35 µmol/kg, equivalent to 30 mg/kg) displayed similar data to what we observed with morphine (i.e., no reversal of analgesia and minimal reversal of OIC) (Supplementary Fig. 10). AG10-L2-Nal displayed similar data even when tested at 100 mg/kg. The data obtained for naloxegol were also consistent with the morphine experiment. Naloxegol was effective in reversing both analgesia and OIC induced by fentanyl (Supplementary Fig. 10).

### Development of a peripherally restricted opioid agonist probe

To confirm our results above, we next investigated the role of CNS vs. periphery in inducing analgesia and OIC using AG10-Oxycodone conjugate as an agonist probe (AG10-L2-Oxy, Fig. 2). This probe should provide direct agonistic activity without the need for concomitant administration of morphine as we did for the AG10-L2-Nal experiment. We decided to choose oxycodone as the opioid agonist for our probe for two reasons. First, we wanted to use an opioid other than morphine, which should allow us to determine if the data we observed with morphine can be applied to other opioid agonists. Second, oxycodone has a very high brain-to-plasma ratio (~3-fold) compared to morphine^40,41^, and therefore, it would allow us to evaluate the applicability of our approach to limit the BBB penetration of molecules other than naloxone. AG10-L2-Oxy demonstrated potent and selective binding to TTR in buffer and human serum (Fig. 3b–d). In addition, AG10-L2-Oxy demonstrated potent and selective binding to the mu-opioid receptor (*K*_i_ = 13 nM vs. 3.1 nM for oxycodone) compared to the delta-opioid receptor (*K*_i_ = 410 nM vs. 958 nM for oxycodone) and kappa-opioid receptor (*K*_i_ = 120 nM vs. 677 nM for oxycodone)^42^.

**Fig. 8:**
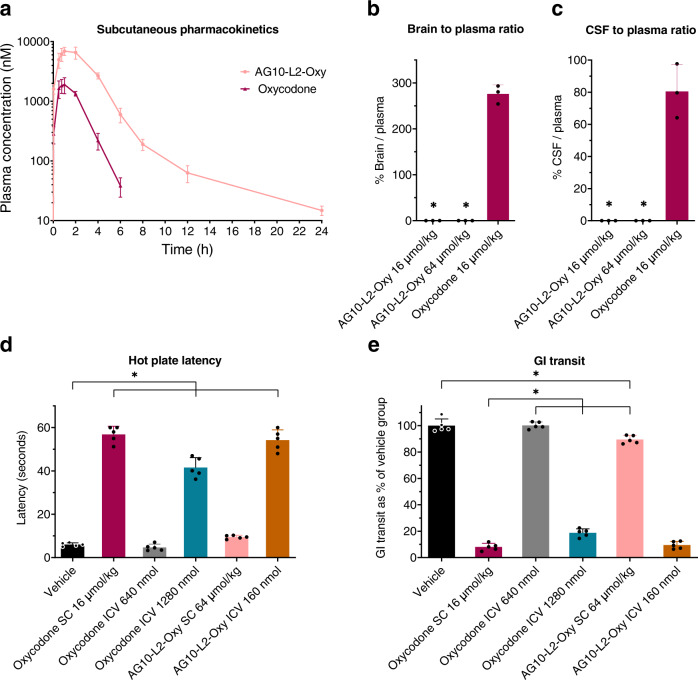
Pharmacokinetic and pharmacodynamic evaluation of AG10-Oxycodone conjugate (AG10-L2-Oxy). **a** Pharmacokinetic profile of AG10-L2-Oxy and oxycodone after a single subcutaneous dose of 16 µmol/kg to rats (*n* = 4 per group). Plasma concentration of compounds is expressed as means ± s.d. of three biological replicates. **b** Brain to plasma ratios and **c** cerebrospinal fluid (CSF) to plasma ratios of AG10-L2-Oxy and oxycodone (*n* = 3 per group). Male rats were dosed subcutaneously with 16 µmol/kg (equivalent to 5 mg/kg) oxycodone and both 16 and 64 µmol/kg AG10-L2-Oxy (equivalent to 13 mg/kg and 52 mg/kg, respectively). The plasma, brain tissue, and CSF were collected at 60 min after dosing. The ratio of the brain (ng/g) versus plasma concentration (ng/mL) is expressed as the percentage brain to plasma ratio. The ratio of the CSF (ng/mL) versus plasma concentration (ng/mL) is expressed as the percentage CSF to plasma ratio. Bar graphs show the respective mean (±s.d.) (n = 3 per group). Statistical differences were determined using One Way ANOVA followed by Tukey’s post-hoc test (*P < 0.05 compared to oxycodone 16 µmol/kg group). For the brain to plasma ratio experiment F(2,6) = 577.6, *P* < 0.0001 and for the CSF to plasma ratio experiment F(2,6) = 68.84, *P* < 0.0001. **d** Hot plate latency and **e** GI transit assays to evaluate the contribution of opioid agonists on central and peripheral mu-opioid receptors in OIC. Oxycodone (16 µmol/kg; subcutaneous route, SC), Oxycodone (640 and 1280 nmol per rat; intracerebroventricular route, ICV), AG10-L2-Oxy (64 µmol/kg; subcutaneous route, SC), AG10-L2-Oxy (160 nmol per rat; intracerebroventricular route, ICV), or vehicle was administered at t = 0 min. Charcoal was given at 10 min. Hot plate latency and GI transit were measured at 40 min. Bar graph showing the respective mean (±s.d.) (*n* = 5 rats per group). Statistical differences were determined using one-way ANOVA followed by Tukey’s post-hoc test (*P < 0.05). F(5,24) = 300.5, *P* < 0.0001 for the hot plate assay and F(5,24) = 983.3, *P* < 0.0001 for the GI transit assay. Source data are provided as a Source Data file.

We then evaluated the pharmacokinetic properties of AG10-L2-Oxy and oxycodone after a single subcutaneous injection in rats (Fig. 8a). The pharmacokinetic profile of AG10-L2-Oxy was markedly different from oxycodone. The concentrations of AG10-L2-Oxy in plasma were higher than oxycodone at any given time. While there was no measurable amount of oxycodone 6 h after dosing, AG10-L2-Oxy was still present even after 24 h (detailed pharmacokinetic parameters are shown in Supplementary Fig. 11 and Supplementary Table 1 and 5). We then evaluated the BBB penetration of AG10-L2-Oxy in rats (Fig. 8b, c). The percentage brain to plasma ratio and CSF to plasma ratios of oxycodone were 276% and 80%, respectively, which is consistent with the literature data for oxycodone^41,43^. In contrast, there was no detectable amounts of AG10-L2-Oxy in either brain or CSF, even at a dose 4-fold higher than that of oxycodone (Fig. 8b, c).

### AG10-L2-Oxy confirms the role of CNS in precipitating analgesia and OIC

We performed an experiment in which rats were administered with subcutaneous or intracerebroventricular injections of oxycodone or AG10-L2-Oxy. As expected, subcutaneous administration of oxycodone (will be distributed in the periphery and CNS) resulted in full analgesia (latency = 56.8 ± 3.8 seconds compared to 6.0 ± 0.8 seconds for vehicle) and inhibition of GI transit (8.0 ± 2.7% compared to 100 ± 5.1% for vehicle) (Fig. 8d, e). Subcutaneous administration of AG10-L2-Oxy resulted in a minor effect on both analgesia (latency = 9.5 ± 0.8 seconds) and GI transit (89.5 ± 3.0%). The ~10% OIC caused by the peripheral action of AG10-L2-Oxy (Fig. 8e) is comparable to ~18% the reversal of morphine-induced constipation we observed for AG10-L2-Nal (Fig. 7d). Intracerebroventricular administration of oxycodone at a dose 4-fold higher than that of AG10-L2-Oxy did not result in significant analgesia or constipation. However, there was a significant analgesic effect (41.6 ± 4.6 seconds) and inhibition of GI transit (18.7 ± 3.0%) at a dose 8-fold higher than that of AG10-L2-Oxy. This could be a result of the higher clearance rate of oxycodone out of the brain (15-fold faster than morphine)^41,44^ and fits with reports that less than 2% of oxycodone remains in the brain 10 min after intracerebroventricular administration^45^. In contrast, intracerebroventricular administration of an 8-fold lower dose of AG10-L2-Oxy resulted in effective analgesia (latency = 54.2 ± 4.8 seconds) and major inhibition of GI transit (9.5 ± 2.8%). This highlights some of the caveats of using short-acting opioids for performing in vivo mechanistic studies and provides a clear advantage of the longer duration of the AG10-based probes when performing in vivo experiments.

We also evaluated the efficacy of the long-acting antidiarrheal agent loperamide in inducing constipation in our OIC rat model. Loperamide enters the brain readily but due to efflux by P-glycoprotein its CNS concentration is minimal. At therapeutic doses, the action of loperamide is peripherally restricted to the mu-opioid receptors in the gut. However, at higher doses loperamide can overwhelm the efflux transporter, accumulate in the brain, and lead to opioid-related CNS adverse effects including overdose and death^46^. In contrast, fentanyl (a lipophilic drug with high CNS penetration) has a 2-fold preference for the brain over plasma^47^. Similar to fentanyl, loperamide has high potent agonist activity on the mu-opioid receptors (*K*_i_ = 0.53 nM vs. *K*_i_ = 0.39 nM for fentanyl)^48^. The reported ED_50_ of subcutaneous loperamide for inducing constipation is 1 mg/kg (GI transit = 50%)^49^. Therefore, we evaluated the efficacy of loperamide vs. fentanyl in inducing OIC at a dose of 1 mg/kg. In our study, the inhibition of GI transit by loperamide (GI transit = 54.5 ± 4.9%, which is consistent with literature) was 25-fold lower than that of fentanyl (GI transit = 2.3 ± 1.8%) (Supplementary Fig. 12). Increasing the dose of loperamide by 30-fold (30 mg/kg) has been reported to result in less than 2-fold decrease in GI transit (GI transit = 30%)^49^. It is important to note that the anticholinergic action of loperamide (through a nonopioid mediated process)^50^ may also contribute to the inhibition of the GI transit observed in our studies.

Collectively, these data (Figs. 6–8) provide insights that highlight the critical role of mu-opioid receptors, in both CNS and peripheral nervous system, in causing OIC. Importantly, our findings on the critical role of the mu-opioid receptors in the CNS for causing OIC challenge the theory that morphine and other opioid agonists reduce GI motility through a mechanism involving predominantly the mu-opioid receptors in the GI tract and that the CNS contribution to constipation is minor^17–23^. This information provides clues that explain deficiencies associated with current PAMORAs.

### Evaluating the potential of AG10-L2-Nal as a therapeutic agent for OIC

The data above clear some of the misunderstandings about the mechanism of action of clinically used PAMORAs and highlight the challenges in designing PAMORAs that provide effective reversal of OIC while maintaining adequate levels of analgesia. While AG10-L2-Nal displayed lower efficacy than other PAMORAs in reversing OIC, we thought that maintaining the full analgesic effect of morphine and the extended circulation half-life are important characteristics that warrant further evaluation of AG10-L2-Nal as a potential OIC therapeutic. To replicate a real-life situation, we evaluated the subcutaneous route of administration and challenged AG10-L2-Nal and naloxegol with two doses of morphine.

**Fig. 9:**
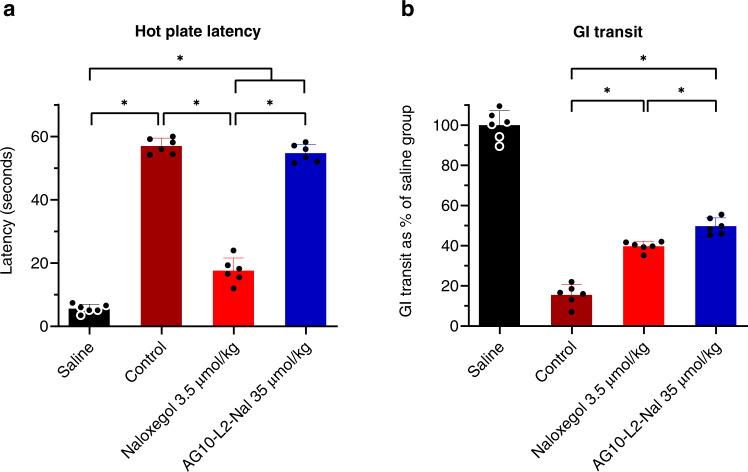
Hot plate and GI transit efficacy studies after subcutaneous administration of AG10-L2-Nal and two doses of morphine. **a** Evaluation of AG10-L2-Nal (single subcutaneous dose; 35 μmol/kg) and naloxegol (single subcutaneous dose; 3.5 μmol/kg) efficacy in reversing analgesia, statistical differences were determined using one-way ANOVA followed by Tukey’s post hoc test F(3,20) = 510.4, *P* < 0.0001 and/or **b** opioid-induced constipation (OIC) caused by two subcutaneous doses of morphine (2 × 35 μmol/kg) in male Sprague-Dawley rats. The saline group received 0.9% sterile saline at 0 h, vehicle at 5 min, and another 0.9% sterile saline dose at 1.5 h. The control group received 35 μmol/kg morphine at 0 h, followed by vehicle at 5 min, and a second morphine dose (35 μmol/kg) at 1.5 h. All other groups received 35 μmol/kg morphine at 0 h, followed by naloxegol (3.5 μmol/kg) or AG10-L2-Nal (35 μmol/kg) at 5 min, and a second morphine dose (35 μmol/kg) at 1.5 h. The hot plate latency was measured after 2.5 h of the first saline or morphine dose at 55 ± 0.5 °C temperature. For the Gastrointestinal (GI) transit assay, 1 mL of charcoal meal was given by oral gavage 30 min after the first saline/morphine dose. GI transit was measured after 2.5 h of the first saline or morphine dose. Statistical differences were determined using one-way ANOVA followed by Tukey’s post hoc test F(3,20) = 301.8, *P* < 0.0001. All data are presented as mean (±s.d.) (*P < 0.05, *n* = 6 rats per group). Source data are provided as a Source Data file.

There was no significant difference between the control (2 doses of morphine) and AG10-L2-Nal (AG10-L2-Nal + 2 doses morphine) groups in hot plate latencies (57.0 ± 2.5 and 54.7 ± 2.8 seconds, respectively) (Fig. 9a). The latency for naloxegol (3.5 μmol/kg; the only dose that maintained some level of analgesia, see Fig. 6) was 17.6 ± 4.0 seconds, which is higher than that of the saline group (5.6 ± 1.3 seconds) but much lower than that of AG10-L2-Nal and morphine groups. These results indicate that AG10-L2-Nal does not reverse opioid-induced analgesia after 2.5 h when tested against two equimolar doses (35 µmol/kg) of morphine (Fig. 9a). We also demonstrated that AG10-L2-Nal significantly reversed morphine-induced constipation (Fig. 9b). The gastrointestinal motility for AG10-L2-Nal was 49.8 ± 4.1% of the drug-free saline group, which is significantly higher than both naloxegol (39.7 ± 2.5%) and control groups (15.6 ± 5.1%). The OIC reversal by AG10-L2-Nal after 2.5 h of the subcutaneous dosing is more than what we observed at 1 h after the intravenous dose, mainly due to AG10-L2-Nal high binding affinity to opioid receptors and longer circulation half-life.

In addition to being stable in human, rat, and mouse sera (Fig. 5a and Supplementary Fig. 7), we found AG10-L2-Nal to be chemically stable in phosphate buffer saline for at least one month at 37 °C (Supplementary Fig. 13). Our data also showed that AG10-L2-Nal does not interfere with TTR’s biological function and that TTR can indeed interact with both AG10-L2-Nal and holo-RBP in concert (Supplementary Fig. 14). AG10-L2-Nal showed no cytotoxic effects toward two cell lines tested (Jurkat and Hep3B cell lines; Supplementary Fig. 15). Therefore, AG10-L2-Nal displayed excellent chemical stability, pharmacokinetics, and pharmacodynamic properties, potentially making it an effective therapeutic candidate for treating OIC.

## Discussion

For the last four decades, a growing number of investigations into sites of action of opioid analgesics have utilized quaternary PAMORAs, which have been assumed to have limited permeability across the BBB. On the other hand, several preclinical data and clinical reports have indicated that these quaternary PAMORAs, or their active parent tertiary metabolites, may penetrate the BBB quite readily, and therefore, the results obtained are at best difficult to interpret^11,15,16,51^. In many of the published reports, variables such as the type of opioid used and its duration of action, potency, and metabolic stability^11^ have not been fully considered in the design of in vivo experiments. These variations made it difficult to draw conclusions about the exact role of the peripheral nervous system and CNS in mediating analgesia and other side effects of opioid medications. For example, it was suggested that morphine and other opioid agonists reduce GI motility through a mechanism involving predominantly the mu-opioid receptors in the GI tract and that the CNS contribution to constipation is minor^17–23^. This was based on experiments where OIC was fully reversed by PAMORAs (assumed incorrectly to act only peripherally) that are concomitantly administered with morphine. We show here that the assumption that OIC reversal is predominantly driven by antagonism of peripheral mu-opioid receptors is incorrect. Our drug delivery system allowed us to develop AG10-L2-Nal, which displayed superior binding potency, peripheral selectivity, pharmacokinetics, and efficacy in rats compared to other clinically used PAMORAs. Our AG10-L2-Nal data demonstrate that the mu-opioid receptors in the CNS, in synergy or additive effect with the peripheral nervous system, have a fundamental role in precipitating OIC. This is supported by experiments with our AG10-L2-Oxy agonist, where intracerebroventricular administration induced substantial constipation while SC administration induced limited constipation. Importantly, the ability to restrict our probes either in the peripheral (by SC or IV administrations) or central compartments (by ICV dosing) provide these molecules with a major advantage over other opioid agonist/antagonist probes that enter or exit the brain at variable rates (e.g., oxycodone vs. morphine), requiring these pharmacokinetic variables to be evaluated carefully before each in vivo experiment.

In our studies in rats, it was surprising to see that a significant percentage of the doses of PAMORAs, currently used clinically to treat OIC, cross the BBB. This could explain the occurrence of opioid withdrawal symptoms and reversal of central analgesic effect in some patients. When given concomitantly with morphine, AG10-L2-Nal reduced constipation-related side effects while maintaining comparable levels of analgesia to that of morphine. The high peripheral selectivity of AG10-L2-Nal suggests that it would not precipitate withdrawal symptoms when given with opioid analgesics. Most of the approved PAMORAs are limited to non-cancer patients, and subcutaneous MNTX is the only PAMORA approved for OIC in cancer patients^15^. However, a significant number of patients do not respond to MNTX or experience withdrawal symptoms. Therefore, AG10-L2-Nal has an immediate potential use as a therapeutic agent for OIC in patients with chronic non-cancer and chronic cancer pain where there is a need for higher doses of opioids to obtain continuous analgesia. The bioavailability of AG10-L2-Nal via the subcutaneous route has a special importance for cancer patients where the subcutaneous route of administration is preferred, especially for patients with dysphagia (i.e., swallowing difficulties)^52^.

At certain low concentrations, both naloxegol and MNTX maintained some level of analgesia (latency ~15 seconds) and partial reversal of OIC (GI transit = 30–50%). While this level of efficacy might be clinically effective for certain patient population, this could also explain why some patients respond poorly to these medications. For example, subcutaneous MNTX is the only PAMORA approved for OIC in cancer patients^15^. However, a significant number of patients do not respond to MNTX or experience withdrawal symptoms. By extending the half-life of AG10-L2-Nal, we have shown that a similar or better reversal of OIC than current PAMORAs is possible, while at the same time maintaining full analgesia.

We believe that AG10-L2-Nal and AG10-L2-Oxy or their chemotypes offer promise as future neurochemical tools for investigating the actions of opioids and for studying the mu-opioid receptor as a potential therapeutic target for yet unexplored applications. For example, PAMORAs have been investigated both preclinically and clinically in counteracting the effect of opioid agonists on promoting cancer growth and metastasis, HIV and hepatitis C virus infection, urinary retention, postoperative ileus, and rheumatoid arthritis progression^9,24,25^. Therefore, these exciting possibilities of using selective PAMORAs that do not interfere with the intended analgesia of opioid agonists await further investigation. In summary, we have developed a drug delivery approach for restricting the passage of small molecules across the BBB. We demonstrated the utility of our approach by developing the most potent and peripherally selective opioid probes. Our findings could help in the discovery of a class of therapeutic agents or pharmacological probes, which should broaden the scope and utility of our approach.

## Methods

### Evaluation of binding affinity of ligands to TTR in buffer

The binding affinity of compound **1**, AG10-L1-Nal, AG10-L2-Nal, and AG10-L2-Oxy to TTR in buffer was determined by their ability to displace FP probe from TTR using a Fluorescence Polarization (FP) assay^32^. Serial dilutions of compound **1**, AG10-L1-Nal, AG10-L2-Nal, and AG10-L2-Oxy (0.010 µM to 20 µM) were added to a solution of FP-probe (50 nM) and TTR (300 nM) in assay buffer (PBS pH 7.4, 0.01% Triton-X100, 1% DMSO in 25 μL final volumes) in a 384-well plate. The samples were allowed to equilibrate by agitation on a plate shaker for 20 minutes at room temperature. Fluorescence polarization (excitation λ 485 nm, emission λ 525 nm, cutoff λ 515 nm) measurements were taken using a SpectraMax M5 Microplate Reader (Molecular Devices). We used SoftMax® Pro software v5.4.1 (Molecular Devices, Inc.) to collect the fluorescence data. The IC_50_ values were obtained by fitting the data to the following equation [y = (A-D)/(1 + (x/C)^B) + D], where A = maximum FP signal, B = slope, C = apparent binding constant (*K*_app_), and D = minimum FP signal. The binding constant (*K*_d_) values were calculated using the Cheng–Prusoff equation from the IC_50_ values. All reported data represent the mean ± s.d. (*n* = 3).

### Evaluation of binding affinity and selectivity of ligands to TTR in human serum

The binding affinity and selectivity of ligands compound **1**, AG10-L1-Nal, AG10-L2-Nal, and AG10-L2-Oxy to TTR in human serum was determined by their ability to compete with the binding of a fluorescent probe exclusion (FPE probe) binding to TTR in human serum^33,34^. AG10 and tafamidis were used as controls. An aliquot (98 µL) of human serum was mixed with 1 μL of test compounds (1.0 mM stock solution in DMSO; 10 µM final concentration in serum) and 1 μL of FPE probe (0.36 mM stock solution in DMSO; 3.6 µM final concentration in serum). The fluorescence changes (λ_ex_ = 328 nm and λ_em_ = 384 nm) were monitored every 15 min using a SpectraMax M5 microplate reader for 6 h at 25 °C.

### Evaluation of the brain uptake of the experimental compounds in rats

Jugular vein cannulated male Sprague Dawley rats were used for this study. Animals were randomized in various treatment groups (*n* = 3 animals per group). Each animal received one 50 µmol/kg intravenous bolus dose of compound **1** or 4.84 µmol/kg dose of naloxone, naloxegol, or AG10-L2-Nal in 200 µL dosing solution, followed by an injection of 200 µL sterile saline to flush the jugular vein cannula. Oxycodone (16 µmol/kg) and AG10-L2-Oxy (16 & 64 µmol/kg) doses were administered subcutaneously. The doing solution (vehicle) was composed of 10% DMSO, 20% PEG400 and 70% sterile deionized water. 30 min (for compound **1**, naloxone, naloxegol and AG10-L2-Nal) or 60 min (for oxycodone and AG10-L2-Oxy) after the dosing, the rats were anesthetized with an intraperitoneal injection of 90 mg/kg ketamine and 9 mg/kg xylazine. The CSF samples were collected from the cisterna magna with a 22-gauge needle. The blood was collected from the aortic exsanguination with a 20-gauge needle followed by decapitation and brain collection. The brain was immediately snap frozen in the liquid nitrogen.

### Evaluation of the intravenous and subcutaneous pharmacokinetic profile of naloxone, naloxegol, and AG10-L2-Nal in rats

Jugular vein cannulated male Sprague Dawley rats (225–250 g; 7-8 weeks old) from Charles River were used for both intravenous (IV) and subcutaneous (SC) pharmacokinetic (PK) studies. For the IV PK study, each animal received one intravenous bolus dose of either naloxone, naloxegol, or AG10-L2-Nal (4.84 µmol/kg) in 200 µL dosing solution followed by an injection of 200 µL sterile saline to flush the jugular vein cannula (*n* = 3 rats per group). 200 µL blood samples were collected from each rat, via jugular vein cannula, in heparinized tubes at predetermined time points (0.033, 0.5, 1, 2, 4, 6, 8, 12, and 24 h postdosing), and the volume was replaced with sterile normal saline. For the SC PK study, each animal received one subcutaneous 16 µmol/kg dose of either naloxone, naloxegol, or AG10-L2-Nal, in 500 µL dosing solution (per 250 g rat) in the scruff area. Blood samples were collected from each rat via jugular vein cannula, in heparinized tubes at predetermined time points (0.033, 0.25, 0.5, 0.75, 1, 2, 4, 6, 8, 12, and 24 h postdosing), and the same volume was replaced with sterile normal saline. For the oxycodone and AG10-L2-Oxy SC PK study, 500 µL dosing solution was administered (per 250 g rat) in the scruff area and blood samples were collected at 0.033, 0.5, 0.75, 1, 2, 4, 6, 8, 12, and 24 h. The blood samples were prepared the same way as described in the brain uptake study.

#### LC-MS/MS analysis and sample preparation

The plasma analysis of naloxone, naloxegol, AG10-L2-Nal, oxycodone, and AG10-L2-Oxy were performed the same way as the plasma samples from the brain uptake study of these molecules. A non-compartmental analysis model using Phoenix WinNonlin (version 8) was used to obtain all the intravenous and subcutaneous pharmacokinetic parameters from their plasma concentration−time data.

### Hot plate analgesia assays

Hot plate analgesia meter from Columbus Instruments, Ohio, USA was used for the experiment. The hot plate was maintained at 55 ± 0.5 °C. The maximum length of hot plate exposure was set at 60 seconds to avoid any kind of tissue damage. The withdrawal latency to heat exposure (withdrawal or shaking of the hind paw, sharp withdrawal, licking of fore or hind paw, or attempting to escape by jumping) was recorded and the animal was gently and quickly removed from the hot plate. A footswitch connected to the analgesia meter was used to control the timer. Experimenters were blinded to treatment groups. Pre-dose control response was measured before dosing to establish baseline withdrawal latencies. Predose baseline latencies were ranked, and the animals were allocated to treatment groups, so the mean baseline latencies were similar among groups. The day before the experiment, the rats were placed on the hot plate at normal ambient temperature for half an hour to let them get accustomed to the instrument. The temperature in the experimental room was 22 ± 2 °C. Relative humidity was not controlled in the experiment room while performing the experiment. The rats went through a short fasting (8–12 h) when there was no reported corticosterone hormone level fluctuation, to minimize the stress associated with fasting during these behavior experiments.

### Gastrointestinal (GI) transit assays

The well-established rat GI transit model was employed to determine the ability of opioid antagonists to antagonize morphine-induced constipation by measuring the distance traveled by a charcoal within the GI tract of the fasted male SD rats. Rats were fasted for a short period of time (8–12 h) to minimize the stress associated with fasting during the experiment^53,54^. The charcoal meal was prepared by 10% w/v charcoal and 10% w/v gum Arabic in tap water.

### Statistical Analyses

Data were analyzed using the GraphPad Prism V9.3.1.471 software (La Jolla, CA, USA). All results were expressed as mean ± standard deviation (s.d.). A priori analysis revealed that to achieve a statistical power of 0.8 with alpha equal to 0.05, given our experimental s.d. values, the number of animals per group must be more than 2. The power analysis was performed by G*Power software (version 3.0.10). For data meeting normality tests using the Shapiro-Wilk test, statistical difference was determined using parametric tests (two-tailed unpaired t-test with equal variance or one-way/two-way ANOVA followed by Tukey’s multiple comparison test). For nonparametric data, statistical differences were determined using the Kruskal-Wallis test followed by Dunn’s multiple comparisons test to identify which groups showed statistical difference. All statistical analyses were indicated in the figure legends or in the tables. Significant differences were determined at (*) *P* < 0.05.

### Reporting summary

Further information on research design is available in the Nature Research Reporting Summary linked to this article.

## Supplementary information

Supplementary Information

Reporting Summary

## Data Availability

The authors declare that all the data supporting the findings of this study are available within the article and Supplementary Information files. The publicly available protein structures can be found in pdb id: 4HIQ and 4DKL. All compounds (including AG10-L1-Nal, AG10-L2-Nal, and AG10-L2-Oxy) can be obtained through a standard material transfer agreement by contacting malhamadsheh@pacific.edu. Source data are provided with this paper.

## Source data

Source Data
