# Supplementary material for: Peripherally restricted transthyretin-based delivery system for probes and therapeutics avoiding opioid-related side effects
Source: Nat Commun. 2022 Jun 23;13:3590. doi: 10.1038/s41467-022-31342-z (PMC9226319; doi:10.1038/s41467-022-31342-z)
Supplement: Supplementary file 1 — Supplementary Information [file 41467_2022_31342_MOESM1_ESM.pdf]

# Supplementary Information for

## Peripherally restricted transthyretin-based delivery system for probes and therapeutics avoiding opioid-related side effects

Md Tariqul Haque Tuhin[1,2], Dengpan Liang[1,2], Fang Liu[1,2], Hala Aldawod[1], Toufiq Ul Amin[1], Joshua S. Ho[1], Rasha Emara[1], Arjun D. Patel[1], Melanie A. Felmlee[1], Miki S. Park[1], James A. Uchizono[1], Mamoun M. Alhamadsheh[1]*

[1]Department of Pharmaceutics and Medicinal Chemistry, Thomas J. Long School of Pharmacy, University of the Pacific, Stockton, California 95211, United States

[2]These authors contributed equally to this work.

*Corresponding author. Email: malhamadsheh@pacific.edu

# Table of Contents


| Supplementary Figures 1-19 | |
|---|---|
| • **Supplementary Figure 1.** LC-MS/MS calibration curves used to quantitate compound **1** in rat plasma, brain, and CSF | 5 |
| • **Supplementary Figure 2.** Human mu-opioid receptor binding (agonist radioligand) assays of naloxone, naloxegol, AG10-L1-Nal, AG10-L2-Nal, oxycodone, and AG10-L2-Oxy | 6 |
| • **Supplementary Figure 3.** Human mu-opioid receptor guanosine 5-O-(3-[$^{35}$S]thio)triphosphate ([$^{35}$S]GTP$\gamma$S) antagonist functional binding assays of **(a)** AG10-L2-Nal, and **(b)** naloxegol | 7 |
| • **Supplementary Figure 4.** Competitive antagonism of morphine by AG10-L2-Nal at the human mu-opioid receptor | 8 |
| • **Supplementary Figure 5.** Human delta-opioid receptor (agonist radioligand) binding assays of AG10-L2-Nal and AG10-L2-Oxy | 9 |
| • **Supplementary Figure 6.** Rat kappa-opioid receptor (agonist radioligand) binding assays of AG10-L2-Nal and AG10-L2-Oxy | 9 |
| • **Supplementary Figure 7.** HPLC traces for the stability study of AG10-L2-Nal in **(a)** PBS buffer, **(b)** human serum, **(c)** rat serum, and **(d)** mouse serum at 0 hour, 24 hours, and 48 hours | 10 |
| • **Supplementary Figure 8.** LC-MS/MS calibration curves used to quantitate naloxone, naloxegol, and AG10-L2-Nal in rat plasma, brain, and CSF | 11 |
| • **Supplementary Figure 9.** Hot plate and gastrointestinal (GI) transit efficacy studies of AG10-L2-Nal with AG10 against morphine | 12 |
| • **Supplementary Figure 10.** Hot plate and gastrointestinal (GI) transit efficacy studies of AG10-L2-Nal and naloxegol against fentanyl | 13 |
| • **Supplementary Figure 11.** LC-MS/MS calibration curves used to quantitate oxycodone and AG10-L2-Oxy in rat plasma, brain, and CSF | 14 |
| • **Supplementary Figure 12.** Gastrointestinal (GI) transit assay of loperamide. Gastrointestinal (GI) transit assay at 1 hour after SC doses of the test compounds | 15 |
| • **Supplementary Figure 13.** HPLC traces for the long-term stability study of AG10-L2-Nal in PBS buffer at 0-day, 7-day, 14-day, 21-day, and 28-day intervals | 16 |
| • **Supplementary Figure 14.** Binding of AG10-L2-Nal to TTR does not interfere with the holo-RBP binding to TTR | 17 |
| • **Supplementary Figure 15.** MTT cell proliferation assay to determine the cytotoxicity of naloxone, naloxegol, and AG10-L2-Nal in Jurkat and Hep3B cell lines | 18 |
| • **Supplementary Figure 16.** Synthesis of naloxone derivative **7** | 24 |

| | |
|---|---|
| • **Supplementary Figure 17.** Synthesis of compound **11** | 26 |
| • **Supplementary Figure 18.** Synthesis of AG10-L1-Nal and AG10-L2-Nal | 30 |
| • **Supplementary Figure 19.** $^{1}$H NMR Spectrum for AG10-L1-Nal (**2**) | 32 |
| • **Supplementary Figure 20.** $^{13}$C NMR Spectrum for AG10-L1-Nal (**2**) | 32 |
| • **Supplementary Figure 21.** HPLC traces of AG10-L1-Nal (**2**) | 33 |
| • **Supplementary Figure 22.** $^{1}$H NMR Spectrum for AG10-L2-Nal (**3**) | 35 |
| • **Supplementary Figure 23.** $^{13}$C NMR Spectrum for AG10-L2-Nal (**3**) | 35 |
| • **Supplementary Figure 24.** HPLC traces of AG10-L2-Nal (**3**) | 36 |
| • **Supplementary Figure 25.** Synthesis of AG10-L2-Oxy (**4**) | 37 |
| • **Supplementary Figure 26.** $^{1}$H NMR Spectrum for AG10-L2-Oxy (**4**) | 40 |
| • **Supplementary Figure 27.** $^{13}$C NMR Spectrum for AG10-L2-Oxy (**4**) | 40 |
| • **Supplementary Figure 28.** HPLC traces of AG10-L2-Oxy (**4**) | 41 |
| **Supplementary Tables 1-5** | |
| • **Supplementary Table 1.** Mass spectrometer conditions for multiple reaction monitoring (MRM) of the tested compounds. | 19 |
| • **Supplementary Table 2.** Binding affinity of the test compounds to the human mu-opioid receptor. | 19 |
| • **Supplementary Table 3.** Pharmacokinetic parameters of naloxone, naloxegol, and AG10-L2-Nal determined from the plasma concentrations after the intravenous dosing | 20 |
| • **Supplementary Table 4.** Pharmacokinetic parameters of naloxone, naloxegol, and AG10-L2-Nal determined from the plasma concentrations after the subcutaneous dosing | 20 |
| • **Supplementary Table 5.** Pharmacokinetic parameters of Oxycodone and AG10-L2-Nal determined from the plasma concentrations after the subcutaneous dosing | 21 |
| **Supplementary Equations 1-2** | |
| • **Supplementary Equation 1.** Modified Hill equation for four-parameter logistic nonlinear regression model | 46 |
| • **Supplementary Equation 2.** Cheng-Prusoff equation | 47 |
| **Supplementary Methods** | |
| • Materials and reagents | 22 |
| **Chemical synthesis and HPLC purity analysis** | |
| • Chemistry general procedures | 22 |
| • Preparative HPLC method for purification of the synthesized compounds | 23 |
| • Analytical HPLC method for evaluating the purity of the synthesized compounds | 23 |

| | |
|---|---|
| Experimental animals | 42 |
| Sample preparation and LC-MS/MS analysis of the experimental compounds | 43 |
| In silico modeling studies | 45 |
| In vitro pharmacology: opioid receptor binding assays:<br>• Human mu-opioid receptor binding (agonist radioligand) assay<br>• Human delta- and rat kappa- opioid receptor (agonist radioligand) binding assays | <br>46<br>47 |
| Functional mu-opioid receptor activity assays | 48 |
| Serum stability assay of AG10-L2-Nal | 48 |
| Long-term stability assay of AG10-L2-Nal in PBS buffer | 48 |
| Hot plate analgesia assays | 49 |
| Gastrointestinal (GI) transit assays | 52 |
| Evaluating the effect of AG10-L2-Nal on Holo-RBP-TTR interaction in human serum | 54 |
| Evaluation of the *in vitro* cytotoxicity of naloxone, naloxegol, and AG10-L2-Nal | 55 |
| Supplementary References | 56 |

# Supplementary Figures

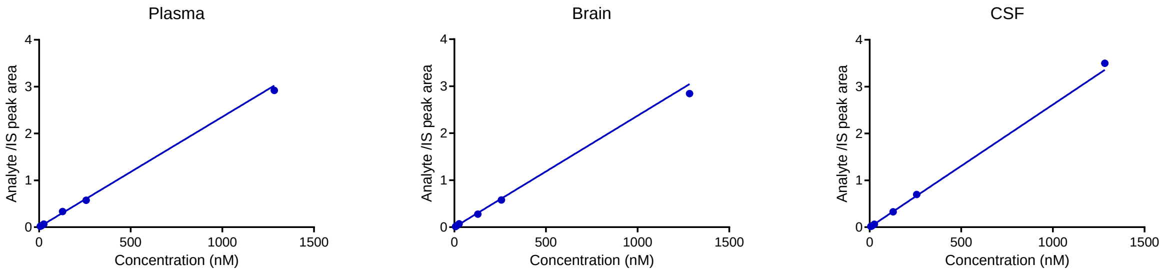


**Supplementary Figure 1.** LC-MS/MS calibration curves used to quantitate Compound **1** in rat plasma, brain, and CSF. The calibration curves were for the BBB study. The identities of the compounds were determined using the following Q1/Q3 transition masses for Compound **1** (390.2/372.1) and IS reference Chloro-AG10 (309.0/109.2). See Supplementary Table 1 for the detailed multiple reaction monitoring (MRM) parameters. Source data are provided as a Source Data file.

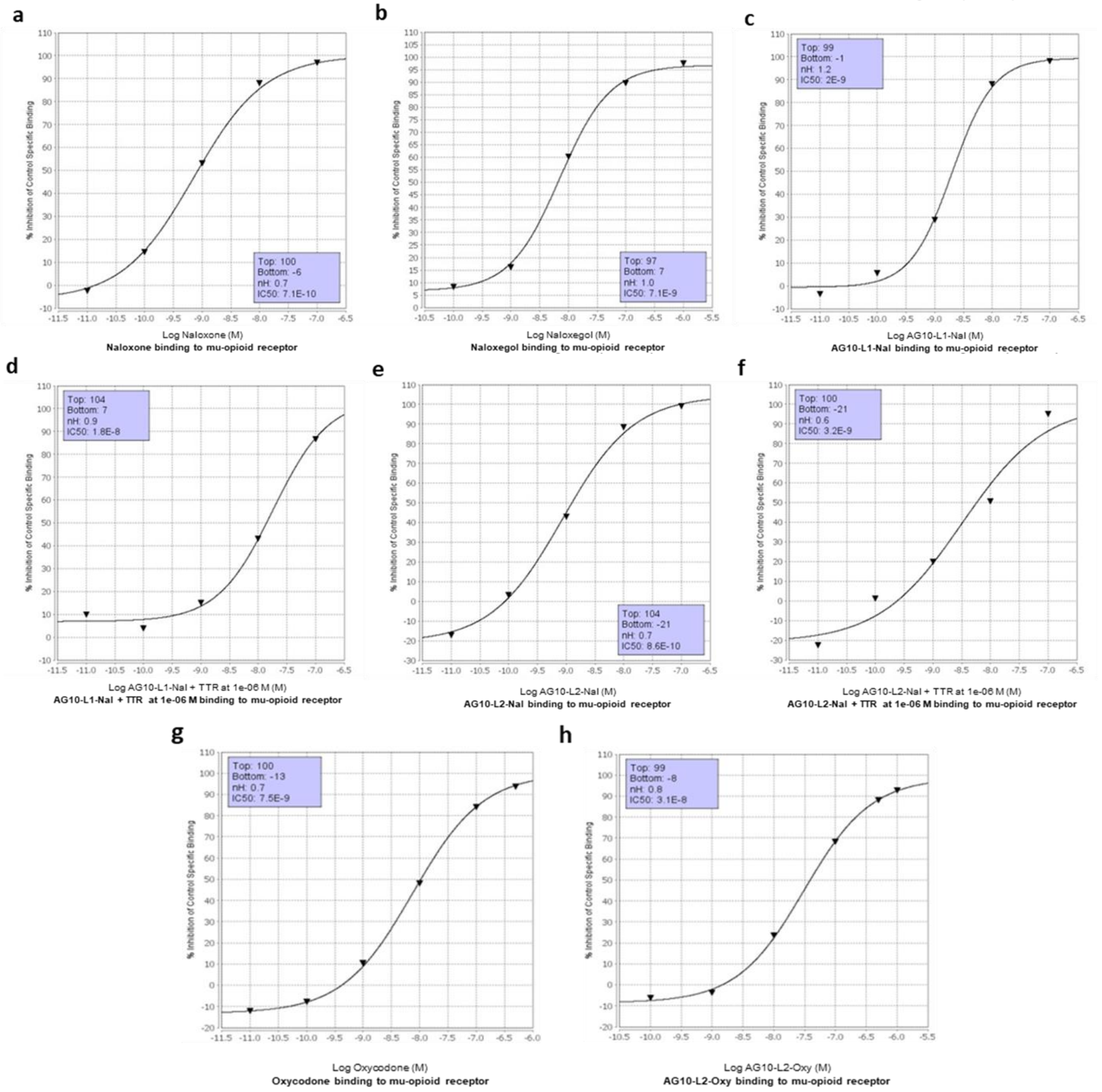


**Supplementary Figure 2.** mu-opioid receptor binding (agonist radioligand) assays of naloxone, naloxegol, AG10-L1-Nal, AG10-L2-Nal, oxycodone, and AG10-L2-Oxy **(a-h).** The inhibitory binding constant ($K_i$) values were calculated using the Cheng–Prusoff equation from $IC_{50}$ values. Source data are provided as a Source Data file.

**a**

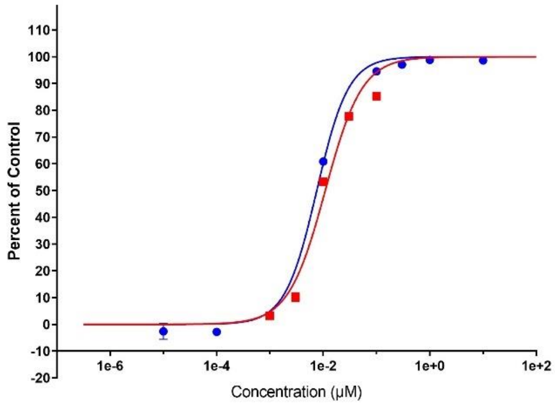


| Compound Name | $IC_{50}$ |
|---|---|
| ■ Naltrexone | 10.9 nM |
| ● AG10-L2-Nal | 7.5 nM |

**b**

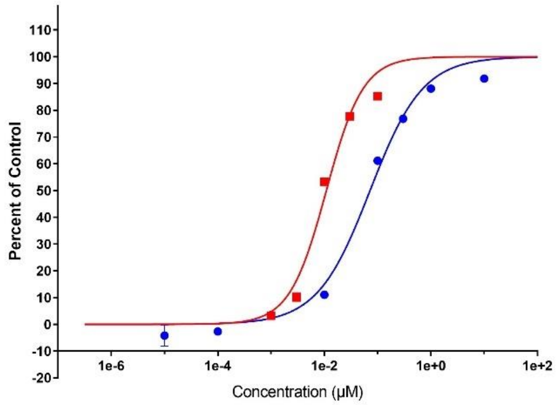


| Compound Name | $IC_{50}$ |
|---|---|
| ■ Naltrexone | 10.9 nM |
| ● Naloxegol | 72 nM |

**Supplementary Figure 3.** Human mu-opioid receptor guanosine 5-O-(3-[$^{35}$S]thio)triphosphate ([$^{35}$S]GTPγS) antagonist functional binding assays of **(a)** AG10-L2-Nal, and **(b)** naloxegol. Antagonistic functional binding activity of human mu-opioid receptors expressed in the membranes of CHO-K1 cells from Chinese hamster ovary was used in these assays. 8 testing concentrations are shown. Error bars indicate mean ± s.d. (Each data point represents mean value of duplicate experiments relative to inhibition of DAMGO-induced bound [$^{35}$S]GTPγS). The potent mu-opioid receptor antagonist, naltrexone, was used as a reference compound in both the assays. Source data are provided as a Source Data file. Error bars are already included in the Figure; however, they are very small for most of the points except the lowest concentrations. The study was conducted at Eurofins, Panlabs (Taipei, Taiwan).

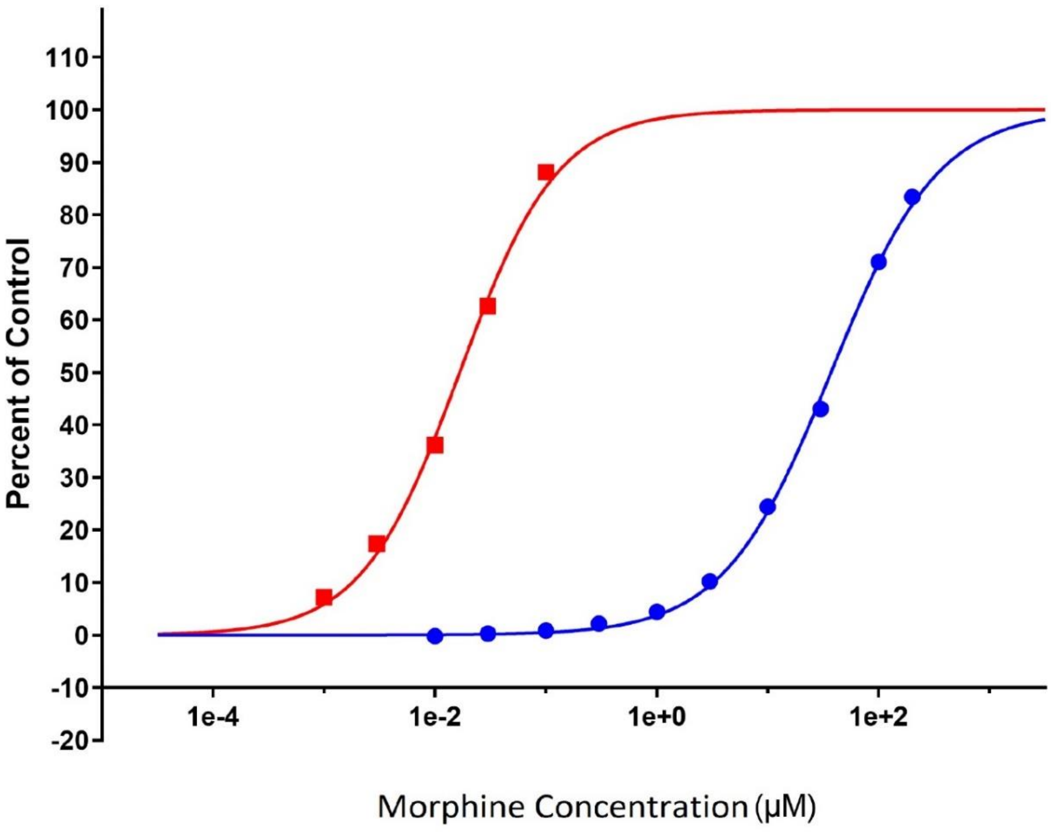


| Compound Name | $EC_{50}$ |
|---|---|
| ■ Morphine | 0.039 µM |
| ● Morphine + 0.4 µM AG10-L2-Nal | 37.2 µM |

**Supplementary Figure 4.** Competitive antagonism of morphine by AG10-L2-Nal at the human mu-opioid receptor. The effect of 0.4 µM AG10-L2-Nal on morphine agonist concentration-response curve, as measured by guanosine 5-O-(3-[$^{35}$S]thio)triphosphate ([$^{35}$S]GTPgS binding) are shown. Agonistic functional binding activity of human mu-opioid receptors expressed in the membranes of CHO-K1 cells from Chinese hamster ovary was used in these assays. 10 testing concentrations are shown. Error bars indicate mean ± s.d. (Each data point represents mean value of duplicate experiments). In the Schild-type experiments, 0.4 µM AG10-L2-Nal elicited parallel rightward shifts in the morphine concentration-response curve with no reduction in the maximal response ($E_{max}$). Source data are provided as a Source Data file. Error bars are already included in the Figure; however, they are very small for all the points. The study was conducted at Eurofins, Panlabs (Taipei, Taiwan).

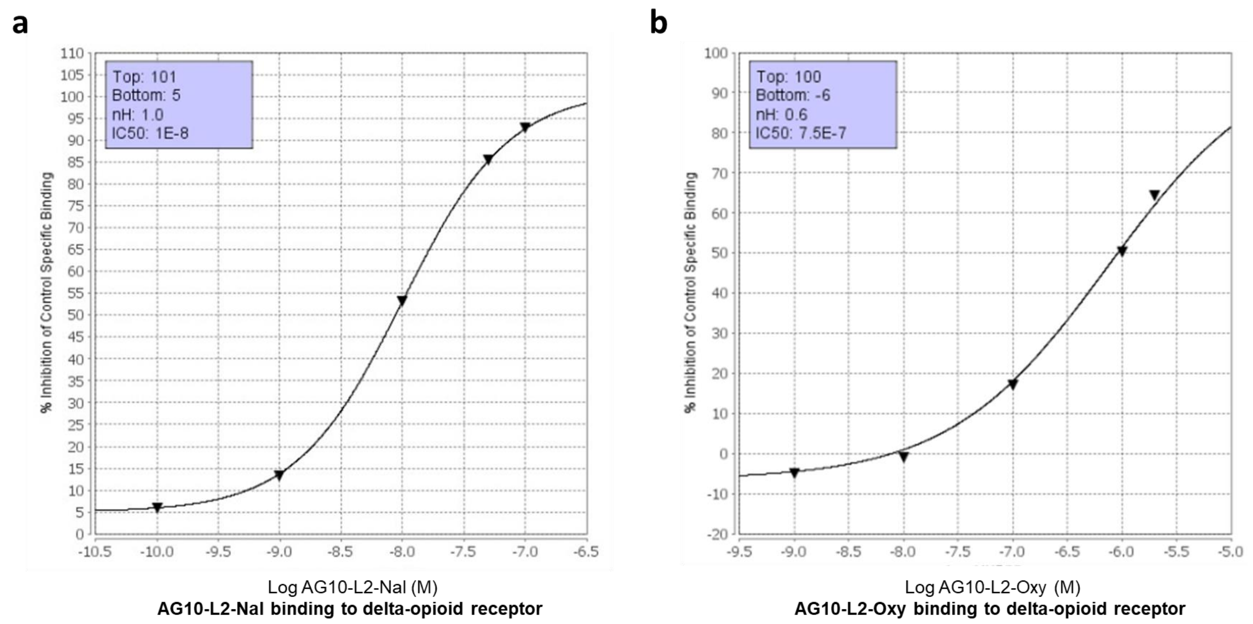


**Supplementary Figure 5.** delta-opioid receptor (agonist radioligand) binding assays of AG10-L2-Nal **(a)** and AG10-L2-Oxy **(b)**. The inhibitory binding constant ($K_i$) values were calculated using the Cheng–Prusoff equation from the $IC_{50}$ values. Source data are provided as a Source Data file.

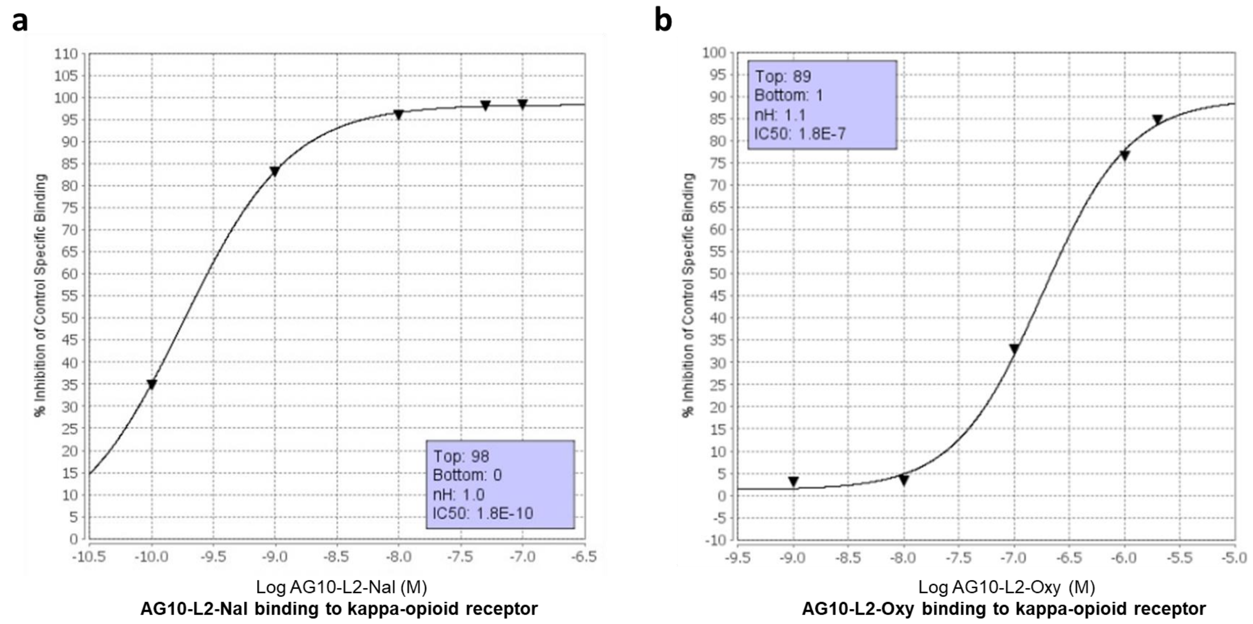


**Supplementary Figure 6.** kappa-opioid receptor (agonist radioligand) binding assays of AG10-L2-Nal **(a)** and AG10-L2-Oxy **(b).** The inhibitory binding constant ($K_i$) values were calculated using the Cheng–Prusoff equation from the $IC_{50}$ values. Source data are provided as a Source Data file.

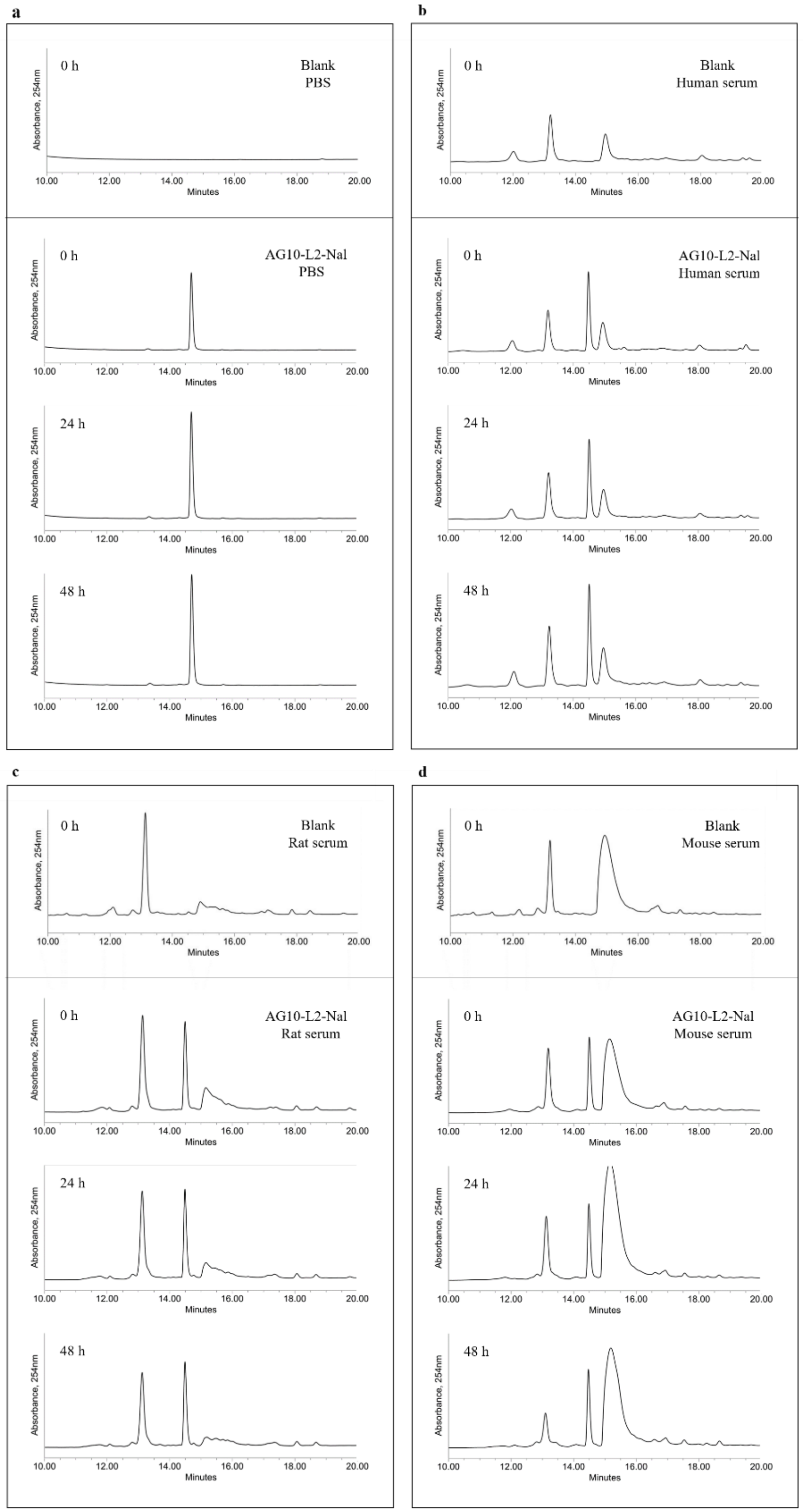


**Supplementary Figure 7.** HPLC traces for the stability study of AG10-L2-Nal in **(a)** PBS buffer, **(b)** human serum, **(c)** rat serum, and **(d)** mouse serum at 0 hour, 24 hours, and 48 hours.

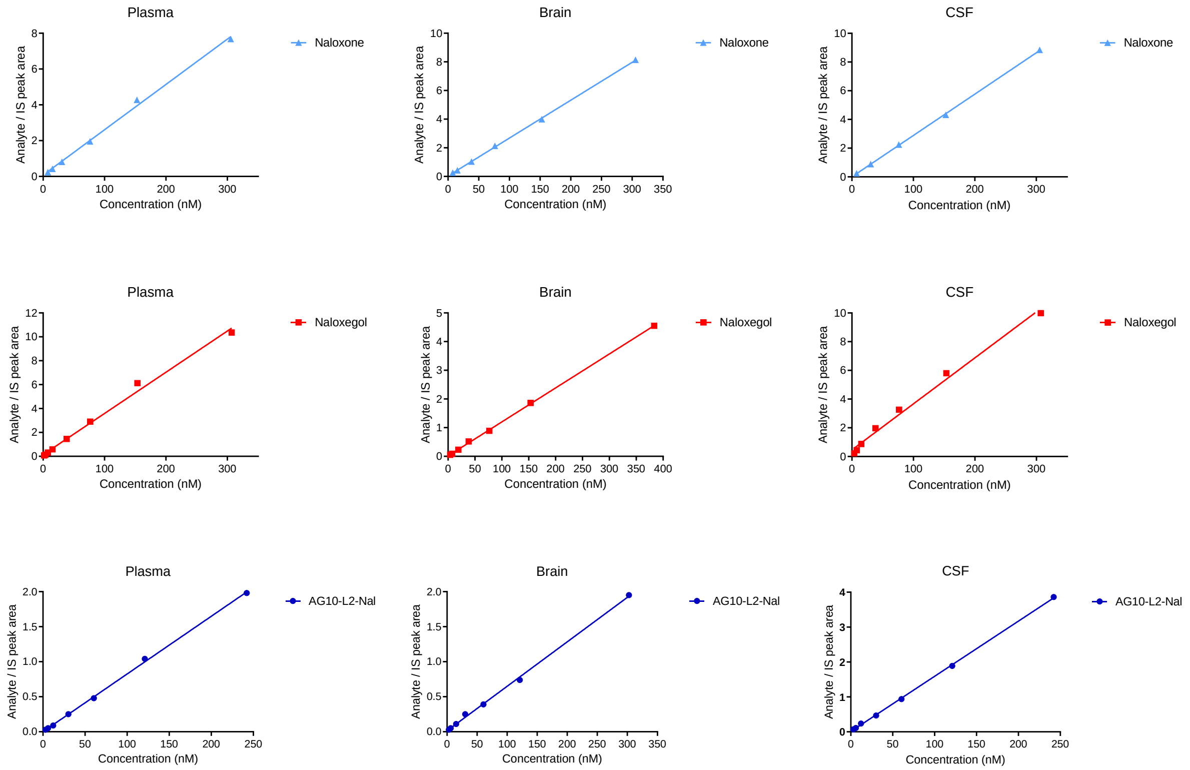


**Supplementary Figure 8.** LC-MS/MS calibration curves used to quantitate naloxone, naloxegol, and AG10-L2-Nal in rat plasma, brain, and CSF. The calibration curves were for the BBB, intravenous, and subcutaneous pharmacokinetic studies. The identities of the compounds were determined using the following Q1/Q3 transition masses for naloxone (328.0/310.0), IS reference naloxone-D5 (333.3/315.0), naloxegol (652.4/634.2), and AG10-L2-Nal (413.9/405). See Supplementary Table 1 for the detailed multiple reaction monitoring (MRM) parameters. Source data are provided as a Source Data file.

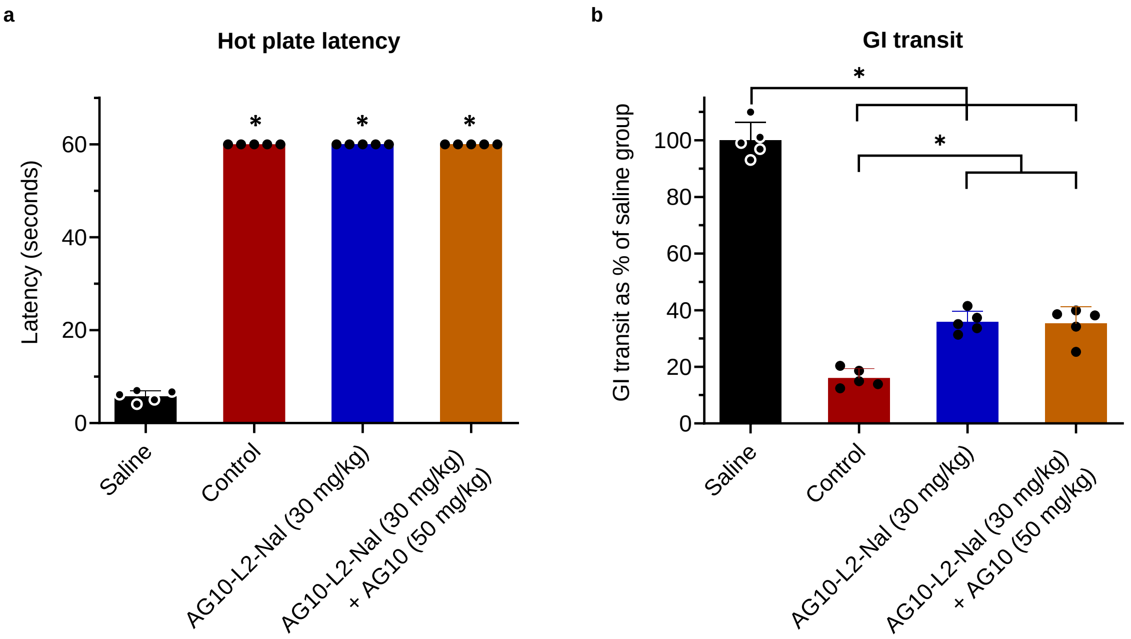


**Supplementary Figure 9. Hot plate and gastrointestinal (GI) transit efficacy studies of AG10-L2-Nal (in the presence and absence of AG10) against morphine.** (**a**) Hot plate latency test to measure analgesia. Male Sprague-Dawley rats were first administered with a single subcutaneous (SC) dose of vehicle or the 50 mg/kg AG10. After 10 minutes, animals were administered with a single subcutaneous dose of saline or 10 mg/kg morphine (35 µmol/kg). After another 5 minutes, animals were administered with AG10-L2-Nal or vehicle. Saline group: vehicle + saline + vehicle; control group: vehicle + 10 mg/kg morphine + vehicle; AG10-L2-Nal group: vehicle + 10 mg/kg morphine (35 µmol/kg) + 30 mg/kg AG10-L2-Nal (35 µmol/kg); AG10-L2-Nal +AG10 group: AG10 50 mg/kg + 10 mg/kg morphine (35 µmol/kg) + 30 mg/kg AG10-L2-Nal (35 µmol/kg). The hot plate withdrawal latency to heat exposure (withdrawal or shaking of the hind paw, sharp withdrawal, licking of fore or hind paw, or attempting to escape by jumping) was recorded 1 hour after the morphine dose before the rats were removed from the hot plate. Statistical differences were determined using Kruskal–Wallis test followed by Dunn's multiple comparisons test, H = 18.53, P = 0.0003. (**b**) Gastrointestinal (GI) transit assay at 1 hour after different SC doses of the test compounds. The dosing schedule is similar to the hot plate assay with an additional oral gavage of charcoal meal 30 minutes after the saline or morphine dose. Statistical differences were determined using one-way ANOVA followed by Tukey's post hoc tes, F(3,16) = 297.5, $P < 0.0001$. All data are presented as mean (± s.d.) (*$P < 0.05$, n = 5 rats per group). Source data are provided as a Source Data file.

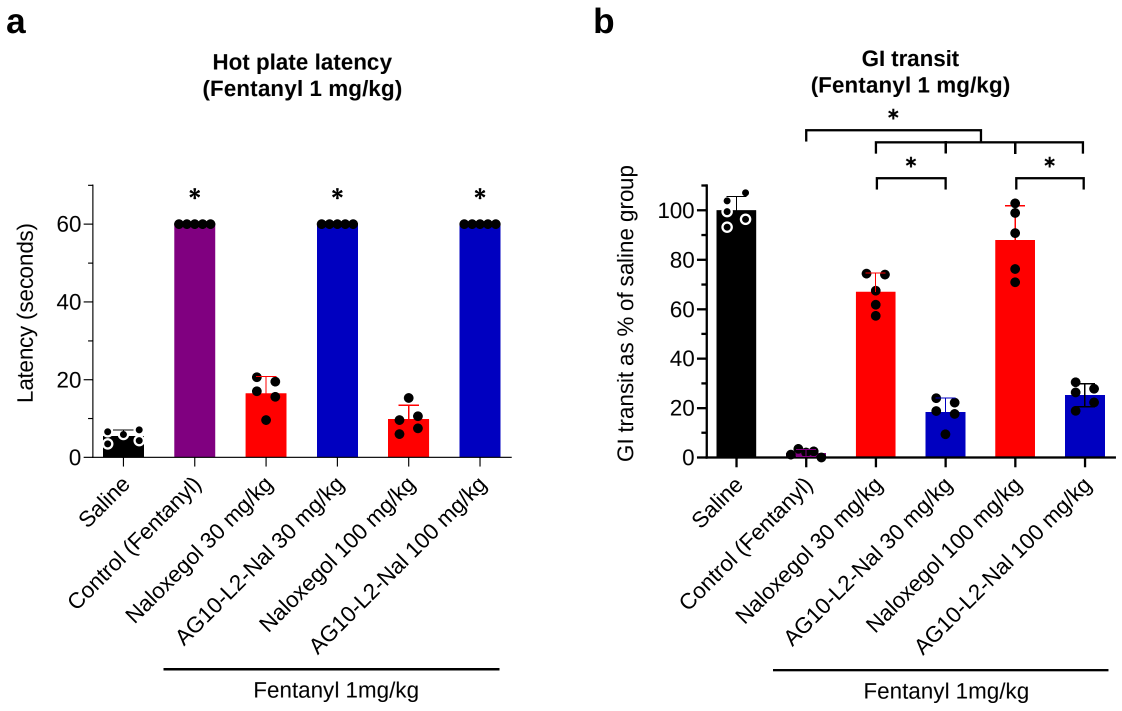


**Supplementary Figure 10.** Hot plate and gastrointestinal (GI) transit efficacy studies of AG10-L2-Nal and naloxegol against fentanyl. (**a**) Hot plate latency test to measure analgesia. Male Sprague-Dawley rats were first administered with a single subcutaneous (SC) dose of vehicle or the opioid antagonists. After 30 minutes, animals were administered with a single subcutaneous dose of saline or fentanyl (1 mg/kg). Saline group: vehicle + saline; control group: vehicle + fentanyl (1 mg/kg); all other groups: specified dose of antagonists + fentanyl (1 mg/kg). The hot plate withdrawal latency to heat exposure (withdrawal or shaking of the hind paw, sharp withdrawal, licking of fore or hind paw, or attempting to escape by jumping) was recorded 1 hour after the fentanyl dose. Statistical differences were determined using Kruskal-Wallis test followed by Dunn's multiple comparisons test, H = 27.93, $P < 0.0001$. All data are presented as mean (± s.d.) (*$P < 0.05$ compared to saline group). (**b**) Gastrointestinal (GI) transit assay at 1 hour after different single SC doses of the test compounds. The dosing schedule is similar to the hot plate assay with an additional oral gavage of charcoal meal 30 minutes after the saline or fentanyl dose. Statistical differences were determined using one-way ANOVA followed by Tukey's post hoc test, $F(5, 24) = 145.5$, $P < 0.0001$. All data are presented as mean (± s.d.) (*$P < 0.05$, n = 5 rats per group). Source data are provided as a Source Data file.

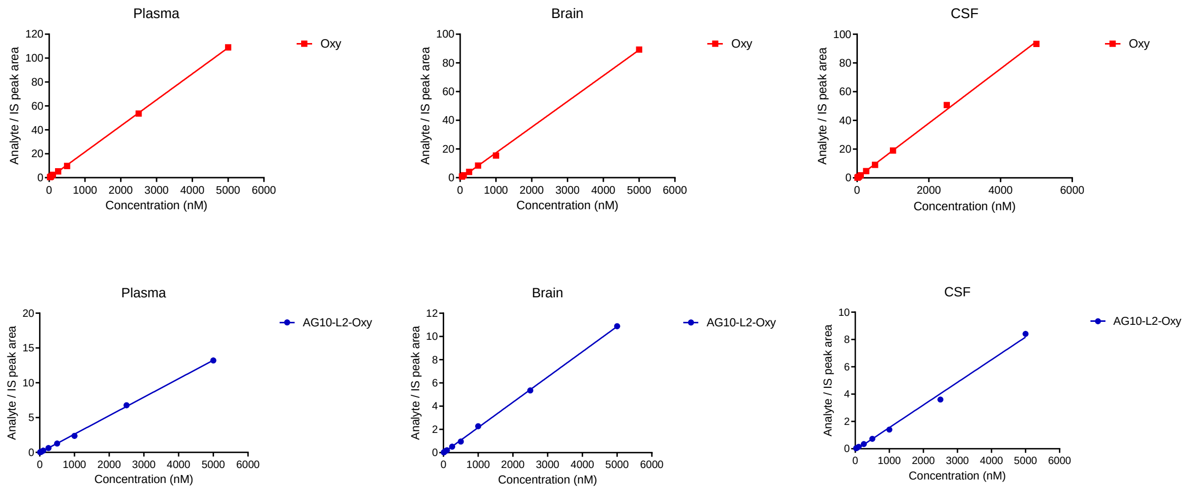


**Supplementary Figure 11.** LC-MS/MS calibration curves used to quantitate oxycodone and AG10-L2-Oxy in rat plasma, brain, and CSF. The calibration curves were for the BBB and subcutaneous pharmacokinetic studies. The identities of the compounds were determined using the following Q1/Q3 transition masses for oxycodone (316.4/241.1), IS reference oxycodone-D6 (322.4/304.5), and AG10-L2-Oxy (814.6/469.1). See Supplementary Table 1 for the detailed multiple reaction monitoring (MRM) parameters. Source data are provided as a Source Data file.

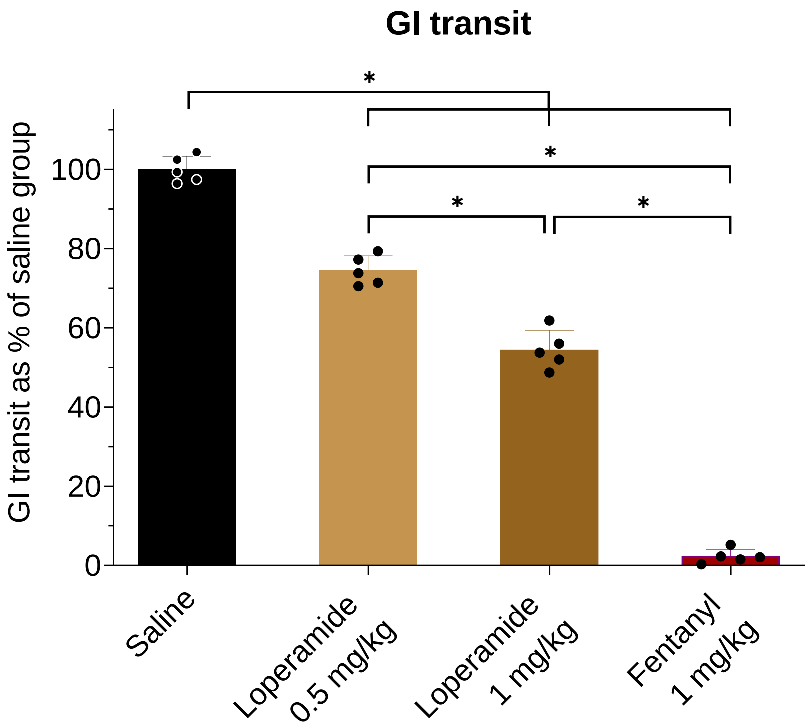


**Supplementary Figure 12.** Gastrointestinal (GI) transit assay of loperamide. Gastrointestinal (GI) transit assay at 1 hour after SC doses of the test compounds. Male Sprague-Dawley rats were first administered with a single subcutaneous (SC) dose of saline or loperamide 0.5 or loperamide 1 mg/kg doses. The animals received an oral gavage of charcoal meal 30 minutes after the saline or loperamide doses. At 1 hour the rats were euthanized and the GI transit was measured. Statistical differences were determined using one-way ANOVA followed by Tukey's post hoc test $F(3,16) = 649.9$, $P < 0.0001$. All data are presented as mean (± s.d.) (*$P < 0.05$, n = 5 rats per group). Source data are provided as a Source Data file.

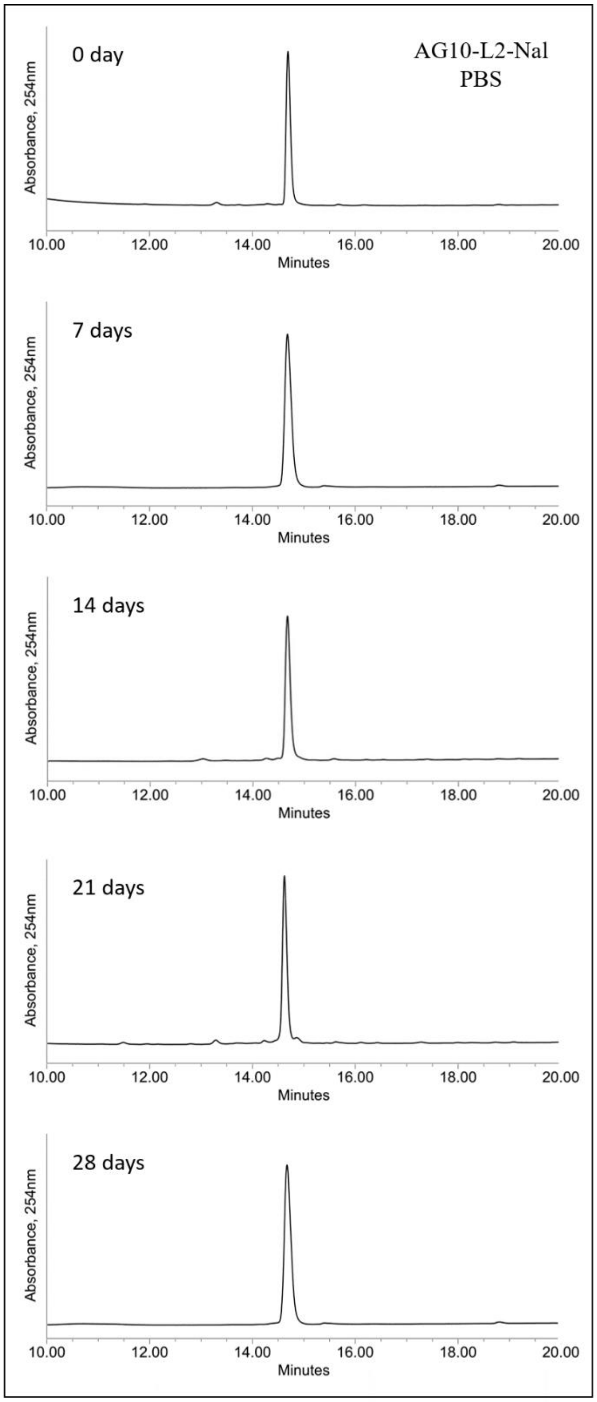


**Supplementary Figure 13.** HPLC traces for the long-term stability study of AG10-L2-Nal in PBS buffer at 0-day, 7-day, 14-day, 21-day, and 28-day intervals.

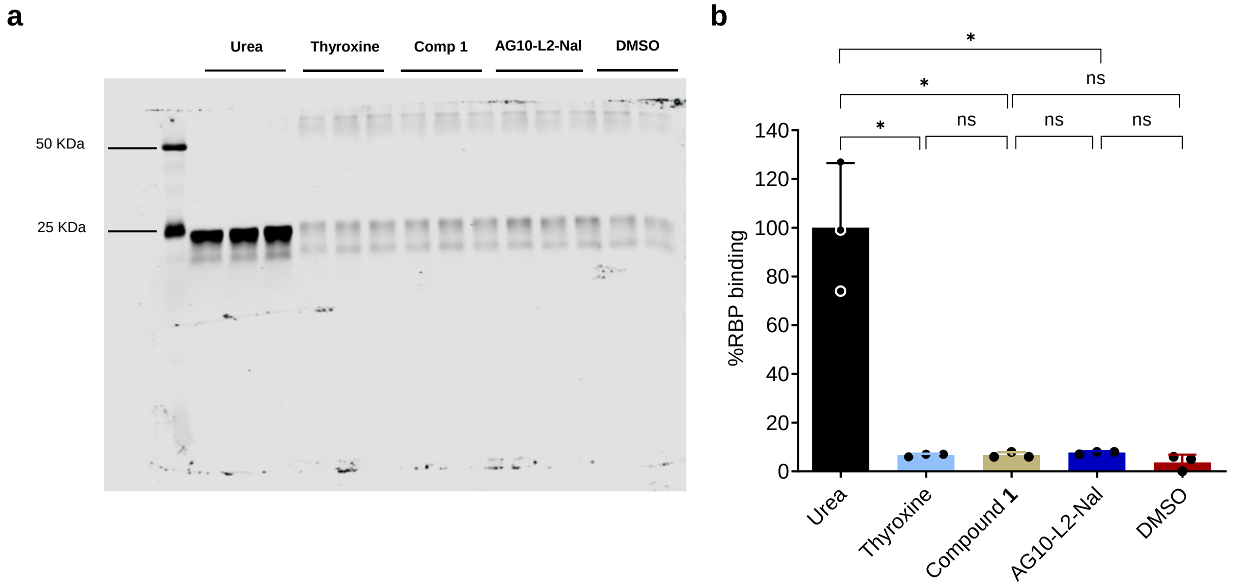


**Supplementary Figure 14.** Binding of AG10-L2-Nal to TTR does not interfere with the holo-RBP binding to TTR. **(a)** Human serum (TTR concentration ~ 5 µM) was incubated with Thyroxine (T4), compound **1**, AG10-L2-Nal (all compounds at 20 µM final concentrations), and DMSO in PBS buffer (pH = 7) or with urea (8 M) buffer for 2 hours at 37°C before cross-linking and immunoblotting. The membrane was incubated with rabbit anti-RBP antibody and then with IRdye800 donkey anti-rabbit secondary antibody. After incubation, the membrane was washed and scanned using LI-COR Odyssey® CLx Imaging System for quantification. The membrane image is a representation of replicate experiment (n=3). **(b)** Bar graph representing the mean (± s.d) (n = 3) of %RBP displacement from TTR quantitated from three membranes. Statistical differences were determined using one-way ANOVA followed by Tukey's multiple comparison test, $F(4,10) = 37.34$, $P < 0.0001$ (*$P < 0.05$). Source data are provided as a Source Data file.

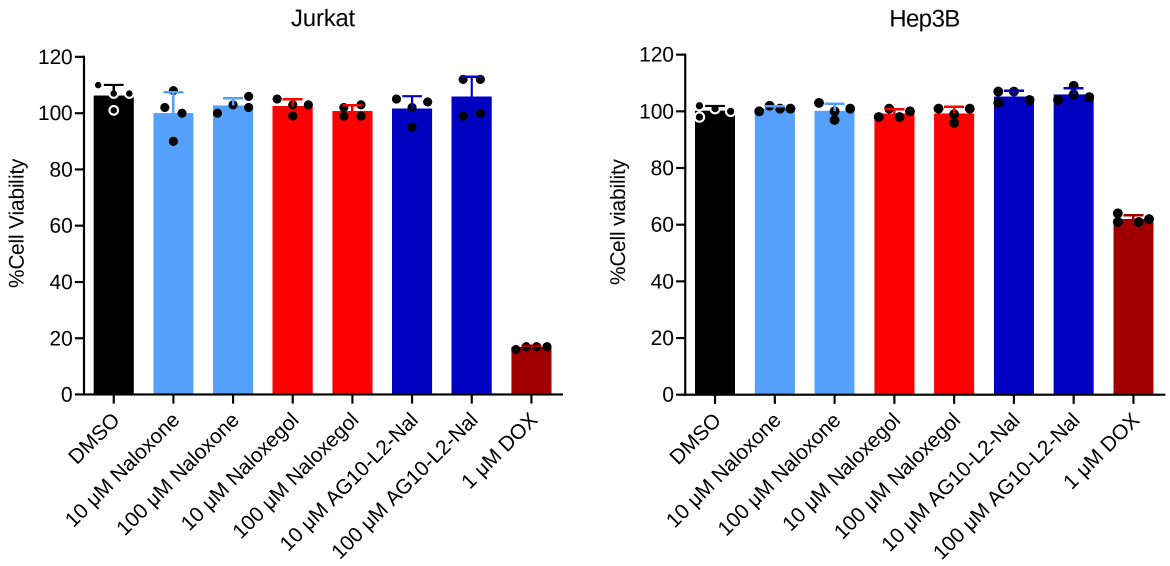


**Supplementary Figure 15.** MTT cell proliferation assay to determine the cytotoxicity of naloxone, naloxegol, and AG10-L2-Nal in Jurkat and Hep3B cell lines. Naloxone, naloxegol, and AG10-L2-Nal showed no cytotoxicity against Jurkat and Hep3B cell lines when tested at 10 and 100 µM concentrations. Each time point is expressed as means ± s.d. (n = 4). Source data are provided as a Source Data file.

# Supplementary Tables

**Supplementary Table 1.** Mass spectrometer conditions for multiple reaction monitoring (MRM) of the tested compounds.

| Compounds | Q1 Mass (Da) | Q3 Mass (Da) | Declustering potential (volts) | Focusing potential (volts) | Entrance potential (volts) | Collision energy (volts) | Collision cell exit potential (volts) |
|---|---|---|---|---|---|---|---|
| Compound **1** | 390.2 | 372.1 | 51 | 170 | 10 | 29 | 24 |
| Chloro-AG10 | 309.0 | 109.2 | 31 | 270 | 10 | 31 | 8 |
| Naloxone | 328.0 | 310.0 | 66 | 220 | 10 | 29 | 8 |
| Naloxone-D5 | 333.3 | 315.0 | 61 | 160 | 10 | 33 | 18 |
| Naloxegol | 652.4 | 634.2 | 66 | 370 | 10 | 45 | 22 |
| AG10-L2-Nal | 413.9 | 405.0 | 36 | 50 | 10 | 21 | 20 |
| Oxycodone | 316.4 | 241.1 | 39 | 150 | 10 | 40 | 30 |
| Oxycodone D6 | 322.4 | 304.5 | 36 | 140 | 10 | 27 | 18 |
| AG10-L2-Oxy | 814.6 | 469.1 | 70 | 293 | 11.5 | 55 | 30 |

**Supplementary Table 2.** Binding affinity of the test compounds to the human mu-opioid receptor. Source data are provided as a Source Data file in Supplementary Figure 2 source data.

| **Compounds** | $K_i$ **(nM)** |
|---|---|
| Naloxone | 0.29 |
| Naloxegol | 2.90 |
| Methylnaltrexone | 22.10 |
| AG10-L1-Nal | 0.81 |
| AG10-L1-Nal + TTR | 7.40 |
| AG10-L2-Nal | 0.35 |
| AG10-L2-Nal + TTR | 1.30 |
| Oxycodone | 3.10 |
| AG10-L2-Oxy | 13.00 |

**Supplementary Table 3.** Pharmacokinetic parameters of naloxone, naloxegol and AG10-L2-Nal determined from the plasma concentrations after intravenous dosing. Statistical differences were determined using one-way ANOVA followed by Tukey's multiple comparison test. For k, F(2,6) = 50.36, $P$ = 0.0002; for $t_{1/2}$, F(2,6) = 90.94, $P$ < 0.0001; for $AUC_{inf}$, F(2,6) = 603.5, $P$ < 0.0001; for CL, F(2,6) = 224.4, $P$ < 0.0001; for $V_{ss}$, F(2,6) = 48.0, $P$ < 0.0001. (* represents $P$ < 0.05 compared to naloxone group, a represents $P$ < 0.05 of naloxegol compared to AG10-L2-Nal group). All data are presented as mean (± s.d.) (n = 3 rats per group). Source data are provided as a Source Data file in Figure 5d source data.

| | k<br>(1/h) | $t_{1/2}$<br>(h) | $AUC_{inf}$<br>(nM.h) | CL<br>(L/h/kg) | $V_{ss}$<br>(L/kg) |
|---|---|---|---|---|---|
| Naloxone | 0.81 ± 0.13 | 0.87 ± 0.13 | 1455.11 ± 257.18 | 3.39 ± 0.55 | 1.23 ± 0.42 |
| Naloxegol | 0.41 ± 0.06 [* a] | 1.72 ± 0.27 [a] | 741.34 ± 33.65 [a] | 6.54 ± 0.29 [* a] | 2.76 ± 0.39 [* a] |
| AG10-L2-Nal | 0.12 ± 0.02 [*] | 5.98 ± 0.81 [*] | 16912.96 ± 1085.27 [*] | 0.29 ± 0.02 [*] | 0.12 ± 0.02 [*] |

**Supplementary Table 4.** Pharmacokinetic parameters of naloxone, naloxegol and AG10-L2-Nal determined from the plasma concentrations after subcutaneous dosing. Statistical differences were determined using one-way ANOVA followed by Tukey's multiple comparison test. For k, F(2,6) = 136.8, $P$ < 0.0001; for $t_{1/2}$, F(2,6) = 41.83, $P$ = 0.0003; for $AUC_{inf}$, F(2,6) = 98.32, $P$ < 0.0001; for CL/F, F(2,6) = 75.43, P < 0.0001; for $V_{ss}$/F, F(2,6) = 4.915, P = .0545. (* represents $P$ < 0.05 compared to naloxone group, a represents $P$ < 0.05 of naloxegol compared to AG10-L2-Nal group). All data are presented as mean (± s.d.) (n = 3 rats per group). Source data are provided as a Source Data file in Figure 5e source data.

| | k<br>(1/h) | $t_{1/2}$<br>(h) | $AUC_{inf}$<br>(nM*h) | CL/F<br>(L/h/kg) | $V_{ss}$/F<br>(L/kg) |
|---|---|---|---|---|---|
| Naloxone | 1.03 ± 0.1 | 0.67 ± 0.07 | 1761.87 ± 236.85 | 9.10 ± 1.22 | 8.94 ± 2.08 |
| Naloxegol | 0.79 ± 0.02 [* a] | 0.88 ± 0.02 [a] | 3682.32 ± 565.51 [a] | 4.37 ± 0.71 [* a] | 5.58 ± 0.96 |
| AG10-L2-Nal | 0.20 ± 0.04 [*] | 3.62 ± 0.76 [*] | 17576.12 ± 2537.87 [*] | 0.92 ± 0.14 [*] | 4.89 ± 1.83 |

**Supplementary Table 5.** Pharmacokinetic parameters of oxycodone and AG10-L2-Oxy determined from the plasma concentrations after the subcutaneous dosing. Statistical differences were determined using two tailed unpaired t-test with equal variance. For k, T(6) = 12.33, $P < 0.0001$; for $t_{1/2}$, T(6) = 14.78, $P < 0.0001$; for $AUC_{inf}$, T(6) = 13.21, $P < 0.0001$; for CL/F, T(6) = 11.36, $P < 0.0001$; for $V_{ss}/F$, T(6) = 0.7609, $P = 0.4756$ (* represents $P < 0.05$ compared to oxycodone group). All data are presented as mean (± s.d.) (n = 4 rats per group). Source data are provided as a Source Data file in Figure 8a source data.

| | k (1/h) | $t_{1/2}$ (h) | $AUC_{inf}$ (nM*h) | CL/F (L/h/kg) | $V_{ss}/F$ (L/kg) |
|---|---|---|---|---|---|
| Oxycodone | 0.9 ± 0.12 | 0.78 ± 0.09 | 4852.00 ± 676.31 | 3.32 ± 0.47 | 3.76 ± 0.85 |
| AG10-L2-Oxy | 0.15 ± 0.02 * | 4.68 ± 0.52 * | 25755.00 ± 3090.4 * | 0.62 ± 0.07 * | 4.22 ± 0.86 |

# Supplementary Methods

**Materials and reagents.** Naloxone hydrochloride (#N285000) and naloxegol oxalate (#N284475) were purchased from Toronto Research Chemicals, Toronto, Ontario. Morphine sulfate (CII), USP (#M1167), Oxycodone (#O1169) and methylnaltrexone bromide (#HY-75766) were purchased from Spectrum Chemical Manufacturing Corporation and MedChemExpress (MCE) LLC, USA, respectively. Activated carbon powder (USP) (#C272-500) that was used in the GI transit study was bought from Fisher Scientific. Gum Arabic powder was bought from ACROS Organics™ (#AC258852500). Hot plate analgesia meter with start/stop foot switch control was purchased from Columbus Instruments, Columbus, OH, USA. Rabbit anti-RBP4 antibody was purchased from Abcam (#ab154914). IRdye800 donkey antirabbit secondary antibody was bought from LI-COR Biosciences (#926-32213). Prealbumin from human plasma (human TTR) (#P1742) and human serum (#H4522) were purchased from Sigma. Glutaraldehyde was bought from Sigma (#G5882). Mouse Balb C Serum (#IMSBCSER) and Innovative grade US origin rat Sprague Dawley Complement Serum (#IGRTSDCSER) were purchased from Innovative Research. RPMI-1640 Medium (HyClone, Utah, USA), fetal bovine serum (Gemini), penicillin/streptomycin (100 unit/mL and 100 µg/mL (Gibco, NY, USA) and L-Glutamine (Glutamax-100X, #35-050-061, Gibco, NY, USA) were bought from the companies mentioned in the parentheses. CellBIND® 96-well clear plates (#CLS3340-50EA) were purchased from Corning®. CellTiter 96 non-radioactive cell proliferation assay kit (#G4000, Promega, WI, USA) was used to perform the MTT (3-(4, 5-dimethylthiazol- 2-yl)-2, 5 diphenyltetrazolium bromide) assay for the test compounds in both the cell lines.

**Chemical synthesis and HPLC purity analysis**

**Chemistry general procedures.** All reactions were carried out under an argon or nitrogen atmosphere using dry solvents under anhydrous conditions, unless otherwise noted. The solvents used were ACS grade from Fisher. Yields refer to chromatographically and spectroscopically ($^{1}$H NMR and $^{13}$C NMR) homogeneous materials, unless otherwise noted. Reagents were purchased from Aldrich and Fisher and used without further purification. N-Boc-2-azidoethylamine (#DA-458) was purchased from Acrotein ChemBio Inc., Hoover, AL, USA. Reactions were monitored

by thin-layer chromatography (TLC) carried out on EMD Millipore® silica gel 60 plastic TLC plates coated with fluorescent indicator $F_{254}$ TLC (#1.05735.0001), using UV light and iodine chamber as visualizing agents. Normal phase flash column chromatography was carried out using Combi Flash® Rf+ Lumen instrument (Teledyne ISCO) with High Performance Silica Flash Column (RediSep® Rf+ Gold), and preparative thin-layer chromatography (PTLC) separations were carried out on Analtech® 2mm (60F-254) (#P02015). CombiFlash® Rf+ Lumen version 2.1.33 software was used to analyze the purification process of flash column chromatography of the synthesized molecules. $^{1}$H NMR and $^{13}$C NMR spectra were recorded on a JEOL JNM−ECA 600 spectrometer and calibrated using residual undeuterated solvent as an internal reference. $^{1}$H NMR and $^{13}$C NMR spectra were analyzed using Delta v5.3 NMR Processing and Control software. High-resolution mass spectra (HRMS) were determined by JEOL AccuTOF DART using helium as an ionization gas and polyethylene glycol (PEG) as an external calibrating agent. Coupling constants (J) were expressed in Hertz.

**Preparative HPLC method for purification of the synthesized compounds.** The purification was performed on a Waters Delta 600 HPLC system connected to a photodiode array detector operating between the UV ranges of 210 – 600 nm, using Waters Masslynx V4.1 software. The HPLC analysis was performed on an XBridge® Prep C18 Column (10x250 mm, 5 µm) at ambient temperature upon injection of 5 mL of each sample to obtain the chromatogram at 254 nm UV absorbance. The mobile phase was composed of solvent A consisting of acetonitrile-water (5:95, v/v) containing 0.1% trifluoroacetic acid and solvent B consisting of acetonitrile-water (95:5, v/v) containing 0.1% trifluoroacetic acid and delivered at a flow of 2.0 mL/minute. The HPLC method was a gradient separation increasing linearly from 0-100% solvent B.

**Analytical HPLC method for evaluating the purity of the synthesized compounds.** Detailed HPLC information of key compounds (traces, retention times, and % purity) are included below. The analysis of the key compounds' purity (>95% for all compounds) was performed using C18 and C4 reverse-phase HPLC columns on Waters e2695 separations module HPLC system connected to a Waters 2998 photodiode array detector operating between the UV ranges of 200-400 nm and quantified using the Empower 3 software. The HPLC analysis was performed on a Waters™ XBridge C18 column (4.6x250 mm, 5µm) or a Waters™ Symmetry300 C4 column

(2.1x150 mm, 5 µm), eluting at 0.5 mL/minute, at ambient temperature upon injection of 50 µL of each sample to obtain the chromatogram at 254 nm UV absorbance. The mobile phase was composed of solvent A consisting of acetonitrile-water (5:95, v/v) containing 0.1% trifluoroacetic acid and solvent Bconsisting of acetonitrile-water (95:5, v/v) containing 0.1% trifluoroacetic acid. The HPLC program for the C4 and C18 column was a gradient method increasing linearly from 0-100 % solvent B at 0-20 minutes, followed by isocratic elution at 100% solvent B until 22 minutes, going back down to 0% of B at 24 minutes, and then remaining at 0% of B for 1 minute.

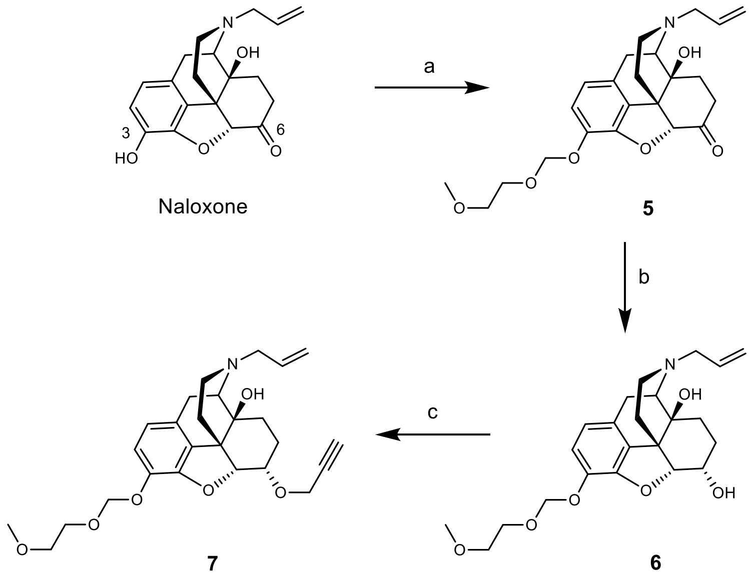


**Supplementary Figure 16.** Synthesis of naloxone derivative **7**. **(a)** diisopropylethylamine, 2-methoxyethoxymethyl chloride, $CH_2Cl_2$, room temperature, overnight; **(b)** sodium triethylborohydride, THF, 0°C, 3.5 hours; **(c)** sodium hydride, DMF, propargyl bromide, 4 hours.

**(4*R*,4a*S*,7*S*,7a*R*,12b*S*)-3-allyl-9-((2-methoxyethoxy)methoxy)-1,2,3,4,5,6,7,7a-octahydro-4a*H*-4,12-methanobenzofuro[3,2-e]isoquinoline-4a,7-diol (α-6-OH-3-MEM-O-Naloxol) (6).**

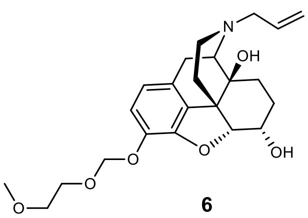

**6**

Compound **5** was synthesized as reported earlier (Patent US 8,563,726 B2). To a solution of **5** (3-MEM-O-naloxone base) (100 mg, 0.24 mmol, 1 equiv) in dry tetrahydrofuran (6 mL) under an inert atmosphere at 0°C, was added 1M solution of sodium triethylborohydride (0.36 mL, 0.36

mmol, 1.5 equiv) slowly. The solution was stirred under a nitrogen atmosphere for 5 hours and warmed to room temperature. Acetic acid (0.5 mL) was then added slowly to destroy excess sodium triethylborohydride. The solvent was removed under reduced pressure and the remaining residue was dissolved in $CH_2Cl_2$ (30 mL). The $CH_2Cl_2$ phase was extracted with 0.1 N HCl/NaCl water solution (3x30 mL) and the combined aqueous extracts were washed with $CH_2Cl_2$ (1x30 mL). Sodium carbonate was added to bring the aqueous solution to pH = 8. The solution was extracted once again with $CH_2Cl_2$ (3x30 mL) and the organic extracts were combined, dried over anhydrous $Na_2SO_4$, filtered, and the solvent was removed under reduced pressure, and the resulting residue was dried overnight in vacuo to afford compound **6** (89 mg, 0.21 mmol, 89% isolated yield) as a colorless viscous liquid. $^1$H-NMR (600 MHz, $CDCl_3$) δ 6.81 (d, 1H, *J* = 8.2 Hz), 6.56 (d, 1H, *J* = 8.2 Hz), 5.81-5.74 (m, 1H), 5.53 (d, 1H, *J* = 6.9Hz), 5.19-5.10 (m, 3H), 4.58 (d, 1H, *J* = 5.5 Hz) 4.16-4.12 (m, 1H), 3.91-3.88 (m, 1H), 3.79-3.76 (m, 1H), 3.52-3.50 (m, 2H), 3.33 (s, 3H), 3.09-2.88 (m, 5H), 2.60-2.51 (m, 2H), 2.22-2.16 (m, 2H), 1.85-1.80 (m, 1H), 1.53-1.46 (m, 3H), 1.31-1.25 (m, 1H); $^{13}$C-NMR (150 MHz, $CDCl_3$) δ 147.54, 138.03, 135.56, 132.59, 127.66, 119.73, 119.37, 118.05, 95.22, 90.76, 71.71, 70.27, 68.15, 66.90, 62.69, 59.20, 58.08, 46.58, 43.37, 32.77, 27.56, 24.16, 23.18. $^1$H NMR ($CDCl_3$) showed that the desired product was greater than 99% α-6-OH-3-MEM-O-naloxol, no β epimer was detected. ESI-MS: *m/z* calcd for $C_{23}H_{31}NO_6$ $[M+H]^+$ 418.2, $[M+Na]^+$ 440.2; found: 417.8, 440.3.

**(4R,4aS,7S,7aR,12bS)-3-allyl-9-((2-methoxyethoxy)methoxy)-7-(prop-2-yn-1-yloxy)-1,2,3,4,5,6,7,7a-octahydro-4aH-4,12-methanobenzofuro[3,2-e]isoquinolin-4a-ol (7).**

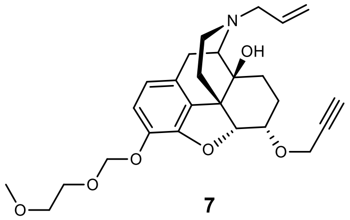


**7**

To a solution of α-6-OH-3-MEM-O-Naloxol (**6**) (89 mg, 0.21 mmol, 1 equiv) in anhydrous dimethylformamide (2 mL) under an inert atmosphere at 0 °C (via an ice bath), was added sodium hydride (60% in oil) (12.79 mg, 0.32 mmol, 1.5 equiv) slowly. The solution was stirred under a nitrogen atmosphere for 1 hour. Then propargyl bromide (80 % w/v) (47.54 µL, 0.32 mmol, 1.5 equiv) was added dropwise to the solution. After 4 hours, 0.5 mL deionized water was added to the reaction mixture to quench the reaction. Then 50 mL ethyl acetate was added to the solution

and extracted with brine (4x25 mL) and the combined aqueous extracts were washed again with ethyl acetate (1x25 mL). The combined ethyl acetate fractions were dried over anhydrous $Na_2SO_4$, filtered, and the solvent was removed under reduced pressure. The residue was purified by flash column chromatography (silica gel, 0-100% ethyl acetate/hexane) to afford compound **7** (73 mg, 0.16 mmol, 75% isolated yield) as a brownish viscous liquid. $^1$H-NMR (600 MHz, $CDCl_3$) δ 6.91 (d, 1H, *J* = 8.2 Hz), 6.53 (d, 1H, *J* = 8.2 Hz), 5.80-5.74 (m, 1H), 5.27 (d, 1H, *J* = 6.9 Hz), 5.22 (d, 3H, *J* = 6.9 Hz), 4.70 (d, 1H, *J* = 4.1 Hz), 4.28-4.21 (m, 2H), 4.14-4.11 (m, 1H), 3.89-3.82 (m, 2H), 3.54 (t, 2H, *J* = 4.5 Hz), 3.35 (s, 3H), 3.09-3.03 (m, 3H), 2.88 (d, 1H, *J* = 5.5 Hz), 2.60-2.50 (m, 2H), 2.37 (t, 1H, *J* = 2.4 Hz), 2.23-2.15 (m, 2H), 1.77-1.73 (m, 1H), 1.61-1.41 (m, 3H), 1.30-1.23 (m, 1H); $^{13}$C-NMR (150 MHz, $CDCl_3$) 148.53, 138.97, 135.48, 131.57, 127.61, 118.68, 118.09, 95.30, 89.00, 80.42, 74.30, 73.33, 71.85, 70.26, 67.98, 62.59, 59.19, 58.12, 56.81, 47.35, 43.22, 33.29, 28.35, 23.19, 21.16. ESI-MS: *m/z* calcd for $C_{26}H_{33}NO_6$ $[M+H]^+$ 456.2, $[M+Na]^+$ 478.2; found: 456.6, 478.6.

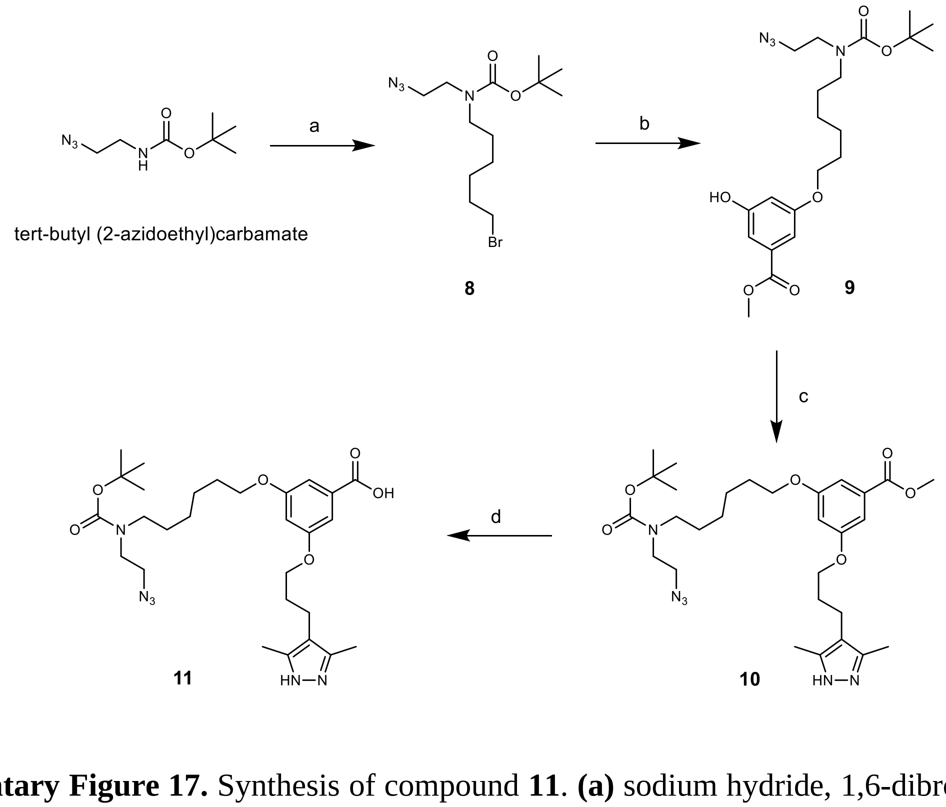


**Supplementary Figure 17.** Synthesis of compound **11**. **(a)** sodium hydride, 1,6-dibromohexane, DMF, room temperature, 2 hours; **(b)** methyl 3,5-dihydroxybenzoate, $K_2CO_3$, DMF, room temperature, 16 hours; **(c)** triphenylphosphine, 3-(3,5-dimethyl-1H-pyrazol-4-yl)propan-1-ol, diisopropyl azodicarboxylate, THF, ultrasonication, 15 minutes; **(d)** $LiOH.H_2O$, $H_2O$/THF (1:1), room temperature, 14 hours.

**tert-butyl (2-azidoethyl)(6-bromohexyl)carbamate (8).**

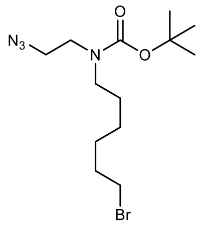


**8**

To a solution of tert-butyl (2-azidoethyl)carbamate (6000 mg, 32.24 mmol, 1 equiv) in dry dimethylformamide (50 mL) under an inert atmosphere, was added sodium hydride (60% in oil) (2579 mg, 64.48 mmol, 2 equiv). The solution was stirred under a nitrogen atmosphere for 1 hour. Then 1,6-dibromohexane (density = 1.58 g/mL) (24.68 mL, 161.1 mmol, 5 equiv) was added all at one time to the solution. After 2 hours, 5 mL deionized water was added to the reaction mixture slowly under stirring to quench the reaction. Then 400 mL ethyl acetate was added to the solution and was extracted with brine (3x150 mL) and the combined aqueous extracts were washed again with ethyl acetate (1x150 mL). The combined ethyl acetate fractions were dried over anhydrous $Na_2SO_4$, filtered, and the solvent was removed under reduced pressure. The residue was purified by flash column chromatography (silica gel, 0-100% ethyl acetate/hexane) to afford compound **8** (3890 mg, 11.14 mmol, 35% isolated yield). $^1$H NMR ($CD_3OD$, 600 MHz) δ 3.46-3.41 (m, 6H), 3.26 (t, 2H, *J* = 7.0 Hz), 1.88-1.84 (m, 2H), 1.59-1.56 (m, 2H), 1.51-1.47 (m, 11H), 1.37-1.30 (m, 2H); $^{13}$C-NMR (150 MHz, $CD_3OD$) δ 157.47, 81.44, 51.13, 50.62, 47.77, 34.40, 34.03, 29.62, 29.04, 28.85, 27.09**.** ESI-MS: *m/z* calcd for $C_{13}H_{25}BrN_4O_6$ $[M+Na]^+$ 371.1; found: 371.1.

**methyl 3-((6-((2-azidoethyl)(tert-butoxycarbonyl)amino)hexyl)oxy)-5-hydroxybenzoate (9).**

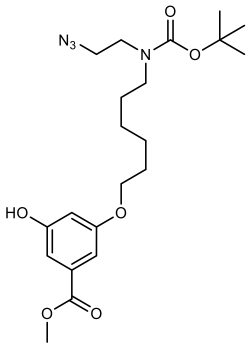

**9**

To a solution of **8** (3890 mg, 11.14 mmol, 1 equiv) and methyl 3,5-dihydroxybenzoate (5620 mg, 33.42 mmol, 3 equiv) in anhydrous dimethylformamide (30 mL), was added $K_2CO_3$ (2309 mg, 16.71 mmol, 1.5 equiv). The suspension was stirred at room temperature for 16 hours. The suspension was quenched with water, diluted with ethyl acetate (300 mL), and washed with brine (3x150 mL). The ethyl acetate fraction was dried over anhydrous sodium sulfate and concentrated in vacuo. The solution was concentrated under reduced pressure, and the residue was purified by flash column chromatography (silica gel, 0-20% ethyl acetate/hexane) to afford **9** (2.86 g, 59% yield); $^1$H NMR ($CD_3OD$, 600 MHz) δ 7.02 (d, 2H, *J* = 2.3 Hz), 6.56 (t, 1H, *J* = 2.3 Hz), 3.96 (t, 2H, *J* = 6.3 Hz), 3.86 (s, 3H), 3.41-3.38 (m, 4H), 3.26 (t, 2H, *J* = 7.3 Hz), 1.80-1.75 (m, 2H), 1.59-1.48 (m, 4H), 1.46 (s, 9H), 1.39-1.34 (m, 2H); $^{13}$C-NMR (150 MHz, $CD_3OD$) δ 167.2, 160.4, 158.5, 156.1, 131.7, 108.6, 106.4, 106.2, 80.0, 67.7, 51.3, 49.6, 49.1, 46.3, 28.9, 28.2, 27.4, 26.2, 25.5**.** ESI-MS: *m/z* calcd for $C_{21}H_{32}N_4O_6$ $[M+Na]^+$ 459.2; found: 459.5.

**3-((6-((2-azidoethyl)(tert-butoxycarbonyl)amino)hexyl)oxy)-5-(3-(3,5-dimethyl-1H-pyrazol-4-yl)propoxy)benzoic acid (11).**

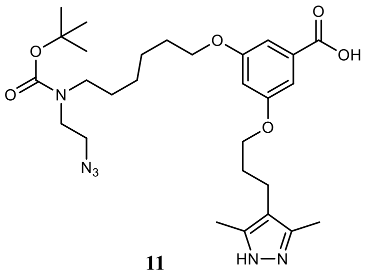

**11**

A mixture of **9** (1000 mg, 2.29 mmol, 1 equiv), 3-(3,5-dimethyl-1H-pyrazol-4-yl)propan-1-ol (371 mg, 2.41 mmol, 1.05 equiv), and triphenylphosphine (750 mg, 2.86 mmol, 1.25 equiv) in THF (6 mL) was sonicated at 42 KHz for 2 minutes[1]. Then, diisopropyl azodicarboxylate (616 µL, 2.86 mmol, 1.25 equiv) (94% w/v) was added dropwise over the course of 2 minutes and sonicated for 15 minutes. The solution was concentrated under reduced pressure. The residue was purified by flash column chromatography (silica gel, 0-10% MeOH/$CH_2Cl_2$) to afford intermediate **10** (941 mg, 72% yield). To a solution of **10** (1915 mg, 3.33 mmol, 1 equiv) in a mixture of THF (8 mL) and water (8 mL) was added $LiOH.H_2O$ (280 mg, 6.67 mmol, 2 equiv). The reaction mixture was heated at 50 °C for 14 hours and then concentrated under reduced pressure. Acetic acid was added to bring the aqueous solution to pH 4 and the solution was concentrated under reduced pressure. The residue was extracted by 10% methanol in ethyl acetate. The combined organic extracts were concentrated under reduced pressure to afford **11** (1.09 g, 59% yield); $^1$H NMR ($CD_3OD$, 600 MHz) δ 7.14-7.12 (m, 2H), 6.66 (t, 1H, *J* = 2.4 Hz), 3.99 (t, 2H, *J* = 6.2 Hz), 3.92 (t, 2H, *J* = 5.8 Hz), 3.43-3.38 (m, 4H), 3.26 (t, 2H, *J* = 7.2 Hz), 2.58 (t, 2H, *J* = 7.2 Hz), 2.14 (s, 6H), 1.94-1.90 (m, 2H), 1.81-1.77 (m, 2H), 1.59-1.50 (m, 4H), 1.46 (s, 9H), 1.39-1.34 (m, 2H); $^{13}$C-NMR (150 MHz, $CD_3OD$) δ 170.10, 161.79, 161.65, 143.43, 134.29, 115.41, 108.98, 107.18, 81.46, 69.30, 67.97, 51.12, 50.62, 47.75, 30.94, 30.35, 29.72 , 28.85, 27.66, 27.03, 19.99, 10.71. ESI-MS: *m/z* calcd for $C_{28}H_{42}N_6O_6$ $[M+H]^+$ 559.3; found: 559.5.

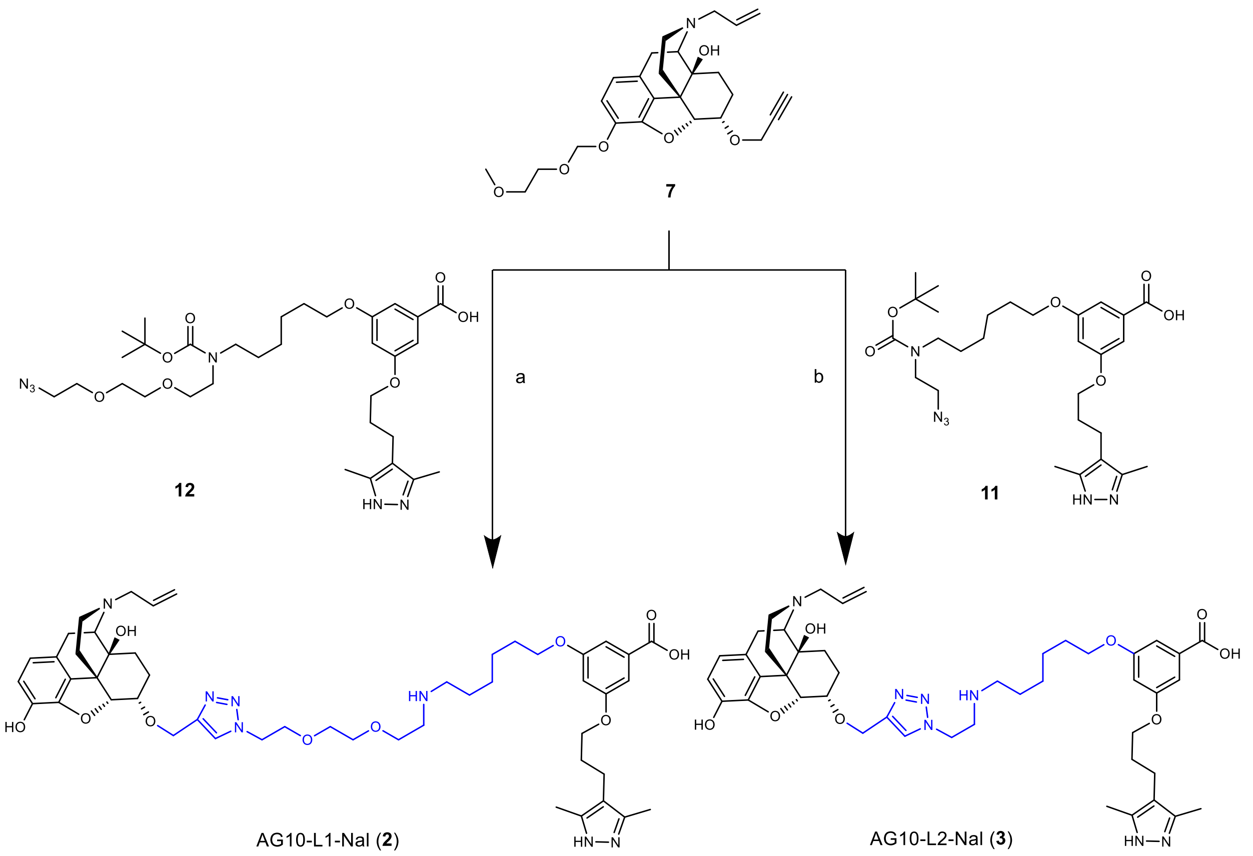


**Supplementary Figure 18.** Synthesis of AG10-L1-Nal (**2**) and AG10-L2-Nal (**3**). **(a)** (i) Compound **11**, $CuSO_4$, sodium ascorbate, THF/$H_2O$ (4:1), room temperature, overnight; (ii) $CH_2Cl_2$/TFA (4:1), room temperature, 2 hours; **(b)** (i) Compound **11**, $CuSO_4$, sodium ascorbate, THF/$H_2O$ (4:1), room temperature, overnight; (ii) $CH_2Cl_2$/TFA (4:1), room temperature, 2 hours.

**3-((6-((2-(2-(2-(4-((((4R,7S,7aR,12bS)-3-allyl-4a,9-dihydroxy-2,3,4,4a,5,6,7,7a-octahydro-1H-4,12-methanobenzofuro[3,2-e]isoquinolin-7-yl)oxy)methyl)-1H-1,2,3-triazol-1-yl)ethoxy)ethoxy)ethyl)amino)hexyl)oxy)-5-(3-(3,5-dimethyl-1H-pyrazol-4-yl)propoxy)benzoic acid (AG10-L1-Nal) (2).**

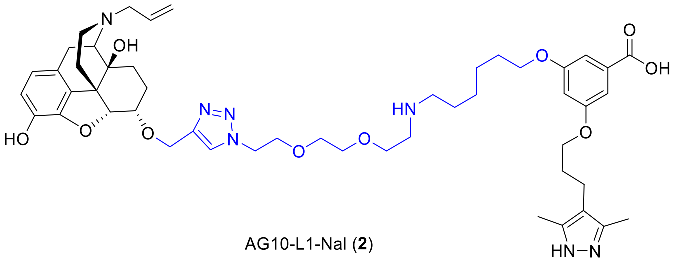

AG10-L1-Nal (**2**)

The click (CuAAC) coupling was carried out by **7** (21 mg, 0.0461 mmol, 1 equiv), **12** (synthesized as reported earlier[2] (29.8 mg, 0.0461 mmol, 1 equiv), $CuSO_4.5H_2O$ (2.9 mg, 0.0115 mmol, 0.25 equiv), and sodium ascorbate (4.6 mg, 0.0231 mmol, 0.5 equiv) in a) 3 mL mixture of THF/$H_2O$ (4:1. The reaction mixture was stirred at room temperature overnight. The solution was concentrated under reduced pressure. The residue was extracted by hexane:ethyl acetate. The combined organic extracts were concentrated under reduced pressure to afford an intermediate and was used for the next step directly. Then, to a solution of the intermediate, was added a mixture containing TFA and $CH_2Cl_2$, (1:4 ratio) (1 mL), and the reaction mixture was stirred at room temperature for 2 hours. The solution was concentrated under reduced pressure and purified by preparative HPLC to afford compound AG10-L1-Nal (**2**) (18 mg, 43% yield over two steps) (98% purity by HPLC); $t_R$ (C4 column) = 10.0 minutes; $t_R$ (C18 column) = 19.2 minutes; $^1$H NMR ($CD_3OD$, 600 MHz) δ 7.90 (s, 1H), 7.15-7.13 (m, 2H), 6.74-6.69 (m, 2H), 6.65 (d, 1H, *J* = 8.2 Hz), 5.97-5.92 (m, 1H), 5.68-5.61 (m, 2H), 4.85 (d, 1H, *J* = 5.5 Hz), 4.75 (d, 1H, J = 11.7 Hz), 4.66 (d, 1H, *J* = 11.7 Hz), 4.59-4.57 (m, 2H), 4.13-4.10 (m, 1H), 4.01-3.98 (m, 4H), 3.93-3.89 (m, 3H), 3.81-3.78 (m, 1H), 3.66-3.59 (m, 8H), 3.20-3.17 (m, 3H), 3.06-3.00 (m, 2H), 2.90-2.85 (m, 1H), 2.72 (t, 2H, J = 7.2 Hz), 2.65-2.60 (m, 1H), 2.34 (s, 6H), 2.03-1.99 (m, 2H), 1.93-1.88 (m, 1H), 1.84-1.79 (m, 2H), 1.75-1.55 (m, 8H), 1.50-1.45 (m, 2H), 1.44-1.38 (m, 1H); $^{13}$C-NMR (150 MHz, $CD_3OD$) δ 169.61, 162.69, 162.45, 161.83, 161.47, 147.44, 146.43, 144.96, 140.69, 134.09, 130.64, 128.07, 126.68, 125.70, 122.36, 120.43, 119.63, 118.76, 109.16, 108.90, 107.28, 88.98, 75.41, 71.64, 71.46, 71.40, 70.43, 69.24, 67.85, 67.04, 64.45, 57.02, 51.47, 48.60, 47.75, 47.05, 31.02, 30.15, 30.04, 28.56, 27.42, 27.16, 26.79, 24.41, 22.10, 19.49, 9.82; HRMS (DART) *m/z:* calcd for $C_{49}H_{68}N_7O_{10}$ $[M+H]^+$ 914.5022; found: 914.5039.

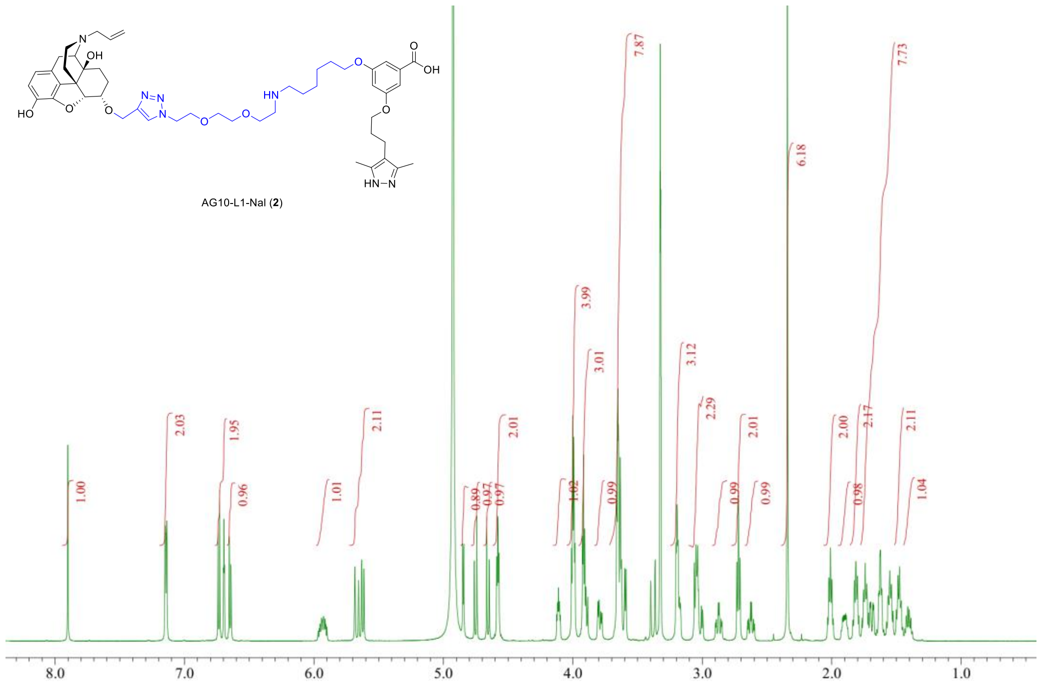


**Supplementary Figure 19.** $^{1}$H NMR Spectrum for AG10-L1-Nal (**2**).

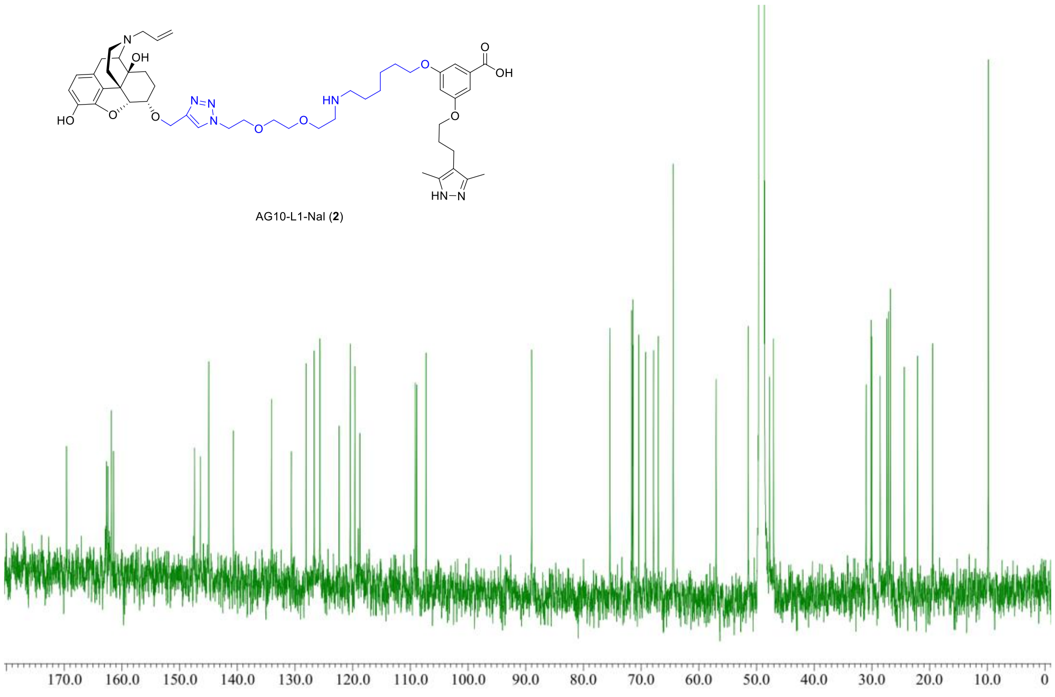


**Supplementary Figure 20.** $^{13}$C NMR Spectrum for AG10-L1-Nal (**2**).

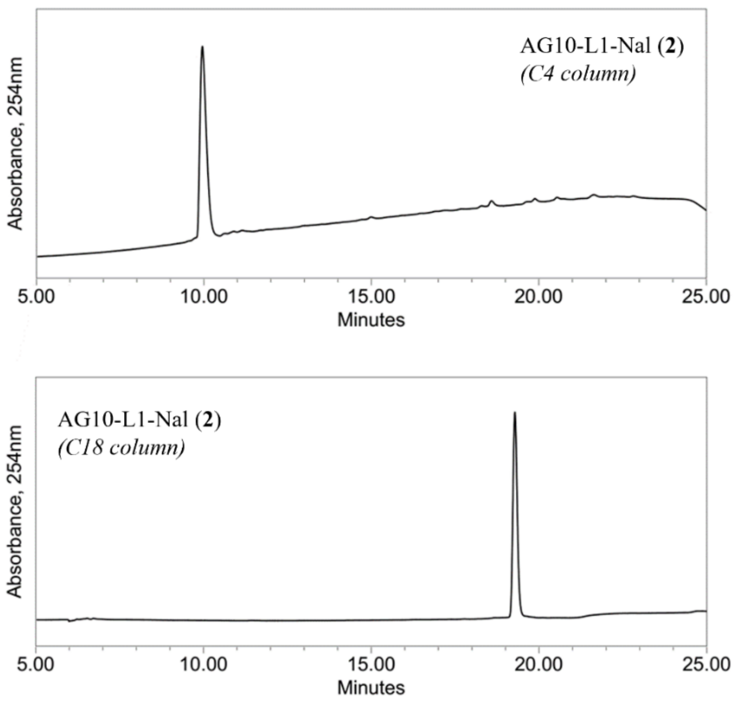


**Supplementary Figure 21.** HPLC traces of AG10-L1-Nal (**2**).

**3-((6-((2-(4-((((4R,7S,7aR,12bS)-3-allyl-4a,9-dihydroxy-2,3,4,4a,5,6,7,7a-octahydro-1H-4,12-methanobenzofuro[3,2-e]isoquinolin-7-yl)oxy)methyl)-1H-1,2,3-triazol-1-yl)ethyl)amino)hexyl)oxy)-5-(3-(3,5-dimethyl-1H-pyrazol-4-yl)propoxy)benzoic acid (AG10-L2-Nal) (3).**

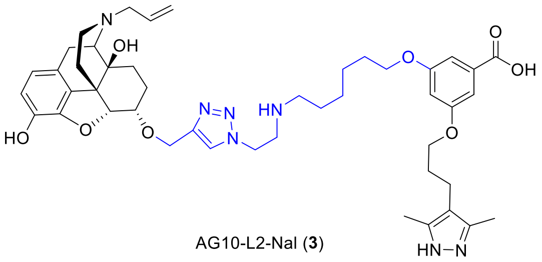

AG10-L2-Nal (**3**)

The click (CuAAC) coupling was carried out by **7** (260 mg, 0.571 mmol, 1 equiv), **11** (319 mg, 0.571 mmol, 1 equiv), $CuSO_4.5H_2O$ (37 mg, 0.148 mmol, 0.25 equiv), and sodium ascorbate (57 mg, 0.286 mmol, 0.5 equiv) in a 5 mL mixture of THF/$H_2O$ (4:1). The reaction mixture was stirred at room temperature overnight. The solution was concentrated under reduced pressure. The residue was extracted by hexane:ethyl acetate. The combined organic extracts were concentrated under reduced pressure to afford an intermediate and was used for the next step directly. Then, to a solution of the intermediate, was added a mixture containing TFA and $CH_2Cl_2$, (1:4 ratio) (1 mL), and the reaction mixture was stirred at room temperature for 2 hours. The solution was concentrated under reduced pressure and purified by preparative-HPLC to afford compound AG10-L2-Nal (**3**) (216 mg, 46% yield over two steps) (98% purity by HPLC); $t_R$ (column) (C4) = 7.0 minutes; $t_R$ (column) (C18) = 14.6 minutes; $^1$H NMR ($CD_3OD$, 600 MHz) δ 7.89 (s, 1H), 7.14-7.12 (m, 2H), 6.71 (d, 1H, *J* = 8.2 Hz), 6.68 (t, 1H, *J* = 2.4 Hz), 6.63 (d, 1H, *J* = 8.2 Hz), 5.96-5.89 (m, 1H), 5.67-5.60 (m, 2H), 4.83 (d, 1H, *J* = 5.5 Hz), 4.78-4.75 (m, 3H), 4.64 (d, 1H, *J* = 12.4 Hz), 4.08-4.06 (m, 1H), 4.01-3.97 (m, 4H), 3.91-3.87 (m, 1H), 3.79-3.76 (m, 1H), 3.62-3.57 (m, 3H), 3.19-3.16 (m, 1H), 3.09-3.07 (m, 2H), 3.03-2.98 (m, 1H), 2.87-2.82 (m, 1H), 2.71 (t, 2H, *J* = 7.2 Hz), 2.63-2.58 (m, 1H), 2.33 (s, 6H), 2.03-1.97 (m, 2H), 1.94-1.71 (m, 6H), 1.68-1.61 (m, 2H), 1.58-1.52 (m, 3H), 1.50-1.45 (m, 3H); $^{13}$C-NMR (150 MHz, $CD_3OD$) δ 169.62, 147.39, 147.24, 145.01, 140.75, 134.06, 130.75, 128.09, 126.62, 125.75, 122.09, 120.31, 119.54, 109.16, 108.91, 107.25, 88.84, 75.52, 71.47, 69.21, 67.81, 64.64, 64.58, 56.98, 47.93, 47.91, 47.40, 46.83, 30.73, 30.12, 29.99, 27.97, 27.35, 27.13, 26.75, 24.36, 22.56, 19.48, 9.81; HRMS (DART) *m/z:* calcd for $C_{45}H_{60}N_7O_8$ $[M+H]^+$ 826.4498; found: 826.4490.

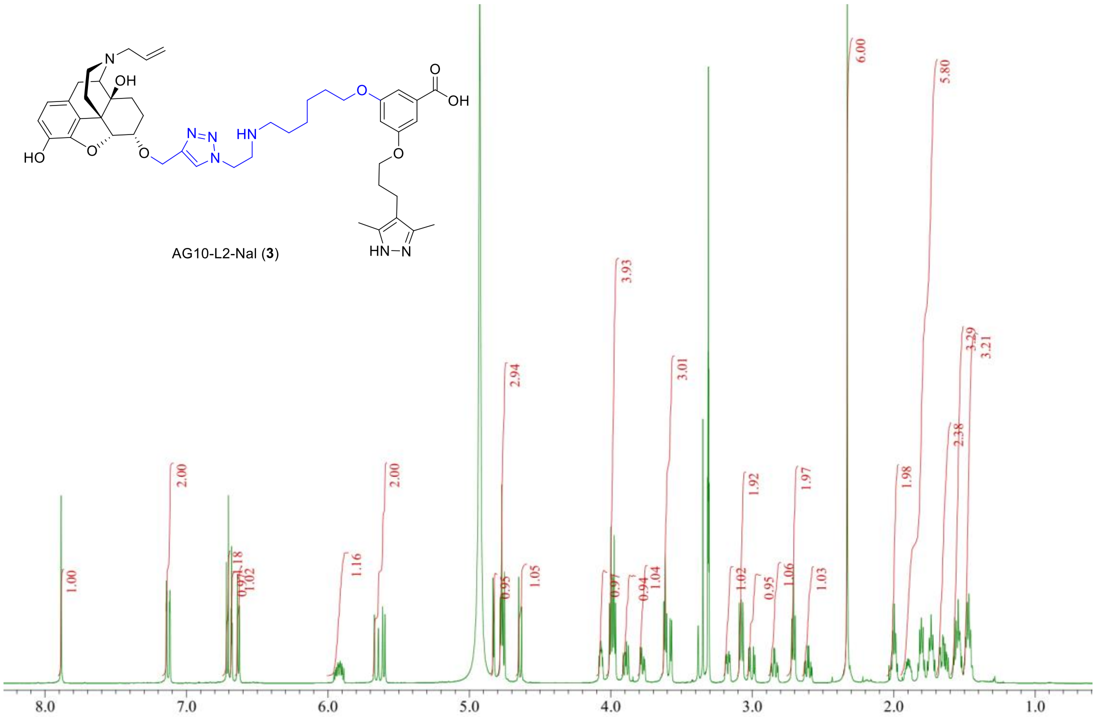


**Supplementary Figure 22.** $^{1}H$ NMR Spectrum for AG10-L2-Nal (**3**).

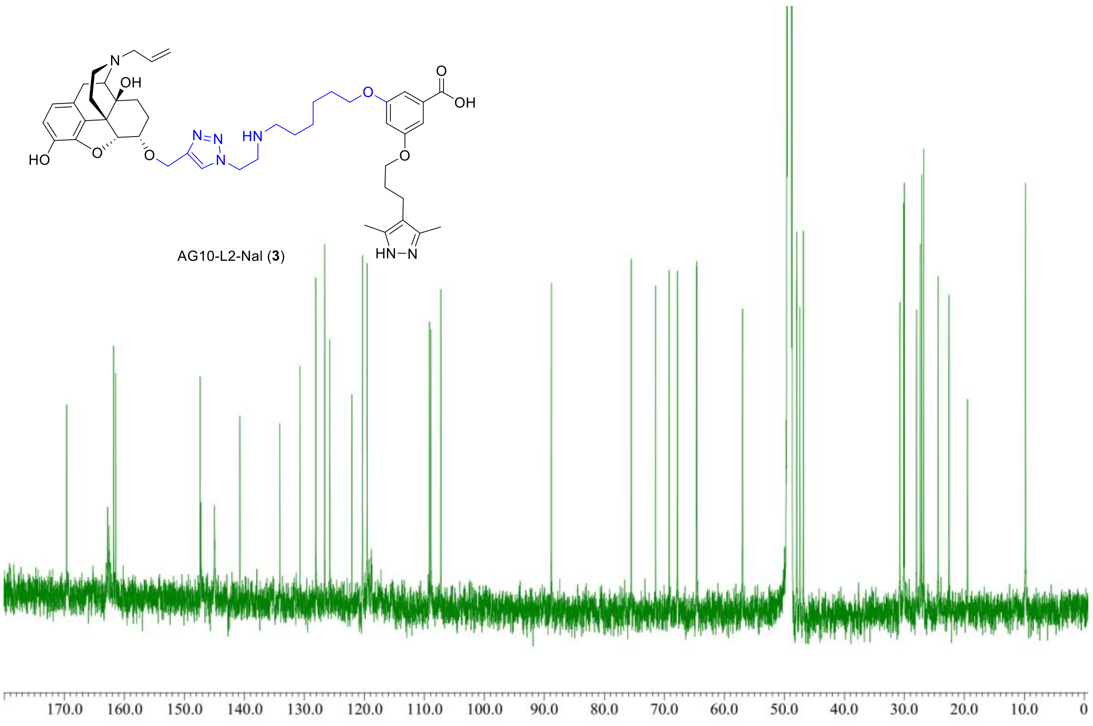


**Supplementary Figure 23.** $^{13}C$ NMR Spectrum for AG10-L2-Nal (**3**).

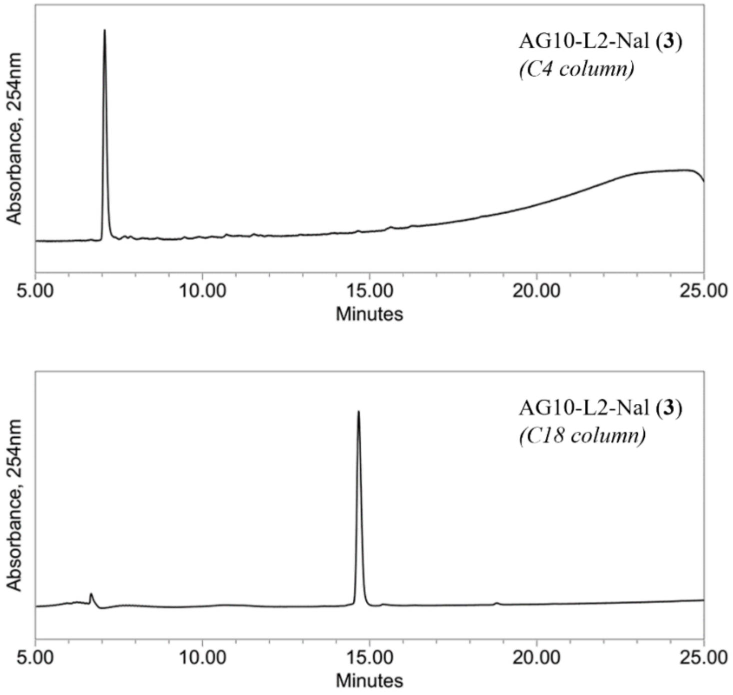


**Supplementary Figure 24.** HPLC traces of AG10-L2-Nal (**3**).

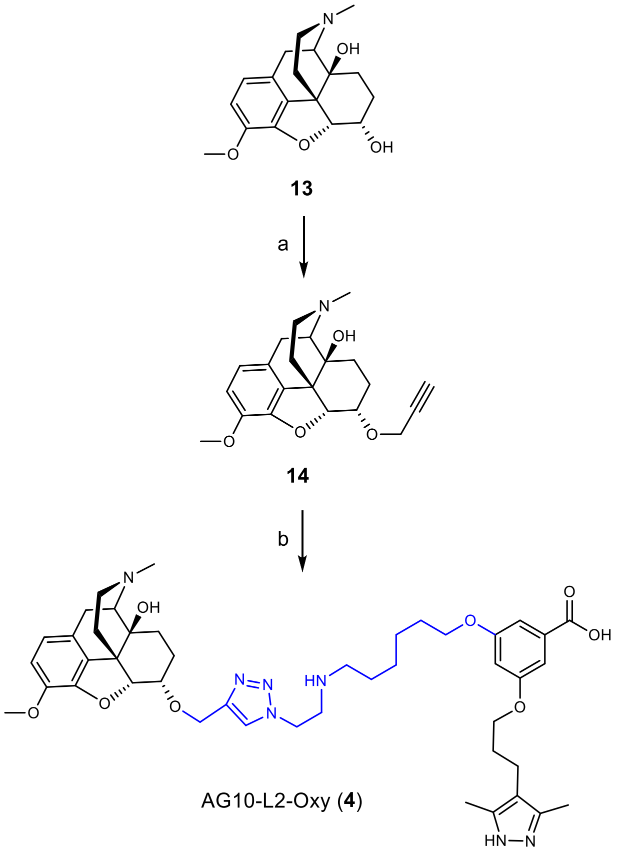


**Supplementary Figure 25.** Synthesis of AG10-L2-Oxy (**4**). **(a)** Sodium hydride, DMF, propargyl bromide, 4 hours; **(b)** (i) Compound **11**, $CuSO_4$, sodium ascorbate, THF/$H_2O$ (4:1), room temperature, overnight; (ii) $CH_2Cl_2$/TFA (4:1), room temperature, 2 hours.

**(4R,4aS,7S,7aR,12bS)-9-methoxy-3-methyl-7-(prop-2-yn-1-yloxy)-1,2,3,4,5,6,7,7a-octahydro-4aH-4,12-methanobenzofuro[3,2-e]isoquinolin-4a-ol (14).**

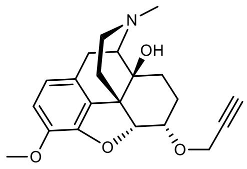

**14**

The synthesis procedure is similar to compound **7.** To a solution of (4R,4aS,7S,7aR,12bS)-9-methoxy-3-methyl-1,2,3,4,5,6,7,7a-octahydro-4aH-4,12-methanobenzofuro[3,2-e]isoquinoline-4a,7-diol **(13)** (synthesized as reported earlier) (Patent US 8,575,196 B2) (400.0 mg, 1.26 mmol,

1 equiv) in anhydrous dimethylformamide (15 mL) under an inert atmosphere at 0 °C (via an ice bath), was added sodium hydride (60% in oil) (201.6 mg, 5.04 mmol, 4 equiv) slowly. The solution was stirred under a nitrogen atmosphere for 1 hour. Then propargyl bromide (80 % w/v) (243.6 µL, 1.64 mmol, 1.3 equiv) was added dropwise to the solution. Reaction mixture was stirred at 60 °C overnight. Then, 5 mL deionized water was added to the reaction mixture to quench the reaction. Then 100 mL ethyl acetate was added to the solution and extracted with brine (3x50 mL) and the combined aqueous extracts were washed again with ethyl acetate (2x50 mL). The combined ethyl acetate fractions were dried over anhydrous $Na_2SO_4$, filtered, and the solvent was removed under reduced pressure. The residue was purified by flash column chromatography (silica gel, 0-10% dichloromethane/methanol) to afford compound **14** (259.7 mg, 0.73 mmol, 58% yield). ESI-MS: *m/z* calcd for $C_{21}H_{25}NO_4$ $[M+H]^+$ 356.2; found: 356.1.

**3-(3-(3,5-dimethyl-1H-pyrazol-4-yl)propoxy)-5-((6-((2-(4-((((4R,4aS,7S,7aR,12bS)-4a-hydroxy-9-methoxy-3-methyl-2,3,4,4a,5,6,7,7a-octahydro-1H-4,12-methanobenzofuro[3,2-e]isoquinolin-7-yl)oxy)methyl)-1H-1,2,3-triazol-1-yl)ethyl)amino)hexyl)oxy)benzoic acid (4).**

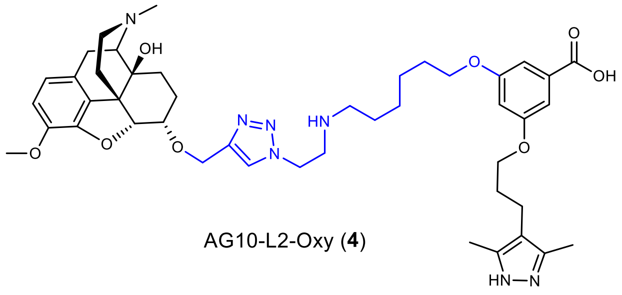

AG10-L2-Oxy (**4**)

The click (CuAAC) coupling was carried out by compound **13** (118 mg, 0.332 mmol, 1 equiv), compound **11** (185.5 mg, 0.332 mmol, 1 equiv), $CuSO_4.5H_2O$ (20.74 mg, 0.083 mmol, 0.25 equiv), and sodium ascorbate (32.9 mg, 0.166 mmol, 0.5 equiv) in a 5 mL mixture of THF/$H_2O$ (4:1). The reaction mixture was stirred at room temperature overnight. The solution was concentrated under reduced pressure. The residue was extracted by hexane:ethyl acetate. The combined organic extracts were concentrated under reduced pressure to afford an intermediate and was used for the next step directly. Then, to a solution of intermediate, was added a mixture containing TFA and

$CH_2Cl_2$, (1:4 ratio) (5 mL) and the reaction mixture was stirred at room temperature for 2 hours. The solution was concentrated under reduced pressure and purified by preparative HPLC to afford compound AG10-L2-Oxy (**4**) (229.8 mg, 0.283 mmol, 85% yield over two steps) (98% purity by HPLC); $t_R$ (C4 column) = 8.2 minutes; $t_R$ (C18 column) = 15.0 minutes; $^1$H NMR (600 MHz, $CD_3OD$) δ 7.81 (s, 1H), 7.14-7.12 (m, 2H), 6.84 (d, 1H, *J* = 8.2 Hz), 6.73 (d, 1H, *J* = 8.2 Hz), 6.68 (t, 1H, *J* = 2.4 Hz), 4.84 (d, 1H, *J* = 5.5 Hz), 4.78-4.74 (m, 3H), 4.64 (d, 1H, *J* = 12.4 Hz), 4.09-4.06 (m, 1H), 4.01-3.96 (m, 4H), 3.81 (s, 3H), 3.61 (t, 2H, *J* = 5.8 Hz), 3.54 (d, 1H, *J* = 5.5 Hz), 3.43-3.35 (m, 1H), 3.16-3.11 (m, 1H), 3.09-3.06 (m, 2H), 2.89 (s, 3H), 2.83-2.78 (m, 1H), 2.70 (t, 2H, *J* = 7.2 Hz), 2.62-2.57 (m, 1H), 2.31 (s, 6H), 2.01-1.97 (m, 2H), 1.93-1.71 (m, 6H), 1.68-1.63 (m, 2H), 1.60-1.52 (m, 3H), 1.50-1.44 (m, 3H); $^{13}$C-NMR (150 MHz, $CD_3OD$) δ 169.62, 148.72, 147.32, 144.89, 144.06, 134.07, 130.95, 125.59, 123.80, 120.31, 117.00, 109.15, 108.91, 107.27, 89.27, 75.44, 71.59, 69.32, 68.85, 67.84, 64.77, 57.53, 47.90, 47.39, 46.09, 41.98, 30.75, 30.12, 30.10, 27.79, 27.35, 27.14, 26.77, 24.91, 22.59, 19.54, 9.89; HRMS (DART) *m/z:* calcd for $C_{44}H_{60}N_7O_8$ $[M+H]^+$ 814.4503; found: 814.4485.

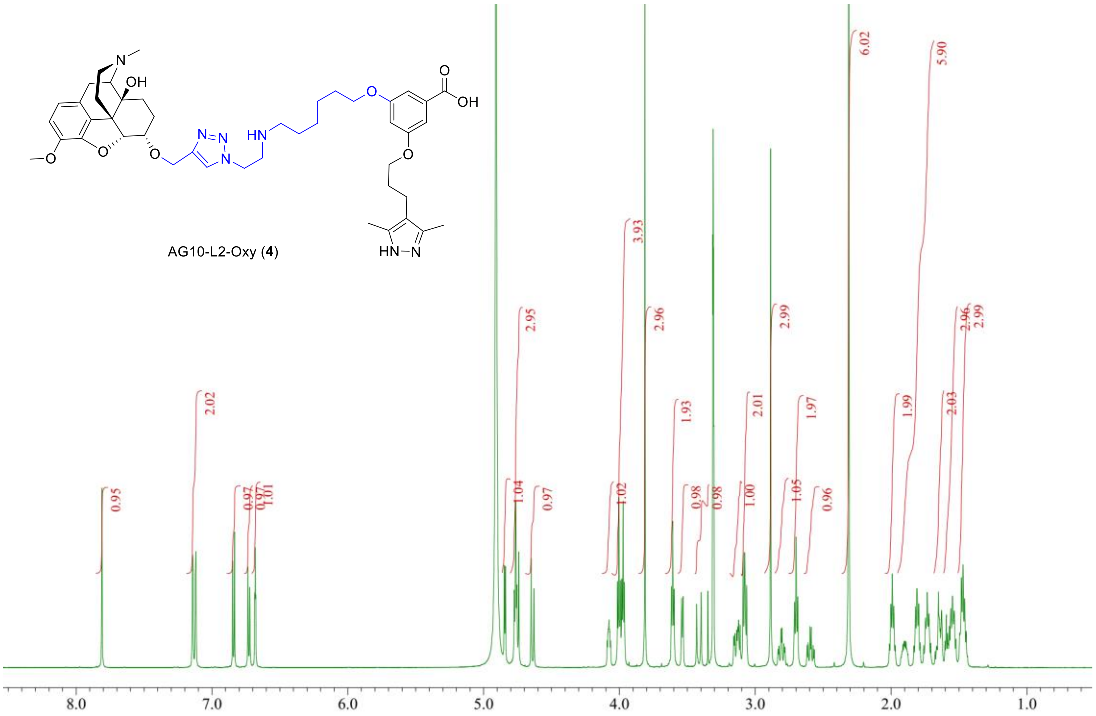


**Supplementary Figure 26.** $^{1}H$ NMR Spectrum for AG10-L2-Oxy (**4**).

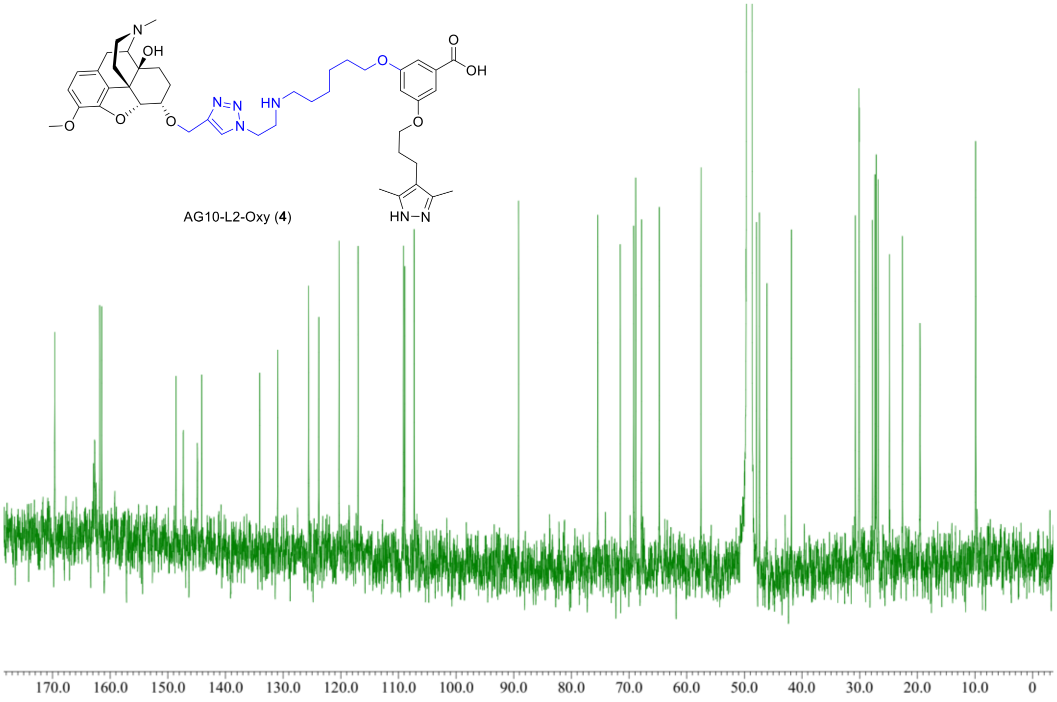


**Supplementary Figure 27.** $^{13}C$ NMR Spectrum for AG10-L2-Oxy (**4**).

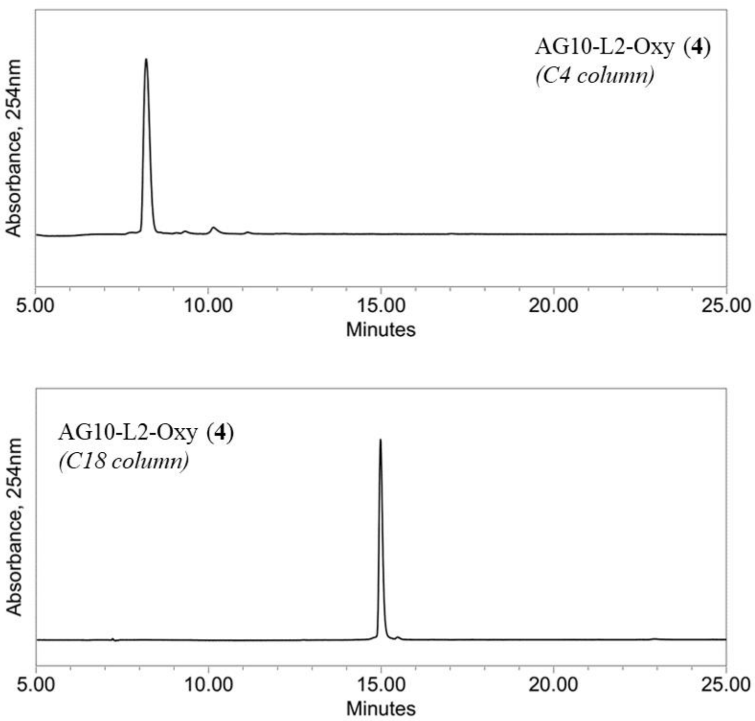


**Supplementary Figure 28.** HPLC traces of AG10-L2-Oxy (**4**).

**Experimental animals.** Approximately 6-8 weeks old normal, jugular vein and intracerebroventricular (ICV) cannulated male Sprague-Dawley rats (226-250 g each) from Charles River Laboratories Inc., Hollister, CA, USA were used for different animal studies. Pre- and post-operative care were performed according to the Charles River Institutional Animal Care and Use Committee (IACUC) policy. Rats were housed individually in standard polycarbonate disposable rat cages with the pre-bedded corn cob and wood shavings. The animal room environment was maintained at 64-79°F, 50-70% relative humidity, and automatic 12-hours light / 12-hours dark cycle (light hours: 7:00 am-7:00 pm). All the animals were acclimated for 5 days upon arrival. They were provided with a standard laboratory diet and normal tap water ad libitum. The animal studies were conducted in accordance with National Institutes of Health (NIH) guidelines for the care and use of live animals and were approved by the Institutional Animal Care and Use Committee (IACUC) at University of the Pacific. The IACUC protocol 19R02 and 21R03 were used in this study.

*Jugular vein cannula implantation.* (Performed by Charles River Laboratories Inc, Hollister, CA). For jugular vein surgery, after performing the proper pre-operative care, a catheter was inserted into the right jugular vein of the rats and was further progressed to the atrium of the heart. Following that, a ligature was placed around the cannulated vascular to secure the catheter in place. Subcutaneously, the catheter was tunneled to the dorsal incision, exteriorized in the scapular region, and secured with a wound clip. The rats were monitored closely, and healthy rats were shipped within 5 days of surgery. Before the day of the experiment, a quick connect™ single luer lock harness from SAI Infusion Technologies was attached to the catheter under isoflurane anesthesia, allowing convenient blood sampling and IV bolus dose administration.

*Intracerebroventricular (ICV) cannula implantation* (Performed by Charles River Laboratories Inc, Hollister, CA). The rats were first placed into stereotaxic apparatus. The bregma and lambda positions of the skull were identified, and four holes for anchoring the screws were made. The guide cannula, made of a threaded cylindrical plastic pedestal molded around a piece of 22G stainless steel, was loaded onto the stereotaxic apparatus holder. The tip of the cannula was directed over the bregma. Subsequently, the zero coordinates were recorded, allowing it to be placed over the specified target as per the predetermined coordinates. After drilling a hole into the skull, the guide cannula was lowered through the hole into the brain. Powdered bone graft material

was applied in a layer to affix the cannula and the exposed portion of the skull was covered. After that, a small amount of bone graft liquid was applied to the powder. Then, the metal wire stylet portion of the dummy cannula was inserted into the guide cannula. The plastic threaded cap of the dummy cannula was used to securely seal it onto guide cannula to prevent any contaminant entry.

**Sample preparation and LC-MS/MS analysis of the experimental compounds.**

*Sample preparation.* The brain was immediately snap frozen in the liquid nitrogen. Plasma samples were prepared by centrifugation of blood at 1500 g for 10 minutes at 4 °C. The CSF, brain and plasma samples were stored in the -80 °C for further analysis. Stock solutions of compound **1** (20 mM), AG10-L2-Nal (**3**) (10 mM), naloxegol (10 mM), naloxone (20 mM), oxycodone (10 mM), and AG10-L2-Oxy (10 mM) were prepared in DMSO solution. The working solutions were prepared by serial dilution of the DMSO solution mentioned above. The calibration curve standards were prepared by spiking aliquots (1 µL) of each working solution to 49 µL blank rat plasma, artificial CSF (125 mM NaCl, 2.5 mM KCl, 1.26 mM $CaCl_2$, and 1.18 mM $MgCl_2$), or blank rat brain homogenate. Quality control (QC) samples prepared at low, medium, and high concentrations were extracted at the same time with the animal samples.

Brain homogenate was prepared by homogenizing the brain tissue with 2 volumes (w:v) of homogenizing solution (PBS buffer). For compound **1**, an aliquot of 50 µL brain homogenate, plasma or CSF sample was precipitated with 4 volumes of extraction buffer (methanol-water (95:5, v/v) containing 0.1% formic acid with 15 ng/mL Chloro-AG10). For naloxone, naloxegol, and AG10-L2-Nal, an aliquot of 50 µL sample was precipitated with 2 volumes of extraction buffer (methanol-water (95:5, v/v) containing 0.1% formic acid with 15 ng/mL naloxone-d5). Oxycodone and AG10-L2-Oxy samples were extracted the same way using oxycodone D6 as the internal standard. The corresponding double blank samples were extracted with the extraction buffer without the internal standard i.e., methanol-water (95:5, v/v) containing 0.1% formic acid. The CSF, brain homogenate and plasma samples were vortexed for 30 seconds, and then kept at -20°C for 5 minutes. Then, the samples were centrifuged at 15000 rpm for 5 minutes; the supernatant was collected and kept in the -20°C freezer for 5 minutes and centrifuged again at 15000 rpm for another 5 minutes. The samples were stored in -80°C for further analysis.

*LC-MS/MS analysis.* 20 μL of samples were injected for LC-MS/MS analysis. Agilent 1200 HPLC coupled with a triple quadrupole mass spectrometer (AB SCIEX API 3000™) was used to quantitate the analytes in the plasma, brain, and CSF samples. The mobile phase was composed of solvent A consisting of methanol-water (5:95, v/v) containing 0.1% formic acid and solvent B consisting of methanol-water (95:5, v/v) containing 0.1% formic acid, at a flow rate of 0.5 mL/minute, and the injection volume was 20 μL. LC-MS/MS analysis was performed on a Waters™ XBridge C18 (4.6x150 mm, 5 μm) using gradient methods for all the compounds. The turbo spray ion source was set in the positive ionization mode. Fragmentation pattern and peak areas were used to identify and quantitate the test compounds, respectively. LC-MS/MS data were collected from Analyst 1.5 software (Applied Biosystems/MDS Analytical Technologies).

For the quantitation of compound **1**, Chloro-AG10 was used as the internal standard. The nebulizer gas (NEB), curtain gas (CUR), collision gas (CAD), ion spray voltage (IS), and temperature (TEM) were set as 10, 10, 10, 5000 and 425, respectively. LC-MS/MS analysis was performed using a gradient method increasing linearly from 0-80% solvent B in 0-5 minutes, stayed at 80% solvent B for 7 minutes, then went down from 80-0% solvent B in 1 minute and finally stayed at 0% solvent B for 1 minute. The retention time for compound **1** and Chloro-AG10 was 9.2 minutes and 11.1 minutes, respectively.

For quantitation of naloxone, naloxegol, and AG10-L2-Nal, naloxone-d5 was used as the internal standard. The source parameters of nebulizer gas (NEB), curtain gas (CUR), collision gas (CAD), ion spray voltage (IS) and temperature (TEM) were 14, 10, 10, 2000, and 425, respectively. The total run was 10.5 minutes. The HPLC chromatography was programmed to start with 0-100% solvent B for 0-5 minutes, then stays at 100% solvent B at 5-8.5 minutes, followed by 100-0% solvent B at 8.5-9.5 minutes, and finally at 0% solvent B at 9.5-10.5 minutes. The retention times for naloxone, naloxone-d5, naloxegol, and AG10-L2-Nal were 6.45 minutes, 6.45 minutes, 7.45 minutes, and 7.66 minutes, respectively.

Oxycodone and AG10-L2-Oxy were quantitated using oxycodone-D6 as the internal standard. The source parameters of nebulizer gas (NEB), curtain gas (CUR), collision gas (CAD), ion spray voltage (IS) and temperature (TEM) were 14, 10, 8, 4000 and 400 respectively. The total run was

11 minutes. The HPLC chromatography was programmed to start with 0-100% solvent B for 0-5 minutes, then stays at 100% solvent B at 5-8 minutes, followed by 100-0% solvent B at 8-9 minutes, and finally at 0% solvent B at 9-11 minutes. The retention times for oxycodone, oxycodone-D6, and, AG10-L2-Oxy were 7.01, 7.01, and 7.64 minutes, respectively.

Based on the calibration curves for these compounds in plasma, brain, and CSF and the internal standard generated by the LC−MS/MS analyst, mean (±s.d.) concentrations of test compounds were determined. Samples were diluted when the concentration of the samples was outside the calibration curve range.

The detailed mass spectrometer conditions for multiple reaction monitoring (MRM) of each compound are listed in the Supplementary Table 1. The lower limit of quantitation (LLOQ) of compound **1** was 6.4 nM in all the matrices. The lower limit of quantitation (LLOQ) of AG10-L2-Nal and naloxone was 3 and 7.6 nM, respectively in all matrices. For naloxegol, the LLOQ was 2 nM in plasma and 3.8 nM in the CSF and the brain tissue. The LLOQ for oxycodone was 5 nM for CSF and 25 nM for both plasma and brain tissue. For AG10-L2-Oxy, the LLOQ was 10 nM for plasma and brain tissue and 25 nM for the CSF. The linear ranges were calculated based on the regression analysis between the analyte and internal standard peak area ratio and the analyte concentration (fig. S2, S3, and S7). The accuracy was between 80% and 120% in every case.

**In silico modeling studies.** The in-silico modeling studies were carried out using TTR-AG10 crystal structure and mu-opioid receptor bound to a morphinan antagonist[2, 3]. The geometry optimization of the AG10-naloxone conjugates was carried out at the hybrid density functional B3LYP level[4] with 6-31G(d)[5, 6] basis set using the Gaussian 09[7] program package. To confirm the optimized geometry is at a minimum, frequency calculations were carried out on the optimized geometries. The docking experiments were carried out using Dock6[8]. The crystal structure of TTR (pdb id: 4HIQ)[9] and the crystal structure of the mu-opioid receptor bound to a morphinan antagonist (pdb id: 4DKL)[10] were obtained from RCSB.org. The UCSF Chimera (Version 1.3) program[11] was used to analyze and visualize the proteins and docking complex structures.

***In vitro* pharmacology: opioid receptor binding assays.** *In vitro* mu-, kappa-, and delta-opioid receptor binding assays were performed by Eurofins Cerep in their France site. Naloxone, naloxegol, AG10-L1-Nal, AG10-L2-Nal, oxycodone, and AG10-L2-Oxy were tested for mu-opioid receptor binding. AG10-L2-Nal and AG10-L2-Oxy were also tested for kappa- and delta-opioid receptor binding.

*Human mu-opioid receptor binding (agonist radioligand) assay*. Expression of human mu-opioid receptor (h-MOR) revealed high affinity recognition of the mu-opiate specific ligand [$^{3}$H]-DAMGO (D-ala2, N-methyl-phe4, glyo15) enkephalin. This binding can be displaced by compounds recognizing mu-opioid receptors with high affinity. In this assay [$^{3}$H]-DAMGO was used as a ligand at 0.5 nM concentration with a $K_d$ value of 0.35 nM. The control inhibitor was DAMGO. The testing concentration of naloxone, AG10-L1-Nal and AG10-L2-Nal were 0.01, 0.1, 1, 10, and 100 nM. For naloxegol, the testing concentrations were 0.1, 1, 10, 100 and 1000 nM. For oxycodone the testing concentrations were 0.01, 0.1, 1, 10, 100 and 500 nM. And for AG10-L2-Oxy, the testing concentrations were 0.1, 1, 10, 100, 500 and 1000 nM. The incubation time for the assay was 120 minutes at room temperature. AG10-L1-Nal and AG10-L2-Nal binding to the mu-opioid receptor was also tested in the presence of TTR (1 µM).

*Analysis and expression of results.* The results are expressed as a percent inhibition of control specific binding obtained in the presence of the test compounds.

$$100 - (\frac{measured\ specific\ binding}{control\ specific\ binding} \times 100)$$

The $IC_{50}$ values and Hill coefficients (nH) were determined by non-linear regression analysis of the competition curves generated with mean replicate values using Hill equation curve fitting (Supplementary equation 1).

**Supplementary Equation 1.** Modified Hill equation for four-parameter logistic nonlinear regression model.

$$Y = D + [\frac{A - D}{1 + \left(\frac{C}{C_{50}}\right)^{nH}}]$$

where Y = specific binding, A = left asymptote of the curve, D = right asymptote of the curve, C = compound concentration, $C_{50}$ = $IC_{50}$, and nH = slope factor. This analysis was performed using software developed at Cerep (Hill software) and validated by comparison with data generated by the commercial software SigmaPlot® 4.0 for Windows® (© 1997 by SPSS Inc.). The inhibition constants ($K_i$) were calculated using the Cheng-Prusoff equation (Supplementary equation 2).

**Supplementary Equation 2.** Cheng-Prusoff equation.

$$K_i = \frac{IC_{50}}{(1 + \frac{L}{K_d})}$$

where L = concentration of radioligand in the assay and $K_d$ = affinity of the radioligand for the receptor. A scatchard plot is used to determine the $K_d$.

Results showing an inhibition higher than 50% are considered to represent significant effects of the test compounds. 50% is the most common cut-off value for further investigation (determination of $IC_{50}$ values from concentration-response curves).

*Human delta and rat kappa-opioid receptor (agonist radioligand) binding assays.* For the delta-opioid receptor (human) binding assay, [$^3$H]-DADLE was used as a ligand at 0.5 nM concentration with a $K_d$ value of 0.6 nM. The control inhibitor was DPDPE. The testing concentrations of AG10-L2-Nal were 0.1, 1, 10, 50, and 100 nM in duplicate. For AG10-L2-Oxy, the testing concentrations were 1, 10, 100, 1000, and 2000 nM. The incubation time for the assay was 60 minutes at room temperature. In the case of the kappa opioid receptor (rat) binding assay, [$^3$H] U 69593 was used as a ligand at 1 nM concentration with a $K_d$ value of 2 nM. U 50488 hydrochloride was the control inhibitor in this case. The testing concentrations of AG10-L2-Nal were 0.1, 1, 10, 50, and 100 nM in duplicate. The incubation time for the assay was 60 minutes at room temperature. $IC_{50}$ and $K_d$ values were calculated the same way as the µ-opioid receptor binding assay.

**Functional mu-opioid receptor activity assays.** Human recombinant mu-opiate receptor stably expressed in CHO-K1 cells were used. The experiments were performed at Eurofins, Panlabs (Taiwan). The experimental procedure was performed as reported[12]. Test compound or vehicle was pre-incubated with the membrane preparation at 0.041 mg/ml and 3 μM GDP in 20 mM HEPES, pH 7.4, 100 mM NaCl, 10 mM MgCl2, 1 mM DTT, 1 mM EDTA for 20 minutes at 25°C followed by SPA beads for another 60 minutes at 30°C. The reaction was initiated by adding 0.3 nM [35S]GTPγS for an additional 30 minutes incubation period. CPM was quantitated by scintillation counter and the % response/inhibition was calculated relative to control response, 10 μM and 0.1 μM DAMGO, in agonist and antagonist mode, respectively. Naltrexone was used as an antagonist reference. For the [$^{35}$S]GTPγS competitive functional assay, morphine was tested (in the absence and presence of 0.4 μM AG10-L2-Nal) at concentrations ranging from 200 μM to 0.01μM. The experiments were conducted at 10 concentrations (each concentration was tested in duplicate experiments).

**Serum stability assay of AG10-L2-Nal.** AG10-L2-Nal 100 μM samples were incubated in human, mouse, or rat sera at 37°C. 100 μL samples were analyzed at 0 h, 24 h, and 48 h time intervals. All the samples were processed by adding 200 μL of solvent B (95% methanol and 0.1% trifluoroacetic acid in water) followed by centrifuging at 15,000 rpm for 5 minutes. The supernatant was kept in the -20°C freezer for 5 minutes and centrifuged again at 15000 rpm for another 5 minutes. The supernatant was analyzed using Waters™ XBridge C18 column (4.6x250 mm, 5μm) on Waters e2695 separations module HPLC system connected to a Waters 2998 photodiode array detector operating between the UV ranges of 200-400 nm. HPLC analysis was performed using a gradient method increasing linearly from 0-100% solvent B in 20 minutes. The mobile phase was composed of solvent A consisting of acetonitrile-water (5:95, v/v) containing 0.1% trifluoroacetic acid and solvent B, at a flow rate of 0.5 mL/minute.

**Long-term stability assay of AG10-L2-Nal in PBS buffer**. We also tested the long-term chemical stability, which demonstrated that AG10-L2-Nal is stable in buffer for a least one month at 37 °C. AG10-L2-Nal (100 μM) was incubated in PBS buffer at 37 °C, and samples (100 μL) were analyzed weekly for four weeks. The samples were processed the same way as the AG10-L2-Nal serum stability assay.

**Hot plate analgesia assays**

*Hot plate analgesia test after IV administration of drugs.* Rats were arbitrarily assigned to treatment groups (n = 6 rats per group) as follows: saline group (received 0.9% sterile saline followed by vehicle), control group (received morphine 10 mg/kg which is equivalent to 35 µmol/kg, followed by vehicle), naloxone 35, 3.5, and 0.7 µmol/kg groups, naloxegol 35, 3.5, and 0.7 µmol/kg groups, methylnaltrexone 35, 3.5, and 0.7 µmol/kg groups, or AG10-L2-Nal 35 µmol/kg group. At time = 0 minute, each rat received one 200 µL intravenous dose of 0.9 % sterile saline (only the saline group) or morphine 35 µmol/kg dissolved in 0.9% sterile saline followed by an injection of 200 µL sterile saline to flush the jugular vein cannula. Except the saline group, every other group received the morphine dose (35 µmol/kg). 5 minutes after the saline or morphine dose, all the rats received another 200 µL intravenous injection of either vehicle (in saline and control groups) or the different opioid antagonist (naloxone, naloxegol, methylnaltrexone, and AG10-L2-Nal) doses containing 10% DMSO, 20% PEG400, and 70% sterile deionized water. Afterwards, an injection of 200 µL sterile saline was given to each rat to flush the jugular vein cannula. To assess the dose in which morphine showed potent analgesia in a consistent manner and naloxone affected the antinociceptive properties of morphine, a dose range-finding test was conducted before the start of the main study (data not shown). All the doses were formulated fresh on the day of the experiment and were kept at room temperature protected from light until use. Each animal was submitted to the analgesia meter 60 minutes after 0.9% saline or morphine 35 µmol/kg doses.

*Hot plate latency test after subcutaneous administration to check the partial agonistic behavior of the molecules.* Rats were arbitrarily assigned to treatment groups (n = 5 rats per group) as follows: vehicle, morphine 35 µmol/kg, and AG10-L2-Nal 35 µmol/kg groups. At time = 0 minute, each rat received one 500 µL subcutaneous dose (per 250 g body weight) of vehicle (composed of 10% DMSO, 20% PEG400, and 70% sterile deionized water), morphine 35 µmol/kg, or AG10-L2-Nal 35 µmol/kg dissolved in vehicle. Hot plate latency was measured 1 hour after the dose.

*Hot plate latency test after intracerebroventricular and/or subcutaneous AG10-L2-Nal administration.* ICV cannulated male Sprague Dawley rats (201–225 g; 6-7 weeks old) from Charles River were used in this experiment. The experimental conditions of hot plate analgesia

test were the same as the intravenous one except the dosing schedule and experimental groups. Rats were arbitrarily assigned to the following treatment groups (n = 5 rats per group): saline group (received ICV vehicle dose followed by a SC vehicle dose and a SC saline dose), control group (received ICV vehicle dose followed by a SC vehicle dose and a SC morphine dose), AG10-L2-Nal SC group (received ICV vehicle dose followed by a SC AG10-L2-Nal dose and a SC morphine dose), AG10-L2-Nal ICV group (received ICV AG10-L2-Nal dose followed by a SC vehicle dose and a SC morphine dose), and AG10-L2-Nal ICV+SC group (received ICV AG10-L2-Nal dose followed by a SC AG10-L2-Nal dose and a SC morphine dose). The ICV AG10-L2-Nal dose was 0.35 μmol/kg which is equivalent to 88 nmol per rat. The subcutaneous morphine and AG10-L2-Nal doses were 35 μmol/kg. At time = 0 minute, each rat received 10 μL ICV dose of vehicle or AG10-L2-Nal followed by a SC dose of vehicle or AG10-L2-Nal. Then, at 10 minutes, 0.9% sterile saline (only for the saline group) or morphine 35 μmol/kg dissolved in 0.9% sterile saline dose was administered subcutaneously. Hot plate latency was measured 1 hour after the saline or morphine doses. The dosing volume for the SC vehicle/AG10-L2-Nal and SC saline/morphine were 500 μL and 200 μL per 250 g rats, respectively. While manipulating cannula, all the procedures were performed using aseptic techniques. For the ICV dosing, the injector cannula was attached to a PE50 tubing. The other end of the tubing was then attached to a 23-gauge needle. The ICV injections were performed using Hamilton micro-syringes. All the materials and parts were sterile while performing injection.

*Hot plate latency test after intracerebroventricular and/or subcutaneous AG10-L2-Oxy administration.* ICV cannulated male Sprague Dawley rats (201–225 g; 6-7 weeks old) from Charles River were used in this experiment. The experimental conditions of this hot plate analgesia test were same as the ICV and/or SC AG10-L2-Nal experiment in the previous section except the dosing schedule and experimental groups. The reported time to run the GI transit assay is 30 minutes (time for charcoal to have significant passage across the small intestine of rats). From our dose-response finding pilot study, no analgesic effect was found for oxycodone 1 hours after ICV dosing (even at a high ICV dose of 1280 nmol). Similar data have been reported in the literature where the analgesic effect of oxycodone in rats diminished rapidly 30 minutes after ICV dosing (The analgesic effect after 1 hour was less than 10% of the peak analgesics effect at 15 minutes)[13]. When we reduced the total study time from 1 hour to 40 minutes in the hot plate study, we observed

significant analgesia only at the higher dose of oxycodone (1280 nmol per rat) and also at 160 nmol of AG10-L2-Nal. That's why the hot plate and GI transit assays shown in Fig. 8d and 8e were conducted 40 minutes post-drug administration (i.e., to allow enough time to observe analgesia and the minimum requirement of 30 minutes for the GI transit assay). Rats were arbitrarily assigned to the following treatment groups (n = 5 rats per group): vehicle (intracerebroventricular route, ICV), oxycodone (16 µmol/kg; subcutaneous route, SC), oxycodone (640 and 1280 nmol per rat; intracerebroventricular route, ICV), AG10-L2-Oxy (64 µmol/kg; subcutaneous route, SC), or AG10-L2-Oxy (160 nmol; intracerebroventricular route, ICV). At time = 0 minute, each rat received ICV (10 µL) or SC (500 µL) doses of vehicle or opioid agonists per 250 g rats. Hot plate latency was measured at 40 minutes after the vehicle or agonist doses.

*Hot plate assay for evaluating the effect of multiple morphine doses in the presence of antagonists after subcutaneous administration.* Normal male Sprague Dawley rats were used (n = 6 rats per group). We administered saline or two doses of 35 µmol/kg morphine, the first dose was at 0 hour and the second dose was administered 1.5 hours after the first dose. The vehicle or opioid antagonists were dosed one time only at t = 5 minutes. All the doses were administered subcutaneously. The saline group received 0.9% sterile saline at 0-minute, vehicle (composed of 10% DMSO, 20% PEG400, and 70% sterile deionized water) at 5 minutes, and another 0.9% sterile saline dose at 1.5 hours. Control, naloxegol, and AG10-L2-Nal groups received 35 µmol/kg morphine at 0 minute, followed by vehicle or naloxegol 3.5 µmol/kg or AG10-L2-Nal 35 µmol/kg dose at 5 minutes, respectively, and the second morphine dose (35 µmol/kg) at 1.5 hours. The hot plate latency was measured 2.5 hours after the first saline or morphine dose. 3.5 µmol/kg dose was chosen for naloxegol in this study since in the IV GI transit assay, there was no statistically significant difference between the morphine 35 µmol/kg and naloxegol 0.7 µmol/kg doses.

*Hot plate study of AG10-L2-Nal and naloxegol against fentanyl in SC route of administration.* Male Sprague Dawley rats were used for this study. Rats were first administered with a single SC dose of vehicle or the opioid antagonists. After 30 minutes, animals were administered with a single SC dose of saline or fentanyl (1 mg/kg). Rats were arbitrarily assigned into following treatment groups (n = 5 rats per group)- saline group: vehicle + saline; control group:  vehicle + fentanyl (1 mg/kg); all other groups: specified dose of antagonists + fentanyl (1 mg/kg). The dosing

volume of vehicle/antagonists were 500 µL, and the dosing volume of saline/fentanyl was 200 µL. The hot plate withdrawal latency to heat exposure (withdrawal or shaking of the hind paw, sharp withdrawal, licking of fore or hind paw, or attempting to escape by jumping) was recorded 1 hour after the fentanyl dose.

*Hot plate assay of AG10-L2-Nal with AG10 against morphine in SC route of administration.* Male Sprague-Dawley rats (n = 5 rats per group) were first administered with a single SC dose of vehicle or the 50 mg/kg AG10. After 10 minutes, animals were administered with a single subcutaneous dose of saline or 10 mg/kg morphine (35 µmol/kg). After another 5 minutes, animals were administered with AG10-L2-Nal or vehicle. Saline group: vehicle + saline + vehicle; control group: vehicle + 10 mg/kg morphine + vehicle; AG10-L2-Nal group: vehicle + 10 mg/kg morphine (35 µmol/kg) + 30 mg/kg AG10-L2-Nal (35 µmol/kg); AG10-L2-Nal +AG10 group: AG10 50 mg/kg + 10 mg/kg morphine (35 µmol/kg) + 30 mg/kg AG10-L2-Nal (35 µmol/kg). The dosing volume of vehicle/antagonists were 500 µL, and the dosing volume of saline/fentanyl was 200 µL. The hot plate withdrawal latency to heat exposure (withdrawal or shaking of the hind paw, sharp withdrawal, licking of fore or hind paw, or attempting to escape by jumping) was recorded 1 hour after the morphine dose before the rats were removed from the hot plate.

**Gastrointestinal (GI) transit assays**

*GI transit study after intravenous administration of the molecules.* Jugular vein cannulated rats were arbitrarily assigned to treatment groups (n = 6 rats per group) as follows: saline group: saline + vehicle; control group: 35 µmol/kg morphine + vehicle; all other groups: 35 µmol/kg morphine + specified dose of antagonists. Rats were first administered with saline or a single intravenous (IV) dose of morphine (35 µmol/kg). After 5 minutes, the morphine treated animals were administered with a single intravenous dose of vehicle or the opioid antagonists. After each dose, an injection of 200 µL sterile saline was administered to flush the jugular vein cannula. 30 minutes after the saline or morphine administration, 1 mL of a charcoal suspension was administered to each animal by oral gavage. 30 minutes after the charcoal ingestion, each rat was humanely euthanized by decapitation, and the intestine was exposed. The distance the charcoal had traveled along the intestine from the pyloric sphincter and the total intestinal length were measured. The distance travelled by the charcoal meal in millimeters was calculated as a percentage of the total

length of the intestine for each rat. Opioid antagonist (naloxone, naloxegol, methylnaltrexone, and AG10-L2-Nal) doses were formulated in 10% DMSO, 20% PEG400, and 70% sterile deionized water. All the doses were formulated fresh on the day of the experiment and were kept at room temperature protected from light until use.

*GI transit assay to check the partial agonistic behavior of AG10-L2-Nal.* This assay was performed the similar way as the intravenous GI transit assay with exception to the dosing schedule (n = 5 rats per group). The treatment groups were the same as the subcutaneous hot plate analgesia test above to check the agonistic characteristic of the molecules. 30 minutes after the treatment administration, 1 mL of charcoal suspension was administered to each animal by oral gavage. Each rat was humanely euthanized 30 minutes after the charcoal ingestion, the intestine was exposed, and the GI transit was measured.

*GI transit assay after intracerebroventricular (ICV) and/or subcutaneous AG10-L2-Nal administration.* The experimental groups were the same as the hot plate latency test stated above (n = 5 rats per group). The distance traveled by a charcoal within the small intestine was measured at 1 hour after the saline or morphine dose. The dosing schedule is the same as the above hot plate study, except in this experiment each rat received a 1 mL charcoal suspension by oral gavage 30 minutes after the saline or morphine dose.

*GI transit assay after intracerebroventricular (ICV) and/or subcutaneous AG10-L2-Oxy administration*. The dosing groups for this study were the same as the hot plate ICV and/or SC AG10-L2-Oxy assay (n = 5 rats per group). At time = 0 minutes, each rat received 10 µL ICV or 500 µL doses of vehicle or opioid agonist per 250 g rats. Charcoal meal was given at 10 minutes. GI transit was measured at 40 minutes after the treatment doses.

*Gastrointestinal (GI) transit assay for evaluating the OIC effect of multiple morphine doses in the presence of naloxegol and AG10-L2-Nal after subcutaneous administration.* The dosing groups for this study were the same as the subcutaneous hot plate nociception model with multiple morphine and single vehicle/opioid antagonist doses (n = 6 rats per group). The charcoal meal was

given 30 minutes after the first saline or morphine dose. The distance the charcoal had traveled was measured 2.5 hours after the saline or morphine dose.

*Gastrointestinal (GI) transit assay of AG10-L2-Nal and naloxegol against fentanyl in subcutaneous route of administration.* Male Sprague Dawley rats were used for this study (n = 5 rats per group). The dosing groups for this study were same as the hot plate study of AG10-L2-Nal and naloxegol against fentanyl in subcutaneous route of administration. Rats were first administered with a single SC dose of vehicle or the opioid antagonists. After 30 minutes, animals were administered with a single SC dose of saline or fentanyl (1 mg/kg). 30 minutes after saline/fentanyl dose, 1 mL of charcoal suspension was administered to each rat by oral gavage. Each rat was humanely euthanized 30 minutes after the charcoal ingestion, and the GI transit was measured.

*Gastrointestinal (GI) transit assay of AG10-L2-Nal with AG10 against morphine in SC route of administration.* Male Sprague Dawley rats were used for this study (n = 5 rats per group). The dosing schedule and groups for this study were same as the hot plate assay of AG10-L2-Nal with AG10 against morphine in SC route of administration except for the fact that 1 mL charcoal meal was administered 30 minutes after the saline or morphine dose. Each rat was euthanized 30 minutes after the charcoal ingestion, and the GI transit was measured.

*Gastrointestinal (GI) transit study of loperamide.* Gastrointestinal (GI) transit assay at 1 hour after SC doses of the test compounds. Male Sprague-Dawley rats (n = 5 rats per group) were first administered with a single subcutaneous (SC) dose of saline or loperamide 0.5 mg/kg or loperamide 1 mg/kg or 1 mg/kg fentanyl doses. The animals received an oral gavage of charcoal meal 30 minutes after the saline or loperamide doses. At 1 hour, the rats were euthanized, the intestine was exposed, and the GI transit was measured. The dosing volume of saline were 500 μL, and the dosing volume of saline/fentanyl was 200 μL.

**Evaluating the effect of AG10-L2-Nal on Holo-RBP-TTR interaction in human serum.** A solution of thyroxine (T4), compound **1**, AG10-L2-Nal (1 μL of 2 mM stock solution in DMSO) or control (1 μL DMSO) was added (final compound concentration 20 μM) to 99 μL of human

serum (from human male AB plasma, Sigma; TTR concentration ~5 μM). The treated serum was incubated at 37 °C for 2 hours. After the incubation, all samples were analyzed using Western blot using a procedure reported earlier[3]. In this assay, 10 μL of the serum incubated with test compounds was added to 90 μL of buffer A (pH 7.0 PBS, 100 mM KCl, 1 mM EDTA, 1 mM DTT). For the urea sample, 10 μL of the control serum (incubated with DMSO) was added to 90 μL of urea buffer (buffer A containing 8 M urea). All serum samples were then cross-linked with glutaraldehyde (final concentration of 2.5%) for 5 minutes, and then quenched with 10 μL of 7% sodium cyanoborohydride solution in 0.1 M NaOH. The samples were denatured by adding 100 μL of SDS gel loading buffer and boiled for 5 minutes. 10 μL of each sample was separated in 16% SDS-PAGE gels. The gel was transferred using wet transfer (Bio-Rad; buffer: 3.03 g of Tris, 14.4 g of glycine, 200 mL methanol, 800 mL water). Membrane was blocked in blocking buffer (Sea-block blocking buffer, Fisher) for 30 minutes at room temperature. The membrane was then incubated in anti-RBP antiserum at 1:500 dilution overnight at 4°C. After incubation, the membrane was washed four times for 5 minutes each in 0.1% Tween-20 PBS at room temperature. Then the membrane was incubated in IRdye800 donkey antirabbit secondary antibody at 1:15000 dilution in blocking buffer for 2 hours at room temperature. After incubation, the membrane was washed in a similar manner as above and scanned using a LI-COR Odyssey CLx Imaging System for quantification. The Western blot membrane image was analyzed by Image Studio Ver 5.2.5 software for quantification. The free RBP band (at ~21 kDa) was quantified easily since it was well separated from the RBP-TTR complex (at ~77 kDa), which is also detected by the antiRBP antiserum.

**Evaluation of the *in vitro* cytotoxicity of naloxone, naloxegol and AG10-L2-Nal.** 3-(4,5-Dimethylthiazol-2-yl)-2,5 diphenyltetrazolium bromide (MTT) assay was performed using CellTiter 96 Non-Radioactive Cell Proliferation Assay (Promega) to determine cell viability. Jurkat and Hep3B cells were cultured in RPMI-1640 and advanced MEM medium respectively supplemented with 10% fetal bovine serum, penicillin/streptomycin (100 unit/mL and 100 μg/mL, respectively), and 1% L-glutamine under the humidified atmosphere of 5% $CO_2$ and 95% air at 37°C. The cells were grown to confluence, trypsinized, and seeded into 96-well plates at a density of ~5000 cells/well. The cells were then treated with naloxone, naloxegol, AG10-L2-Nal (each at 10 and 100 μM), or 1 μM doxorubicin as a positive control. Control cells were also treated with

the appropriate concentration of vehicle (DMSO). After 72-hour incubation at 37°C, cell viability was determined following the standard CellTiter 96 NonRadioactive Cell Proliferation Assay protocol. In short, MTT dye solution was added and incubated for an additional 4 hours at the same temperature. Stop solution was added equally to all wells to dissolve any crystals formed, and absorbance was measured at 570 nm using the SpectraMax M5 (Molecular Devices).

**Supplementary References**


1. Müller C, Farkas R, Borgna F, Schmid RM, Benešová M, Schibli R. Synthesis, Radiolabeling, and Characterization of Plasma Protein-Binding Ligands: Potential Tools for Modulation of the Pharmacokinetic Properties of (Radio)Pharmaceuticals. *Bioconjug. Chem.* **28**, 2372-2383 (2017).
2. Pal A, *et al.* Hydrophilic Small Molecules That Harness Transthyretin To Enhance the Safety and Efficacy of Targeted Chemotherapeutic Agents. *Mol. Pharm.* **16**, 3237-3252 (2019).
3. Penchala SC, *et al.* A biomimetic approach for enhancing the in vivo half-life of peptides. *Nat. Chem. Biol.* **11**, 793-798 (2015).
4. Bencharit S, *et al.* Crystal structure of human carboxylesterase 1 complexed with the Alzheimer's drug tacrine: from binding promiscuity to selective inhibition. *Chem. Biol.* **10**, 341-349 (2003).
5. McLean A, Chandler G. Contracted Gaussian basis sets for molecular calculations. I. Second row atoms, Z= 11–18. *J. Chem. Phys.* **72**, 5639-5648 (1980).
6. Krishnan R, Binkley JS, Seeger R, Pople JA. Self‐consistent molecular orbital methods. XX. A basis set for correlated wave functions. *J. Chem. Phys.* **72**, 650-654 (1980).
7. Frisch M, *et al.* Gaussian 09 Citation. Gaussian. *Inc., Wallingford CT*, (2009).
8. Lang PT, *et al.* DOCK 6: Combining techniques to model RNA–small molecule complexes. *RNA* **15**, 1219-1230 (2009).
9. Penchala SC, *et al.* AG10 inhibits amyloidogenesis and cellular toxicity of the familial amyloid cardiomyopathy-associated V122I transthyretin. *Proc. Natl. Acad. Sci. U. S. A.* **110**, 9992-9997 (2013).
10. Manglik A, *et al.* Crystal structure of the µ-opioid receptor bound to a morphinan antagonist. *Nature* **485**, 321-326 (2012).
11. Pettersen EF, *et al.* UCSF Chimera—a visualization system for exploratory research and analysis. *J. Comput. Chem.* **25**, 1605-1612 (2004).
12. Alt A, Mansour A, Akil H, Medzihradsky F, Traynor J, Woods J. Stimulation of Guanosine-5′-O-(3-[35S] Thio) Triphosphate Binding by Endogenous Opioids Acting at a Cloned MuReceptor. *J. Pharmacol. Exp. Ther.* **286**, 282-288 (1998).
13. Kuo A, Wyse B, Meutermans W, Smith M. In vivo profiling of seven common opioids for antinociception, constipation and respiratory depression: no two opioids have the same profile. *Br. J. Pharmacol.* **172**, 532-548 (2015).
