# Supplementary material for: Peripherally restricted transthyretin-based delivery system for probes and therapeutics avoiding opioid-related side effects
Source: Nat Commun. 2022 Jun 23;13:3590. doi: 10.1038/s41467-022-31342-z (PMC9226319; doi:10.1038/s41467-022-31342-z)
Supplement: Supplementary file 2 — Reporting Summary [file 41467_2022_31342_MOESM2_ESM.pdf]

nature portfolio

Corresponding author(s): Mamoun M. Alhamadsheh
Last updated by author(s): May 11, 2022

Reporting Summary

Nature Portfolio wishes to improve the reproducibility of the work that we publish. This form provides structure for consistency and transparency in reporting. For further information on Nature Portfolio policies, see our Editorial Policies and the Editorial Policy Checklist.

Statistics

For all statistical analyses, confirm that the following items are present in the figure legend, table legend, main text, or Methods section.

n/a	Confirmed	
☐	☒	The exact sample size (*n*) for each experimental group/condition, given as a discrete number and unit of measurement
☐	☒	A statement on whether measurements were taken from distinct samples or whether the same sample was measured repeatedly
☐	☒	The statistical test(s) used AND whether they are one- or two-sided<br>*Only common tests should be described solely by name; describe more complex techniques in the Methods section.*
☒	☐	A description of all covariates tested
☐	☒	A description of any assumptions or corrections, such as tests of normality and adjustment for multiple comparisons
☐	☒	A full description of the statistical parameters including central tendency (e.g. means) or other basic estimates (e.g. regression coefficient) AND variation (e.g. standard deviation) or associated estimates of uncertainty (e.g. confidence intervals)
☐	☒	For null hypothesis testing, the test statistic (e.g. *F*, *t*, *r*) with confidence intervals, effect sizes, degrees of freedom and *P* value noted<br>*Give P values as exact values whenever suitable.*
☒	☐	For Bayesian analysis, information on the choice of priors and Markov chain Monte Carlo settings
☒	☐	For hierarchical and complex designs, identification of the appropriate level for tests and full reporting of outcomes
☒	☐	Estimates of effect sizes (e.g. Cohen's *d*, Pearson's *r*), indicating how they were calculated

Our web collection on statistics for biologists contains articles on many of the points above.

Software and code

Policy information about availability of computer code

Data collection	LC-MS/MS data were collected from Analyst 1.5 software (Applied Biosystems/MDS Analytical Technologies). We used SoftMax® Pro software v5.4.1 (Molecular Devices, Inc.) to collect the fluorescence data. The prep-HPLC purification was performed on a Waters Delta 600 HPLC system connected to a photodiode array detector, using Waters Masslynx V4.1 software. Hot plate latency data were measured using the hot plate analgesia meter of Columbus Instruments, Ohio, USA. 1H NMR and 13C NMR spectra were recorded on a JEOL JNM–ECA 600 spectrometer. In the Western blot procedure, the membranes were scanned by LI-COR Odyssey® CLx Imaging System.
Data analysis	Data were analyzed and plotted using the GraphPad Prism V9.3.1.471 software (La Jolla, CA, USA). The power analysis was performed by G*Power software (Version 3.0.10). The UCSF Chimera (Version 1.3) program was used to analyze and visualize the proteins and docking complex structures. 1H NMR and 13C NMR spectra were analyzed using Delta v5.3 NMR Processing and Control software. CombiFlash® Rf+ Lumen version 2.1.33 was used to analyze the purification process of flash column chromatography of the synthesized molecules. Purity spectra of the analytical-HPLC were obtained from the Waters, Inc. Empower 3 software. The Western blot membrane image was analyzed by Image Studio Ver 5.2.5 software for quantification. A non-compartmental analysis model using Phoenix WinNonlin (version 8) was used to obtain all the intravenous and subcutaneous pharmacokinetic parameters from their plasma concentration-time data.

For manuscripts utilizing custom algorithms or software that are central to the research but not yet described in published literature, software must be made available to editors and reviewers. We strongly encourage code deposition in a community repository (e.g. GitHub). See the Nature Portfolio guidelines for submitting code & software for further information.

Data

Policy information about availability of data

All manuscripts must include a data availability statement. This statement should provide the following information, where applicable:

- Accession codes, unique identifiers, or web links for publicly available datasets
- A description of any restrictions on data availability
- For clinical datasets or third party data, please ensure that the statement adheres to our policy

The authors declare that all the data supporting the findings of this study are available within the article and Supplementary Information files. The publicly available datasets can be found in pdb id: 4HIQ [https://www.wwpdb.org/pdb?id=pdb_00004hiq] and 4DKL [https://www.wwpdb.org/pdb?id=pdb_00004dkl]. All compounds (including AG10-L1-Nal, AG10-L2-Nal, and AG10-L2-Oxy) can be obtained through a standard material transfer agreement by contacting malhamadsheh@pacific.edu. Source data are provided with this paper.

Field-specific reporting

Please select the one below that is the best fit for your research. If you are not sure, read the appropriate sections before making your selection.

☒ Life sciences ☐ Behavioural & social sciences ☐ Ecological, evolutionary & environmental sciences

For a reference copy of the document with all sections, see nature.com/documents/nr-reporting-summary-flat.pdf

Life sciences study design

All studies must disclose on these points even when the disclosure is negative.

Sample size	A priori analysis revealed that to achieve a statistical power of 0.8 with alpha of 0.05 and small s.d. values, the number of animals per group has to be more than 2. Please see the table and figure legends for study-specific sample size. For in vitro studies, sample size n=3 was determined to be sufficient owing to low observed variability between samples.
Data exclusions	No data was excluded from the studies.
Replication	Data were replicated for the studies (minimum n=3). Please see the table and figure legends for study-specific details.
Randomization	For all in vivo studies, such as blood-brain barrier (BBB) penetration, pharmacokinetics, and gastrointestinal transit studies, the rats were randomized among the treatment groups. For the hot plate latency tests, pre-dose baseline latencies were ranked, and the animals were randomized to treatment groups, so the mean baseline latencies were similar among groups. For the in vitro experiments, all the samples were treated equally and thus, there was no requirement for randomization.
Blinding	For the in vivo experiments, the experimenters were blinded to the treatment groups. For other experiments, blinding is not necessary because no bias could be made by the subject or tester.

Reporting for specific materials, systems and methods

We require information from authors about some types of materials, experimental systems and methods used in many studies. Here, indicate whether each material, system or method listed is relevant to your study. If you are not sure if a list item applies to your research, read the appropriate section before selecting a response.

Materials & experimental systems

n/a	Involved in the study	
☐	☒	Antibodies
☐	☒	Eukaryotic cell lines
☒	☐	Palaeontology and archaeology
☐	☒	Animals and other organisms
☒	☐	Human research participants
☒	☐	Clinical data
☒	☐	Dual use research of concern

Methods

n/a	Involved in the study	
☒	☐	ChIP-seq
☒	☐	Flow cytometry
☒	☐	MRI-based neuroimaging

Antibodies

Antibodies used	1. Rabbit anti-RBP4 antibody was purchased from Abcam (#ab154914).<br>2. IRdye800 donkey antirabbit secondary antibody was bought from LI-COR Biosciences (#926-32213).
Validation	1. https://www.abcam.com/rbp4-antibody-ab154914.html

2. https://www.licor.com/bio/reagents/irdye-800cw-donkey-anti-rabbit-igg-secondary-antibody

Eukaryotic cell lines

Policy information about cell lines

Cell line source(s)	Jurkat (#TIB-152TM) and Hep3B (#HB-8064™) cell lines were purchased from ATCC.
Authentication	None of the cell lines have been authenticated after purchase.
Mycoplasma contamination	Cell lines were not tested for mycoplasma contamination.
Commonly misidentified lines (See ICLAC register)	None of the used cell lines is listed in the ICLAC database.

Animals and other organisms

Policy information about studies involving animals; ARRIVE guidelines recommended for reporting animal research

Laboratory animals	Approximately 6-8 weeks old non-cannulated and jugular vein/intracerebroventricular (ICV) cannulated male Sprague-Dawley rats (226-250 g each, strain code: 001) from Charles River Laboratories Inc., Hollister, CA, USA were used for various animal studies.
Wild animals	No wild animals were used in this study.
Field-collected samples	No field-collected samples were used in this study.
Ethics oversight	Pre- and post-operative care for the animals ordered from Charles River were performed according to the Charles River Institutional Animal Care and Use Committee (IACUC) policy. The animal studies performed at University of the Pacific were conducted in accordance with National Institutes of Health (NIH) guidelines for the care and use of live animals and were approved by the Institutional Animal Care and Use Committee (IACUC) at University of the Pacific. The IACUC protocol 19R02 and 21R03 were used in this study.

Note that full information on the approval of the study protocol must also be provided in the manuscript.
